# Identification of closely related species in *Aspergillus* through Analysis of Whole-Genome

**DOI:** 10.3389/fmicb.2024.1323572

**Published:** 2024-02-21

**Authors:** Guihong Qi, Lijun Hao, Yutong Gan, Tianyi Xin, Qian Lou, Wenjie Xu, Jingyuan Song

**Affiliations:** ^1^Key Lab of Chinese Medicine Resources Conservation, State Administration of Traditional Chinese Medicine of the People's Republic of China, Institute of Medicinal Plant Development, Chinese Academy of Medical Sciences, Peking Union Medical College, Beijing, China; ^2^Engineering Research Center of Chinese Medicine Resource, Ministry of Education, Beijing, China

**Keywords:** Analysis of whole-GEnome, *Aspergillus*, species identification, invasive aspergillosis, sequencing, genome editing

## Abstract

The challenge of discriminating closely related species persists, notably within clinical diagnostic laboratories for invasive aspergillosis (IA)-related species and food contamination microorganisms with toxin-producing potential. We employed Analysis of the whole-GEnome (AGE) to address the challenges of closely related species within the genus *Aspergillus* and developed a rapid detection method. First, reliable whole genome data for 77 *Aspergillus* species were downloaded from the database, and through bioinformatic analysis, specific targets for each species were identified. Subsequently, sequencing was employed to validate these specific targets. Additionally, we developed an on-site detection method targeting a specific target using a genome editing system. Our results indicate that AGE has successfully achieved reliable identification of all IA-related species (*Aspergillus fumigatus, Aspergillus niger, Aspergillus nidulans, Aspergillus flavus*, and *Aspergillus terreus*) and three well-known species (*A. flavus, Aspergillus parasiticus*, and *Aspergillus oryzae*) within the *Aspergillus* section. *Flavi* and AGE have provided species-level-specific targets for 77 species within the genus *Aspergillus*. Based on these reference targets, the sequencing results targeting specific targets substantiate the efficacy of distinguishing the focal species from its closely related species. Notably, the amalgamation of room-temperature amplification and genome editing techniques demonstrates the capacity for rapid and accurate identification of genomic DNA samples at a concentration as low as 0.1 ng/μl within a concise 30-min timeframe. Importantly, this methodology circumvents the reliance on large specialized instrumentation by presenting a singular tube operational modality and allowing for visualized result assessment. These advancements aptly meet the exigencies of on-site detection requirements for the specified species, facilitating prompt diagnosis and food quality monitoring. Moreover, as an identification method based on species-specific genomic sequences, AGE shows promising potential as an effective tool for epidemiological research and species classification.

## Introduction

*Aspergillus* consists of over 340 officially recognized species based on their morphology, physiology, and phylogenetic traits, with implications across various fields such as food production, biotechnology, environmental science, and human health (Samson et al., 2014). Notably, *Aspergillus fumigatus* is a potentially lethal human pathogen and a major allergen (Goffeau, 2005). It has garnered attention for its association with allergic reactions and invasive syndromes (Hope et al., 2005). Invasive aspergillosis (IA) presents diverse clinical manifestations and, unfortunately, remains associated with a high mortality rate. Early diagnosis is pivotal for a favorable outcome, but current methods pose challenges. Additionally, aflatoxins, highly carcinogenic metabolites produced by *Aspergillus flavus* and *Aspergillus parasiticus*, pose universal agricultural and health concerns (Thakare et al., 2017). Conversely, species such as *Aspergillus nidulans, Aspergillus niger*, and *Aspergillus oryzae* are generally recognized as safe (GRAS) by the US FDA for industrial use, meeting human safety requirements (Li et al., 2022). Given the economic importance of the genus *Aspergillus*, practical concerns arise regarding the precision and stability of its identification (Geiser et al., 2007).

Species identification is a critical focus in biological research due to its pivotal roles in diagnostic, taxonomic, epidemiological, medical, and socio-economic aspects (Li et al., 2004). Before the advent of DNA sequencing, a reliance on phenotypic tests was necessary (Tian et al., 2020), but morphological and physiological traits are often inconsistent, even within the same species (Geiser et al., 2007). Traditional clinical laboratory identification based on morphology is limited to the section or species-complex level for *Aspergillus*, making it challenging to achieve species-level identification (Gautier et al., 2016). DNA barcoding, particularly utilizing the ITS region, has become widely employed for fungal identification (Fernandes et al., 2021; Chen et al., 2023). However, due to limited polymorphism, secondary markers such as partial β*-tubulin* or calmodulin are needed for confident species identification, albeit within a narrow taxonomic range (Geiser et al., 2007; Schoch et al., 2012; Gautier et al., 2016). Despite advancements, accurate species identification in *Aspergillus* remains challenging, necessitating the development of innovative methods.

Genome sequencing is a fundamental tool for unraveling the unique biological characteristics and evolutionary history of a species, providing insights into physiology, ecology, and evolution (Alneberg et al., 2018). *Aspergillus*, the most genome-sequenced fungal genus, surpasses even *Saccharomyces* and *Candida yeasts* (Dujon, 2010). Various fungal databases, such as FungiDB, JGI MycoCosm, and Ensembl Fungi, along with public repositories such as the National Center for Biotechnology Information (NCBI), European Nucleotide Archive (ENA), and DNA Data Bank of Japan (DDBJ) (Meyer et al., 2020), host a wealth of fungal genome sequences. To date, 1,164 genomes have been published on NCBI (https://www.ncbi.nlm.nih.gov/data-hub/genome) for most vital species. *Aspergillus terreus* has the smallest known genome (0.1385 Mb) in *Aspergillus*, while *Aspergillus latus* boasts the largest (77.56 Mb). With the ongoing reduction in sequencing costs and advances in technology (Gao et al., 2023), sequencing a species' genome now costs less than $1, making it easily accessible. This accessibility has facilitated the sequencing of multiple strains within the same species, as exemplified by *A. fumigatus*, with over 300 published genome versions. Despite the wealth of information generated, genome sequencing for *Aspergillus* remains a powerful tool for accurate species-level classification.

The recent rapid advances in genomic and bioinformatic analysis have greatly boosted the development of biological sciences and technologies. A team conducted genome-wide comparisons of gene similarity in ~2,000 fully sequenced genomes (Lan et al., 2016). With the help of full-genome sequence analysis, researchers confirmed that the closest species to *Enterobacter aerogenes* is *Klebsiella pneumoniae* (Diene et al., 2013; Davin-Regli et al., 2019). Cross-species sequence comparison is considered a powerful approach to analyzing functional sites in genomic sequences, and many discoveries have been made based on genomic alignments (Brudno et al., 2004). The whole genome has significantly contributed to understanding the species-specificity of *Aspergillus*, making it an ideal choice for addressing fundamental questions in natural history, systematics, molecular genetics, development, and natural product chemistry (Gibbons and Rokas, 2013). Whole genome analysis has been proven to achieve accurate species identification (Hao et al., 2022; Song et al., 2022), but there is no evidence yet of its identification capabilities in closely related species.

The rapid accumulation of sequenced genomes in *Aspergillus*, particularly with various pathogenic or model species, positions it as an ideal candidate for Analysis of the whole-GEnome (AGE). In our study, we constructed species-level-specific target sequences for 77 *Aspergillus* species with published whole genomes, encompassing almost all widely studied species within the genus *Aspergillus*. Leveraging this resource, we subsequently employed a comparative analysis approach, comparing each experimental sample to the reference genomes, to extract species-specific sequences. Among them, *A. fumigatus*, the most important clinically pathogenic fungus, was used to discuss the methodological approach for AGE. In addition, we specifically focused on *A. flavus, A. parasiticus*, and *A. oryzae*, which are closely related and of significant economic importance. Our study addresses the challenge of *Aspergillus* species identification by constructing specific target sequence libraries for 77 *Aspergillus* species, offering a practical and viable solution to the problem of accurate *Aspergillus* species identification.

## Materials and methods

### Sequences download

The genome accession numbers of these 77 *Aspergillus* species, along with their corresponding strains, submitting institutions, and published references, are all presented in Supplementary Table S1. Moreover, the sequences of ITS, *BenA*, and CaM barcode reference sequences of these 77 *Aspergillus* species, along with related information, were downloaded as a query sequence library from the NCBI nucleotide database (Supplementary Table S2).

### Standard strains

Despite extensive efforts, we were only able to procure standard strains for a subset of seven species among them. The strains of *A. fumigatus, A. flavus, A. oryzae, A. niger, A. nidulans, A. parasiticus*, and *A. terreus* were obtained from the Shanghai Bioresource Collection Center (SHBCC, Shanghai, China).

### DNA barcoding for *Aspergillus* species

#### Genomic DNA extraction

The samples were finely ground in liquid nitrogen, and DNA extraction was performed using a commercial genomic DNA extraction kit [DP305, Tiangen Biotech (Beijing) Co. Ltd., China] according to the manufacturer's protocols. Then, the DNA quality and quantity were evaluated using a Nanodrop 2000 spectrophotometer (Thermo Fisher Co. Ltd., United States) and 0.8% agarose gel electrophoresis in 1**×** TAE buffer at 140 V for 40 min (Bio Rad Laboratories Inc., United States).

#### DNA amplification and sequencing

The following primers were used for PCR amplification (Houbraken et al., 2020). PCR amplification of ITS, *BenA*, and *CaM* regions was performed in 25 μl reaction mixtures containing 20–100 ng of genomic DNA template, 12.5 μl of 2 × Taq PCR MasterMix (Aidlab Biotechnologies Co., Ltd.), and 1 μl of each forward and reverse primer. Samples were amplified in an Applied Biosystems Veriti™ Thermal Cycler (Thermo Fisher Scientific Inc., United States). The PCR mixtures were first heated at 95°C for 10 min, then heated for 40 cycles at 95°C for 30 s, at 56°C for 30 s, and at 72°C for 30 s, and finally incubated at 72°C for 10 min in an automated thermal cycler (GeneAmp PCR System 9700; Applied Biosystems). Purified PCR products were sequenced on an ABI 3730XL sequencer using amplification primers.

#### Data analysis

Proofreading and contig assembly of the sequencing peak diagrams were performed using the CodonCode Aligner (CodonCode Co., United States). Sequence alignment was performed using MEGA 7.0.

### Analysis of the whole-GEnome (AGE) for *Aspergillus* species

#### Bioinformatics analysis for the genomes of *Aspergillus* species

First, the genome sequences of 77 *Aspergillus* species were downloaded from the NCBI database (https://www.ncbi.nlm.nih.gov); genome reference versions are listed in Supplementary Table S1. The bioinformatics analysis procedures were primarily developed based on published tools. The genomes of these species were cut into 25-bp fragments using Jellyfish (v1.1.12) (Marçais and Kingsford, 2011) to generate (L – 25 + 1) 25-mers with the copy number using the default parameters (*L* = genome length). The 25-mers containing PAM sequences (TTTV at the beginning, where V = G, C, or A) were extracted using the awk script. Subsequently, the sequences were compared to the genomes themselves using Bowtie (v1.1.0) (Langmead et al., 2009) with default parameters to determine their locations within their respective genomes. For each species, all 25-mers were aligned to the entire genomes of other species, and sequences with at least three nucleotide differences from other sequences were selected to form a target library for species identification.

#### Sequencing for target recognition

Genomic DNA was extracted as above. For species with standard strains, we designed specific primer pairs based on the 500-bp length upstream and downstream of each target and amplified the targets using corresponding primers. The genomic DNA of each species, flanked with primers listed in Supplementary Table S3, was used as a template for PCR amplification. The primers used were synthesized by Genscript (Genscript Co. Ltd., China). PCR amplification was performed in 25 μl reaction mixtures containing 30 ng of genomic DNA, 12.5 μl of 2**×** Taq PCR MasterMix (Aidlab Biotechnologies Co. Ltd., China), and 1 μl of each forward and reverse primer (2.5 μmol/L). Samples were amplified in an Applied Biosystems Veriti™ Thermal Cycler (Thermo Fisher Co. Ltd., United States). The reaction conditions were as follows: 5 min at 94°C, followed by 35 cycles of 1 min at 94°C, 1 min at 50°C, and 1.5 min + 3 s/cycle at 72°C, with a final step of 7 min at 72°C. The amplified DNA was purified according to the instructions of the Qiaquick PCR Purification Kit (Qiagen, Co. Ltd., Germany).

The purified PCR products of each species were sequenced bidirectionally using Sanger sequencing. Contig assembly and the generation of consensus sequences were performed using CodonCode Aligner (CodonCode Co., United States). Low-quality sequence data and primer sequences were removed.

#### One-tube system for on-site target recognition

Enzymatic Recombinase Amplification (ERA) combined with the CRISPR Cas12a system enables field identification by target recognition. First, the crRNAs were designed based on the selected targets according to the references (Zetsche et al., 2015; Moreno-Mateos et al., 2017) and the information shown in Supplementary Table S3. The corresponding crRNAs for specific targets of each fungal species were synthesized (Genscript Co. Ltd., China). The ERA amplification reagents (KS101, Gendx, Co. Ltd., China) were added to the bottom of a 1.5-ml centrifuge tube. The Cas12a-crRNA complex was carefully added to the inner cap, including 2 μl Cas12a (New England Biolabs, Co. Ltd., United States), 3.3 μl crRNA, 10 μl NEBuffer 2.1, and 30.7 μl nuclease-free water. After the amplification at 37°C for 20 min, the reaction was centrifuged briefly to draw the solution from the cap to the base of the tube, and the mixture was incubated at 37°C for 10 min. Then, 4 μl of ssDNA was added to the mixture, and the final reaction mixture was incubated at 37°C and tested at 0, 3, 6, 9, 12, 15, 25, 35, 45, and 60 min. The fluorescence intensity was read at λ_ex_ 483 nm/λ_em_ 535 nm using a fluorescence microplate analyzer (Thermo Fisher Co. Ltd., United States) at each time point. A fluorescence microplate analyzer, visual fluorescence test, and lateral flow assay can also be used for the results readout. For the visual fluorescence test and lateral flow assay, the preceding procedure follows the same methodology as mentioned earlier, with the corresponding ssDNA (Supplementary Table S3). After the addition of ssDNA, incubation at 37°C for 5 min was conducted prior to the results readout.

### Statistical analyses

*P*-values were calculated using a one-way ANOVA (multiple groups). Data were expressed as mean ± SD. Differences with *P*-values < 0.05 were considered significant. All statistical analyses were performed using GraphPad Prism 8.0 software.

## Results

### AGE achieves precise identification of invasive aspergillosis*-*related species

**Figure 1:**
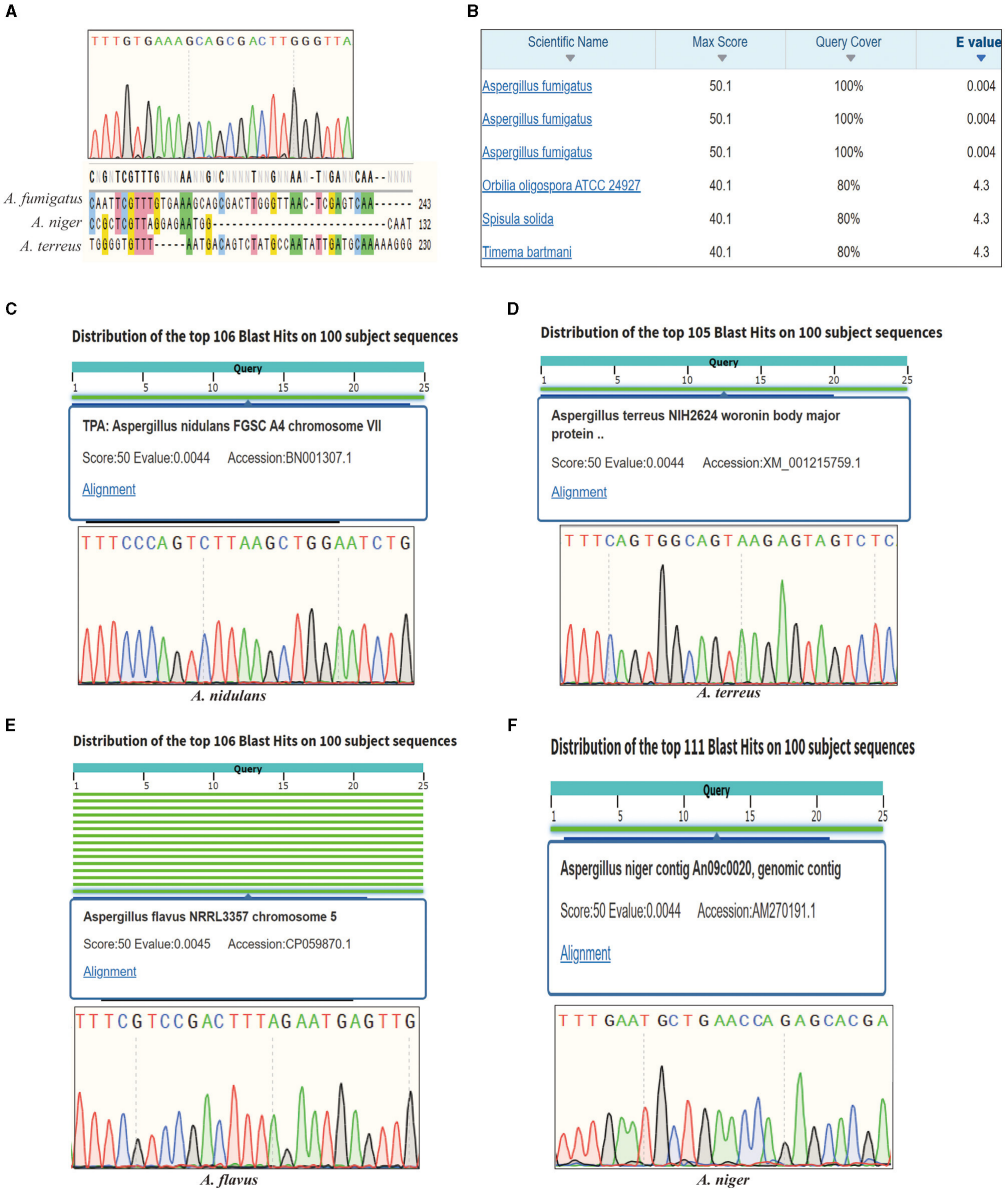
Sanger sequencing results of invasive aspergillosis-related species. **(A)** The sequencing result of *Aspergillus fumigatus* target. **(B)** The alignment of *A. fumigatus* target in the GenBank database. **(C)** The sequencing and BLAST in the GenBank database results of *Aspergillus nidulans* target. **(D)** The sequencing and BLAST in the GenBank database results of *Aspergillus terreus* target. **(E)** The sequencing and BLAST in the GenBank database results of *Aspergillus flavus* target. **(F)** The sequencing and BLAST in the GenBank database results of *Aspergillus niger* target.

Invasive aspergillosis is primarily caused by *A. fumigatus* but also includes *A. niger, A. flavus, A. terreus*, and *A. nidulans*. For *A. fumigatus*, the novel primer pairs have successfully enabled the amplification of the corresponding target, and the sequencing result confirmed this target with good sequencing quality. Moreover, the specific primer pairs exclusively amplify *A. fumigatus, A. terreus*, and *A. niger*, the fungi relevant to invasive aspergillosis, while not amplifying other fungal species. This significantly reduces the time required for an initial clinical diagnosis. Further sequencing results demonstrated that the target is unique to *A. fumigatus* and is absent in other pathogenic fungi (Figure 1A). BLAST searches revealed the high specificity of this target, as no other species were matched within a three-base pair difference (Figure 1B). Additionally, the amplicon containing the target was used to query the NCBI database, revealing intriguing findings. It appeared that this target is situated on chromosome 2 and exhibited expression in various strains of *A. fumigatus*. Conversely, the homology with other species was low, indicating the target's reliability as a specific sequence for identifying *A. fumigatus* (Supplementary Figure S1A). Notably, this discovery highlights its novelty, as this particular sequence has not been thoroughly investigated in any previous studies, particularly concerning the identification of *A. fumigatus*. For other clinical pathogenic fungi, we demonstrated that each reference target can be utilized for species-specific identification, and sequence alignment results revealed that each target can discriminate among different pathogenic fungi (Figures 1C–F). Importantly, AGE has provided novel insights into the identification of *A. niger*. The PCR amplification product of the target is 236 bp, and the sequences showed no significant similarity with other species found in the NCBI Entrez gene database by utilizing the BLAST tool (Supplementary Figure S1B). Our results also confirmed this point; only single bands were obtained after PCR amplification, and the PCR results can directly be used for *A. niger* identification. This finding highlights the extraordinary uniqueness of this target within the identification of *A. niger* research. Furthermore, in the case of *A. terreus*, the target belongs to the Woronin body major protein coding region; this finding underscores the applicability of both unannotated targets and annotated targets in species identification.

### The reference target library for 77 important *Aspergillus* species identification

Initially, we evaluated the performance of DNA barcodes in these species. Among them, the ITS barcode, as a universal barcode, exhibits the highest amplification efficiency. However, ITS was unable to achieve species-level identification for these seven species. *Aspergillus flavus* and *A. oryzae* are completely identical, making them indistinguishable (Supplementary Figure S2A). A BLAST analysis of the ITS sequences of *A. niger* showed that it could not distinguish *A. niger, Aspergillus welwitschiae*, or *Aspergillus awamori* (Supplementary Figures S2B–D). For the *BenA* barcode, it was only able to achieve species-level differentiation for *A. nidulans, A. fumigatus*, and *A. terreus*. For the *CaM* barcode, its amplification success rate is lower and it cannot achieve amplification of *A. niger, A. nidulans*, or *A. oryzae*. This makes it challenging to develop a universal DNA barcode for *Aspergillus* species identification. Next, we evaluated the effectiveness of three commonly used DNA barcodes (ITS, *BenA*, and *CaM*) for identifying these 77 *Aspergillus* species. The identification rates for *Aspergillus* using ITS, *BenA*, and *CaM* barcodes were 11.7%, 49.3%, and 53.2%, respectively (Supplementary Figure S2E). All the results verified by DNA barcoding faced challenges in identifying species-level classification within the genus *Aspergillus*.

**Figure 2:**
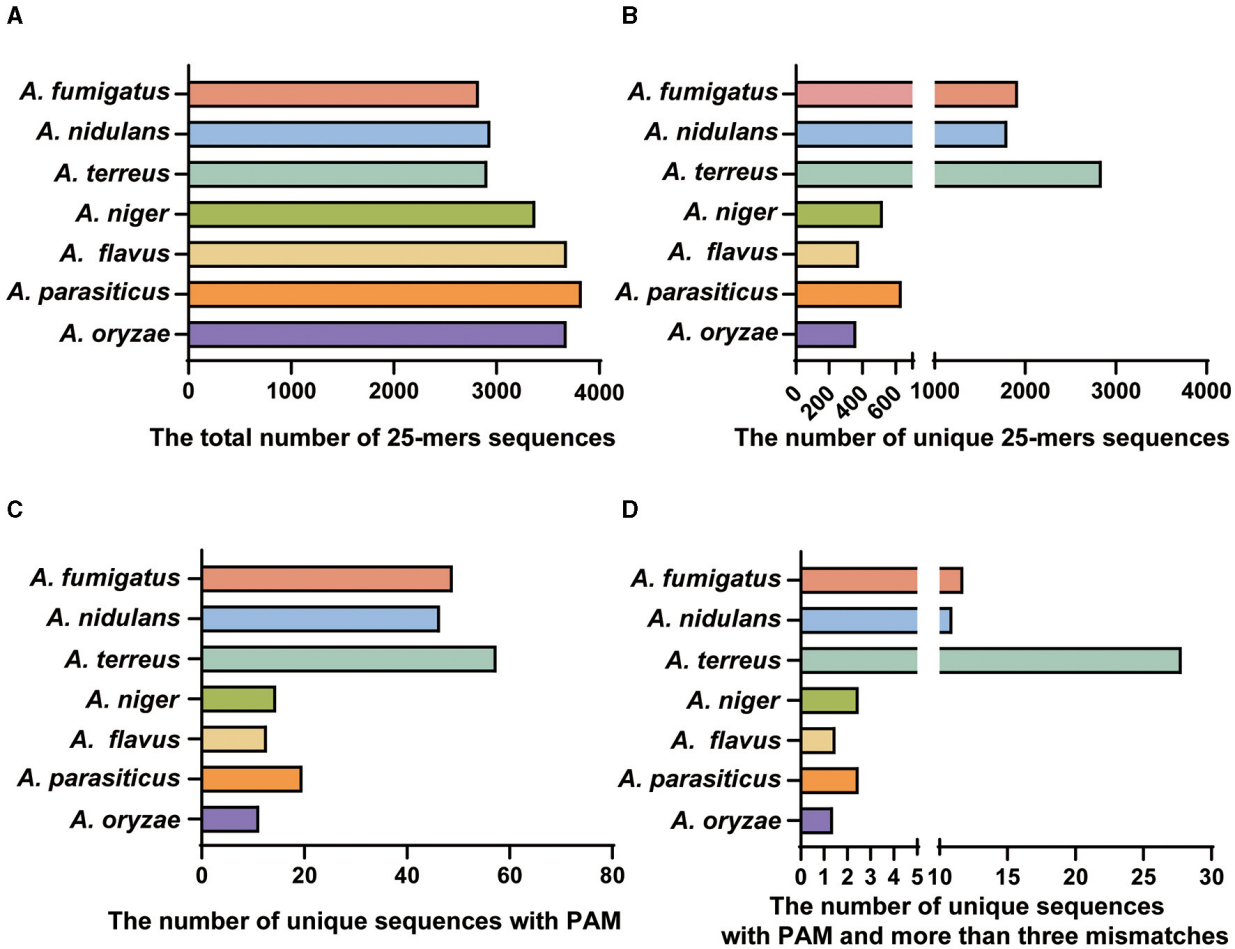
The bioinformatics results for each step. **(A)** The total number of 25-mers sequences of the seven important species. **(B)** The number of unique 25-mers sequences for species after comparison with the other 76 species. **(C)** The number of unique sequences with a PAM. **(D)** The number of unique sequences with a PAM and more than three mismatched (specific targets) species after comparison with the other 76 species.

For the seven important species, the bioinformatics analysis revealed that the genome sizes of these species were similar, with a concentrated number of 25-mer deduplicated sequences ranging from 29 to 35 million (Figure 2A). However, when closely related species were included in the whole genome analysis, the number of specific sequences obtained by related species decreased significantly (Figures 2B, C). Most notably, the three species of the *Flavi* subgenus had the lowest number of specific targets (Figure 2D). Similarly, we found the specific target library for other species and randomly selected a reference target from the resulting specific target library (Table 1). For the reference target for *Aspergillus* species identification, the specificity was verified within the NCBI database by performing a BLAST analysis with the target and 500-bp length upstream and downstream of each target.

**Table 1:** The specific targets for *Aspergillus* species.

**Species**	**Reference target (5′ → 3′)**
*Aspergillus terreus*	TTTCAGTGGCAGTAAGAGTAGTCTC
*Aspergillus eucalypticola*	TTTCCAGGCGAGATGCAAAATCCGG
*Aspergillus bombycis*	TTTGACGCCTCTAATGTAGAATGAG
*Aspergillus candidus*	TTTATTATGCGTAGCACACTGCTAT
*Aspergillus oerlinghausenensis*	TTTAGCCTAGGGGCCATGGTTCTAA
*Aspergillus oryzae*	TTTCCATTTACTTTTAAGGCAGGGG
*Aspergillus versicolor*	TTTCGGGCCATTTTGAAAGCCGTTT
*Aspergillus wentii*	TTTCGCACGATAAGTGCATCCAAAA
*Aspergillus novoparasiticus*	TTTGGGAAACACCTTCTTGAATTGC
*Aspergillus awamori*	TTTCGTGGAGCCAAACTATACTTTC
*Aspergillus nidulans*	TTTCCCAGTCTTAAGCTGGAATCTG
*Aspergillus heteromorphus*	TTTCTGCCTCATCAATGCGGAGGGC
*Aspergillus fumigatus*	TTTGTGAAAGCAGCGACTTGGGTTA
*Aspergillus parasiticus*	TTTGCTAAAAGGCCATTGAGGCGAG
*Aspergillus aculeatinus*	TTTAGAAGGATACTTCGTGCATGAA
*Aspergillus pseudotamarii*	TTTCTGGCGTGTTTACAACACTCCG
*Aspergillus caelatus*	TTTCAACAAAGTTTCCACGGTCAAG
*Aspergillus sojae*	TTTCTCATATACCCAGTTGAGTATT
*Aspergillus udagawae*	TTTCTCAGTGTATACAGTGGAAGGC
*Aspergillus welwitschiae*	TTTCGGTGTGGTGAGTGTGACACCC
*Aspergillus carbonarius*	TTTATATTAGTGCCGATGAGGGAGA
*Aspergillus phoenicis*	TTTCTCCATTAACTCGTTGTTTTGT
*Aspergillus thermomutatus*	TTTCACTTCTACCTGGCGACCTTCA
*Aspergillus brunneoviolaceus*	TTTCCTGGCGGTGCAGAGACAGTGC
*Aspergillus leporis*	TTTCAACAAATGCCGAAACCCTCTA
*Aspergillus pseudoviridinutans*	TTTAAGTCCTGGGCGCGCCGGAACG
*Aspergillus vadensis*	TTTGAGGAGTGACGATACTAACAAC
*Aspergillus protuberus*	TTTCTGACACCTTGTATAGCTTCAG
*Aspergillus tamarii*	TTTCCCCATGGGACGCAAAGAGATA
*Aspergillus glaucus*	TTTGAACGACTGGCTTCATTGGGCG
*Aspergillus steynii*	TTTGACACTGGCTACGTCAGCCAGA
*Aspergillus jensenii*	TTTATGACACCGTCATTGGTAGTTC
*Aspergillus costaricensis*	TTTGTTAGTGTCCAGTCCCTGGAAT
*Aspergillus sclerotiicarbonarius*	TTTAATTCTGTAATTGCTAGATATG
*Aspergillus puulaauensis*	TTTCAGGCTTGCTCCATGACACGAA
*Aspergillus minisclerotigenes*	TTTCACAAAATAGGTTCTTGGTGCA
*Aspergillus fijiensis*	TTTACACCTAACAATGTATGCGAAG
*Aspergillus felis*	TTTAGGATAGCGTTAGGGGTAGAGT
*Aspergillus aculeatus*	TTTGAGCGTGGTGAACCAAGTGGTG
*Aspergillus homomorphus*	TTTCAATGGTGCTGATAAACGGATT
*Aspergillus quadrilineatus*	TTTAATAAGTCAGGAATAATAGTTT
*Aspergillus viridinutans*	TTTCAGTTGAACTCGTTTTGCCTGC
*Aspergillus chevalieri*	TTTGTATCGATATCGTAATTTACAG
*Aspergillus sydowii*	TTTATATAGCCCCCGTTGGAGCGGC
*Aspergillus ruber*	TTTATCTGATGCTGGTCATCTAGTG
*Aspergillus uvarum*	TTTGTGGTATTACCTCGTATACTAG
*Aspergillus arachidicola*	TTTCTTATCCTACAACCCATTACTC
*Aspergillus clavatus*	TTTGGCGTGCTGTTCGTAATTCAAC
*Aspergillus novofumigatus*	TTTCAGAAAAACCAATGCAGGTCAC
*Aspergillus spinulosporus*	TTTCGTAGGACGGTTTGGAGAGAGA
*Aspergillus niger*	TTTGAATGCTGAACCAGAGCACGAT
*Aspergillus melleus*	TTTACGAAAGCCATAAAATCTTTTA
*Aspergillus neoniger*	TTTAAACAGGTAGTGTTAGTAGTGA
*Aspergillus brasiliensis*	TTTCGCGTTAAGAAGCAGACCCTCC
*Aspergillus fumigatiaffinis*	TTTGAGAATCCAGCGGAGCTAATCC
*Aspergillus saccharolyticus*	TTTCCATGCATAGTGCGCAGCACGC
*Aspergillus japonicus*	TTTGCTATGACCCTGCACGTAGAGG
*Aspergillus pseudonomiae*	TTTGAACGTAGCGGTGAACGCGAAC
*Aspergillus flavus*	TTTCGTCCGACTTTAGAATGAGTTG
*Aspergillus alliaceus*	TTTCGGGTAGCCCCTGGGATGATCC
*Aspergillus hiratsukae*	TTTCCCTTGTCAATCCGGTAAACCG
*Aspergillus amoenus*	TTTCTCGCGGCCTAATGATTGACTA
*Aspergillus lentulus*	TTTCTGATTGTACCCGCCGTTTTAG
*Aspergillus nomiae*	TTTAATTGAGTGAGTTTCTTTGGGT
*Aspergillus ochraceus*	TTTCCTTTGACTGGGGTTGCGATCT
*Aspergillus cristatus*	TTTGAGACAGCTTTACGAAGGTTCT
*Aspergillus ibericus*	TTTATGTAGTGCCCTGCATGACAGA
*Aspergillus tubingensis*	TTTCGGTTACTAACAAGTTACTATA
*Aspergillus fischeri*	TTTGATGAGACCCCGCTTTCGTTCA
*Aspergillus piperis*	TTTGAAGAAAGTAGTTAGTTAATGC
*Aspergillus tanneri*	TTTAGAAACTCAGGTGCATCAGGCG
*Aspergillus luchuensis*	TTTACGGCTAAAAAGATATCGTTGT
*Aspergillus mulundensis*	TTTGGGCTGTGCACGAAGCCAACCT
*Aspergillus sclerotioniger*	TTTGAAAAGGTACTTGGCCGTGCGC
*Aspergillus ustus*	TTTCAGCGCCACACATTAGAGATCT
*Aspergillus sergii*	TTTGTTTTACAAAACCACAGAAGTC
*Aspergillus calidoustus*	TTTGCCCCAATGATACCGGTATCAT

### Reference targets empower the precise identification of closely related species

**Figure 3:**
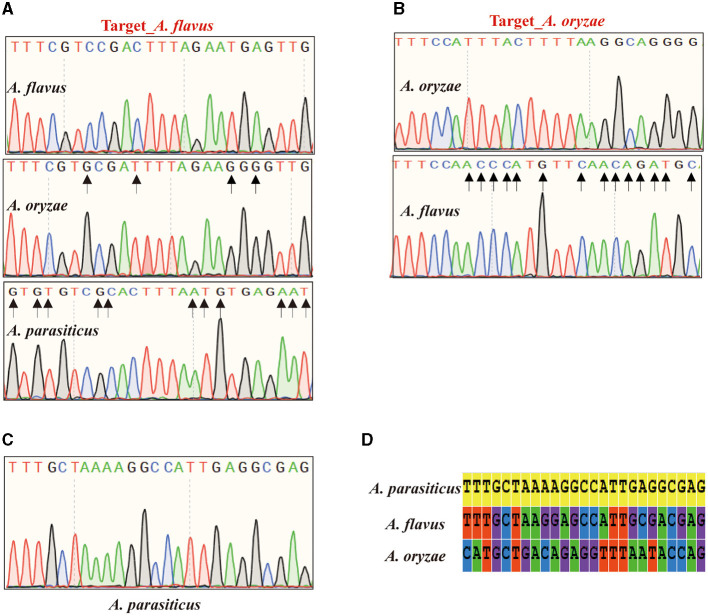
Sanger sequencing results of the *Aspergillus* section *Flavi*. **(A)** The sequencing result of *Aspergillus flavus* target. **(B)** The sequencing result of *Aspergillus oryzae* target. **(C)** The sequencing result of *Aspergillus parasiticus* target. **(D)** The alignment for *A. parasiticus* target sequence with the amplicons from *A. flavus* and *A. oryzae*.

In experimental validation, in addition to the aforementioned clinical pathogenic fungi, we successfully resolved the taxonomic challenge of discriminating between three closely related and morphologically indistinguishable species within the *Aspergillus* section *Flavi*. Among them, *A. oryzae*, as the closest relative to *A. flavus*, demonstrated the utility of *A. flavus* targets, confirming the reliability of the reference targets. For the reference target of *A. flavus*, sequencing analysis demonstrated the absence of an identical sequence to the target of *A. flavus* in *A. oryzae*, with the most similar sequence exhibiting four nucleotide variations (Figure 3A). Similarly, the target of *A. oryzae* was uniquely present in its corresponding species, while homologous sequences in *A. flavus* exhibited 14 nucleotide variations in the nucleotide sequence over a large fragment (Figure 3B). *Aspergillus parasitic*, as another closely related species within the *Aspergillus* section *Flavi*, can also be distinguished from *A. flavus* and *A. oryzae* using the specific target (Figures 3C, D). Importantly, AGE has provided novel insights into the identification of the whole genome. The PCR amplification product of *A. flavus* target is 450 bp, and BLAST alignments showed that the target is located on chromosome 5 without any annotated detail. For the target amplicon sequences of *A. oryzae* and *A. parasitic*, a similar situation was observed.

### AGE enables rapid, accurate, and on-site species identification

**Figure 4:**
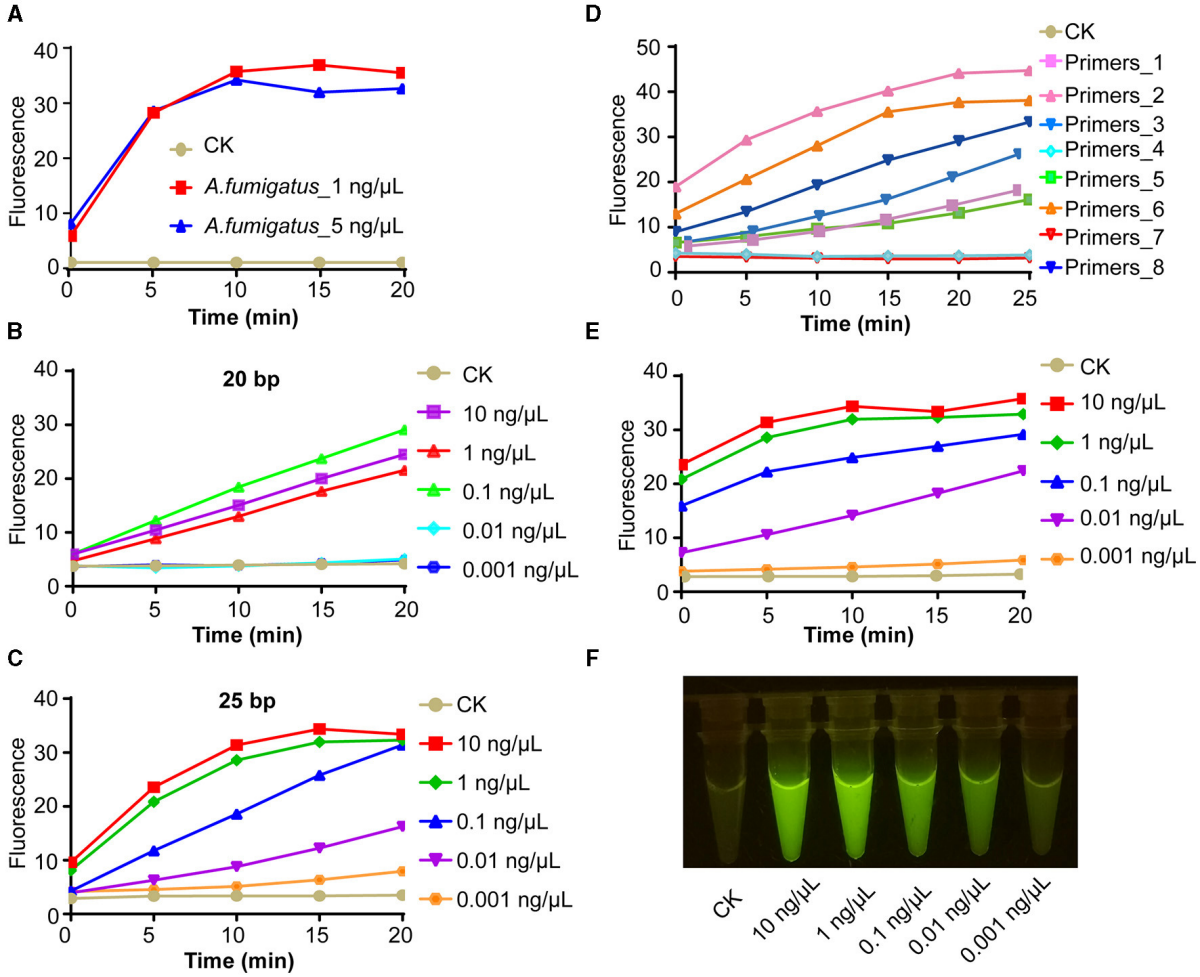
AGE enables rapid, accurate, and on-site species identification. **(A)** The fluorescence test of the amplicons. CK group contains all reagents except the DNA substrate. *Aspergillus fumigatus_*1 ng/μl contains 1 ng/μl purified target amplicons of *A. fumigatus*. *A. fumigatus_*5 ng/μl contains 5 ng/μl purified target amplicons of *A. fumigatus*. **(B)** The concentration sensitivity of 20 bp primer pairs by fluorescence over time. **(C)** The concentration sensitivity of 25 bp primer pairs by fluorescence over time. **(D)** The efficiency of different primer pairs with 25 bp. **(E)** The concentration sensitivity of the most efficient primer pairs by fluorescence over time. **(F)** The sensitivity of visual fluorescence.

To cater to the accessibility of on-site systems for *Aspergillus* species identification, particularly for the timely clinical diagnosis of pathogenic fungi and food safety detection, we conducted methodological research on the system using *A. fumigatus* as the study subject. Initially, we successfully accomplished target recognition by utilizing CRISPR Cas12a along with crRNA that specifically matched the 1 ng/μl purified genomic DNA product (Figure 4A). Next, we replaced PCR amplification with room-temperature isothermal amplification. For on-site identification, it comprised two parts: the specific amplification of the target was conducted at the bottom of a 1.5-ml centrifuge tube, with the CRISPR Cas12a system components added to the tube cap. For one-tube systems, we first investigated the influence of primer length on the intensity of the final fluorescence signal. Compared with 20-bp primers, 25-bp primers have better amplification efficiency and AGE sensitivity can reach 0.01 ng/μl (Figures 4B, C). Afterward, we obtained further confirmation that utilizing different primers of identical length can yield completely disparate outcomes. Our results showed this, thereby underscoring the pivotal role of primer design in ensuring the success of the experiment (Figure 4D). When we employed the most efficient primer pair, we confirmed that the sensitivity of AGE was 0.01 ng/μl (Figure 4E). The fluorescent visualization test showed the sensitivity of AGE was also able to reach 1 ng/μl (Figure 4F).

### AGE successfully identified closely related species on-site identification

**Figure 5:**
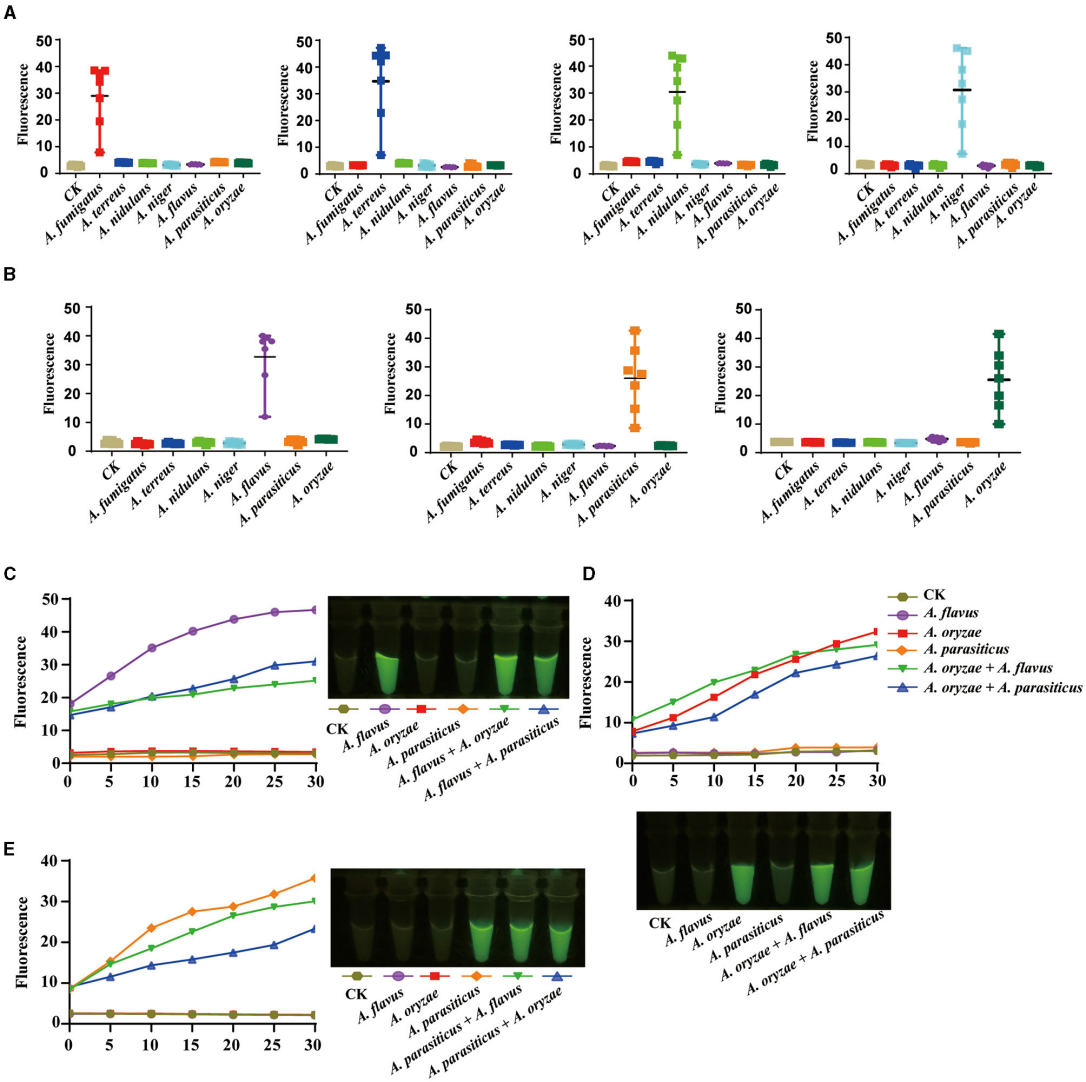
The on-site identification of closely related species in the genus *Aspergillus*. **(A)** The identification of invasive aspergillosis-related species. **(B)** The identification of toxin-producing *Flavi* species. **(C)** The identification of *Aspergillus flavus* and its mixture. **(D)** The identification of *Aspergillus oryzae* and its mixture. **(E)** The identification of *Aspergillus parasiticus* and its mixture.

The selected species belong to the *Aspergillus* genus and are closely related, containing all the species capable of causing *invasive aspergillosis* and the most common toxin-producing species strains. Although the accuracy of the target sequences of these species has been confirmed by sequencing, it is more necessary to solve the field detection problems of these species that are closely related to human life and health. Hence, we further investigated the response of each target sequence to all samples. The results revealed that each target sequence exclusively produced fluorescent signals in the corresponding species' sample set. No significant fluorescent signals were observed in samples from other species. Each target successfully distinguished among these closely related species, demonstrating the scientific validity and practical utility of the genomic analysis strategy (Figure 5A).

The identification of toxigenic species or specific species within these complexes is of particular importance, as fungi commonly exist in diverse fungal communities. In our samples, we included *A. flavus*, a toxigenic species, and its non-toxigenic relative, *A. oryzae*, as an illustrative example to showcase the potential of our approach in identifying complex mixtures. The results showed that whether employing fluorescence absorption detection or direct visual fluorescence observation, the target specifically indicated the presence of *A. flavus* while showing no fluorescence signal for *A. oryzae* and *A. parasiticus*. Importantly, even in mixed samples containing both *A. flavus* and *A. oryzae* or *A. parasiticus*, AGE successfully detected the fluorescent signal emitted specifically by *A. flavus* (Figures 5B, C). AGE exhibited similar success in detecting *A. oryzae* and *A. parasiticus* in mixed samples, mirroring its performance with *A. flavus* (Figures 5D, E).

## Discussion

### The accurate species-level identification of closely related *Aspergillus* species

AGE has emerged as a breakthrough solution, offering a promising resolution to the intricate challenge of discriminating among closely related species within *Aspergillus*. *Aspergillus*, extensively studied for its medical (*A. fumigatus* and *A. terreus*), food spoilage (*A. flavus* and *A. parasiticus*), and industrial (*A. niger* and *A. oryzae*) relevance (de Vries et al., 2017), has genomic data available for 77 key species. Our study pioneers the identification of these 77 species through whole genome analysis. We have successfully established reference targets for each species and experimentally validated the accuracy of all corresponding targets obtained from the available standard strains in our study through meticulous sequencing. Sequencing, the gold standard for nucleic acid detection (Correia et al., 2015), not only determines target sequence presence but also provides insights into closely related species through BLAST analysis. High-throughput processing and tools such as MEGA (Tamura et al., 2021) enable rapid analysis of large-scale data sets. Each target was rigorously compared, confirming its efficacy for accurate species-level identification across the set of 77 species. BLAST searches demonstrated the unique nature of each reference target, showing no similarity within a three-nucleotide variation to any other species in the NCBI database, emphasizing their exceptional specificity.

The diagnosis of invasive aspergillosis (IA) remains challenging for clinical microbiology laboratories (Walsh et al., 2011). Among human pathogenic *Aspergillus* species, *A. fumigatus* is the primary causative agent, followed by *A. flavus, A. terreus, A. niger*, and *A. nidulans* (Denning, 1998; Morgan et al., 2005; Dagenais and Keller, 2009). Our results revealed innovative markers by AGE for these pathogenic fungi (Figure 2), facilitating species-level identification for prompt clinical diagnostics. While many studies focus on gene discovery, such as tef1 (elongation factor 1α), tub2 (β-tubulin), tsr1, and cdc47p (Capella-Gutierrez et al., 2014), *cyp51A* is used for the differentiation of *A. flavus* from *A. oryzae* (Nargesi et al., 2021). AGE uncovered the overlooked potential of unannotated genomic regions in species identification. Each specific target obtained through AGE demonstrates comparable discriminatory capabilities, highlighting the effectiveness of targets from unannotated genomic regions. AGE's extensive and diverse collection of specific sequences allowed us to identify novel, highly species-specific markers from the genomic ‘dark matter'. For instance, we have successfully identified a novel, highly species-specific marker derived from the genomic “dark matter” through AGE, and this target can serve as a valuable marker for the identification of *A. fumigatus* (Song et al., 2023).

### AGE facilitates the real-time detection of microorganisms

AGE, coupled with genome editing, offers robust tools for rapid clinical diagnosis, food safety testing, and environmental monitoring. For on-site detection, the primary goal is to determine the presence of the target species through visualization and accurate identification. Effective bioinformatics analysis is crucial, focusing on finding highly differentiated target sequences. The genome contains numerous informative loci for species identification (Li et al., 2020), and the key lies in how to find them based on analysis of the whole genome in an affordable and streamlined manner. We have established a criterion stating that a candidate sequence must have a minimum of three nucleotide mismatches to be considered a specific target. Additionally, when validating and confirming the selected specific sequences against BLAST, non-conserved regions are filtered out, thereby improving the reliability of the sequence for species-level identification. This is not surprising since important fungal species such as *A. fumigatus* and *A. flavus* have published the genomes of more than 100 different strains, providing ample genomic information that helps filter out potential intra-species variations and ultimately ensures the accuracy of identification. Of note, this study verified that genome annotation (Yan et al., 2018) is not necessary for AGE. While genome annotation can provide additional information about the sequences, its absence does not affect the implementation of AGE. Conversely, having a high-quality and reliable genome is crucial, and fortunately, advancements in sequencing technologies have ensured the accurate sequencing of species genomes (Tully et al., 2014).

Accurate, rapid, and visual target detection aids in diagnosing pathogenic microorganisms, ensuring food safety and health, and monitoring environmental contamination. Utilizing the room-temperature amplification and CRISPR Cas12a system, we achieved on-site identification of all available strains. Despite the multi-enzyme cascade involved in the reaction, our findings align with previous research (Mayboroda et al., 2016; Yamanaka et al., 2017), showing that regular-length primers can achieve efficient amplification, contrary to the notion that longer primers are necessary (Lobato and O'Sullivan, 2018). Therefore, we suggest that the amplification efficiency may be more closely related to the sequence characteristics of the primers. A GC content below 35% or above 60% is not recommended, and the presence of consecutive stretches of more than four GC bases should be avoided. We recommend a detection concentration of 1 ng/μl genomic DNA for convenience, and AGE's versatility is evident in achieving accurate identification even for highly similar species like *A. flavus* and *A. oryzae*. Another feature is its capability to achieve the on-site detection of degraded materials due to its amplification fragments being as short as <200 bp. Therefore, AGE tackles the challenge of real-time identification for *Aspergillus* species related to human health.

### Strengths and limitations of AGE

The most important strength of AGE is that it provides a universal solution for the accurate identification of closely related species. Traditionally, microscopy was used for the identification of fungal species. While this is the “gold standard” for many of the human relevant fungal taxa, it performed poorly in identifying species within each *Aspergillus* species complex (Balajee et al., 2009; Anees-Hill et al., 2022). Thus, species identification efforts have in recent years favored DNA-based approaches over morphology-based approaches (Vu et al., 2019). DNA barcoding has been widely employed as a primary molecular identification tool and has proven to be satisfactory in the identification of diverse species at the genus level. However, the challenge arises in distinguishing closely related species at the species level due to the fact that closely related species are frequently found in close proximity to each other and there are a limited number of variable sites within the high variability regions. In theory, AGE can be effectively applied for species identification because the whole genome data for each species contains unique characteristics that distinguish it from other organisms.

Furthermore, it simplifies species identification while meeting a wider range of demands. It capitalizes on fast and affordable DNA sequencing and the rapid accumulation of genomic data (Boon et al., 2014). Currently, many studies have relied on whole genome sequencing of samples and further filtering of annotated files to identify usable, high-variable regions for species identification (Li et al., 2020; Fu et al., 2022). In our study, we refrained from sequencing each sample and filtering high-variant regions using annotation files, despite the current affordability and rapidity of sequencing. Sequencing was deemed necessary only when the species lacked comprehensive genome data of its own. At the same time, our results have demonstrated for the first time the significant potential of genomic ‘dark matter' in species identification, an aspect that has been long overlooked. Therefore, when using AGE for species identification, it is sufficient to search for differential sequences within the whole genome, and any method capable of detecting specific sequences can be utilized in combination. This includes not only the methods of sequencing and genome editing mentioned but also other approaches like observing amplified bands for identifying *A. niger* among five IA-related species.

Regarding limitations, concerns surrounding species identification in the absence of a genome can be addressed in two ways. First, there is confidence in the cost-effective and accurate shallow sequencing that can effectively resolve this issue. Second, the rapid expansion of fungal genomes, with 3,623 fungal genomes published in 2022 (https://www.ncbi.nlm.nih.gov/datasets/genome/?taxon=4751), may indicate that such concerns are unwarranted in the near future. Moreover, AGE, as a genome analysis-based identification method, is applicable to severely degraded materials. However, it cannot address sample detection when genomic DNA is absent.

### The future of AGE

To expedite the application of AGE in species identification, further efforts can focus on the following aspects. First, the establishment of an online bioinformatics analysis platform that facilitates continuous updates and enhancements of the analysis results while also allowing public access to the specific sequences required. This platform would serve as a valuable resource for researchers and practitioners. Second, the promotion of genome sequencing in a broader range of species would provide a robust foundation for selecting specific sequences and conducting comprehensive phylogenetic analyses. Finally, for closely related species that are currently difficult to differentiate, leveraging genomic data to develop species-specific sequences would ensure the accuracy of clinical pathogenic fungal diagnosis. By addressing these areas, the application of AGE in species identification can be advanced and expanded effectively.

## Conclusion

This is the first study in which 77 closely related species of *Aspergillus* have been identified. The reference targets illustrate the large diversity found in the genus *Aspergillus* and highlight the potential for the discovery of specific sequences by AGE. The successful application of AGE in the identification of closely related species is poised to bring about a revolutionary improvement in species authentication studies.

## Data availability statement

The datasets presented in this study can be found in online repositories. The names of the repository/repositories and accession number(s) can be found in the article/Supplementary material.

## Author contributions

GQ: Data curation, Formal analysis, Investigation, Methodology, Supervision, Validation, Visualization, Writing – original draft, Writing – review & editing. LH: Writing – review & editing, Methodology, Validation. YG: Methodology, Validation, Writing – review & editing. TX: Funding acquisition, Writing – review & editing. QL: Writing – review & editing, Validation. WX: Methodology, Software, Visualization, Writing – review & editing. JS: Writing – review & editing, Conceptualization, Funding acquisition, Project administration, Resources, Supervision.
