# Supplementary material for: Identification of closely related species in Aspergillus through Analysis of Whole-Genome
Source: Front Microbiol. 2024 Feb 21;15:1323572. doi: 10.3389/fmicb.2024.1323572 (PMC10915092; doi:10.3389/fmicb.2024.1323572)
Supplement: Supplementary file 1 [file Data_Sheet_1.docx]

**Supplementary materials for**

**Identification of closely related species in *Aspergillus* through Analysis of whole-Genome**

Guihong Qi_1_^a^, Lijun Hao^a^, Yutong Gan^a^, Tianyi Xin^a^, Qian Lou^a^, Wenjie Xu^a^**^*^**, Jingyuan Song^a,b^**^*^**

^a^ Key Lab of Chinese Medicine Resources Conservation, State Administration of Traditional Chinese Medicine of the People’s Republic of China, Institute of Medicinal Plant Development, Chinese Academy of Medical Sciences & Peking Union Medical College, Beijing 100193, China.

^b^ Engineering Research Center of Chinese Medicine Resource, Ministry of Education, Beijing 100193, China.

* Co- Corresponding author Jingyuan Song and Wenjie Xu.

Email: jysong@implad.ac.cn

**
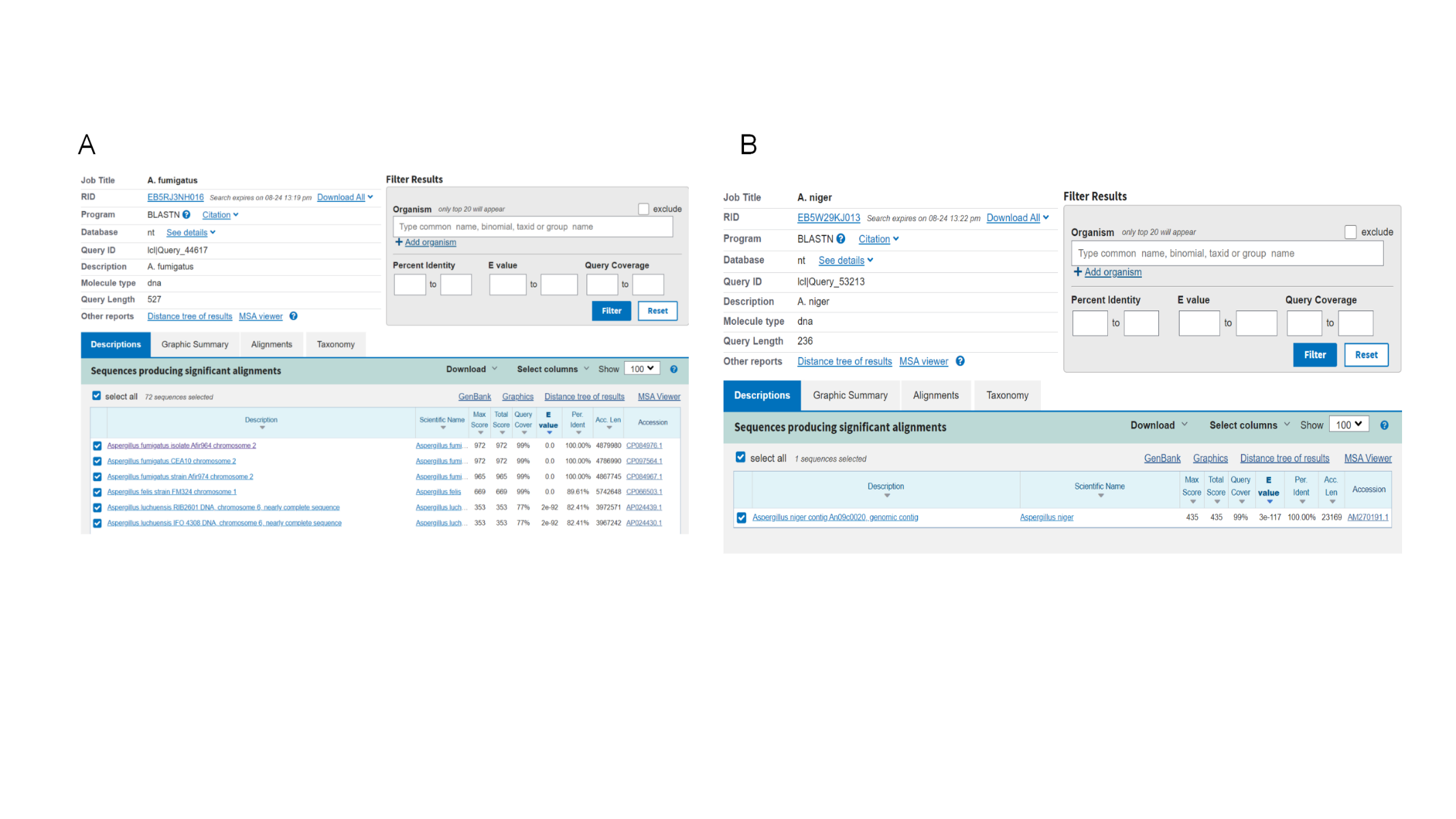
Figure S1. The alignment against the NCBI database using BLAST. (A) The alignment of *A. fumigatus* Target amplicons. (B) The alignment of *A. niger* Target amplicons.**


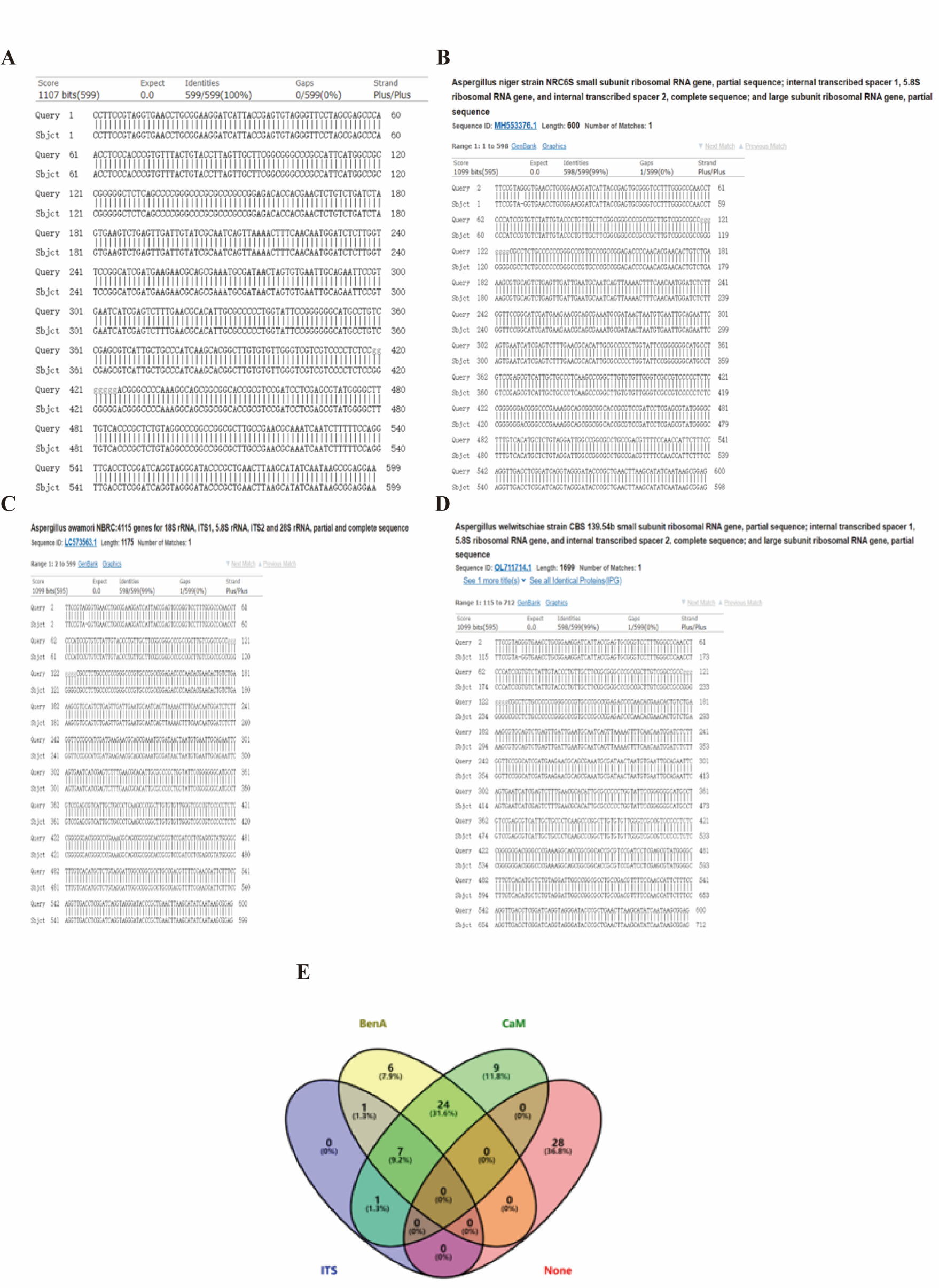

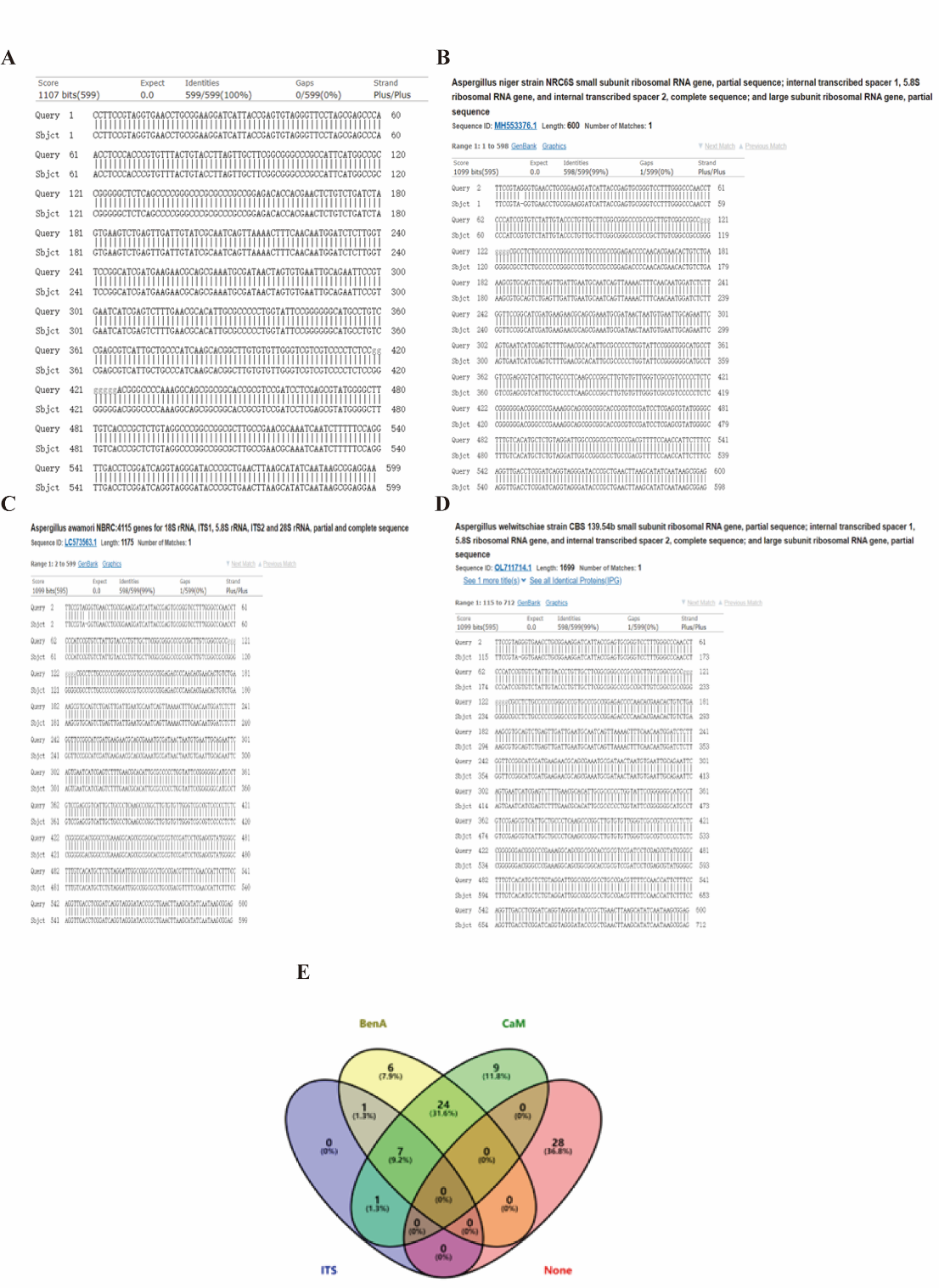
**Figure S2 The DNA barcoding performance in genus *Aspergillus*. (A) The alignment of A. flavus and A. oryzae. (B) The ITS sequence of *A. niger*. (C) The ITS sequence of *A. welwitschiae*. (D) The ITS sequence of *A. awamori*. (E) The identification efficiency of ITS, BenA, CaM for *Aspergillus* species.**

**Table S1 The information of *Aspergillus* species.**

| Species | Strain | Assembly accession | Submitter | RefSeq assembly and GenBank assembly identical | Publications |
| --- | --- | --- | --- | --- | --- |
| *Aspergillus terreus* | NIH2624 | GCA_000149615.1 | Broad Institute | Yes | Askenazi M et al., Nat Biotechnol. 2003 Feb;21(2):150-6. |
| *Aspergillus oryzae* | RIB40 | GCA_000184455.3 | National Institute of Technology and Evaluation (NITE), Japan | Yes | Machida M. et.al Nature. 2005 Dec 22;438(7071):1157-61. |
| *Aspergillus versicolor* | CBS 583.65 | GCA_001890125.1 | DOE Joint Genome Institute | Yes | n/a |
| *Aspergillus wentii* | DTO 134E9 | GCA_001890725.1 | DOE Joint Genome Institute | YES | n/a |
| *Aspergillus awamori* | IFM 58123 | GCA_003850985.1 | Medical Mycology Research Center | n/a | n/a |
| *Aspergillus nidulans* | FGSC A4 | GCA_000011425.1 | Eurofungbase | Yes | Wortman JR. et.al., Fungal Genet Biol. 2009 Mar;46 Suppl 1(Suppl 1):S2-13. |
| *Aspergillus heteromorphus* | CBS 117.55 | GCA_003184545.1 | DOE Joint Genome Institute | n/a | Vesth TC. Et.al., I Nat Genet. 2018 Dec;50(12):1688-1695. |
| *Aspergillus fumigatus* | Af293 | GCA_000002655.1 | J. Craig Venter Institute | Yes | Nierman WC. et.al Nature. 2005 Dec 22;438(7071):1151-6. |
| *Aspergillus parasiticus* | CBS 117618 | GCA_009176385.1 | DOE Joint Genome Institute | n/a | Kjærbølling I et al., Nat Commun, 2020 Feb 27;11(1):1106 |
| *Aspergillus aculeatinus* | CBS 121060 | GCA_003184765.1 | DOE Joint Genome Institute | YES | n/a |
| *Aspergillus sojae* | SMF134 | GCA_008274985.1 | Kookmin University | n/a | Kim KU. Et.al., J Microbiol. 2019 Oct;57(10):874-883. |
| *Aspergillus udagawae* | IFM 46973 | GCA_001078395.2 | Medical Mycology Research Center | No | n/a |
| *Aspergillus welwitschiae* | CBS 139.54b | GCA_003344945.1 | DOE Joint Genome Institute | YES | Vesth TC. Et.al., Nat Genet. 2018 Dec;50(12):1688-1695. |
| *Aspergillus tamarii* | CBS 117626 | GCA_009193485.1 | DOE Joint Genome Institute | n/a | n/a |
| *Aspergillus costaricensis* | CBS 115574 | GCA_003184835.1 | DOE Joint Genome Institute | Yes | n/a |
| *Aspergillus puulaauensis* | MK2 | GCA_016861865.1 | Kagoshima University | No | n/a |
| *Aspergillus minisclerotigenes* | MRI390 | GCA_028505775.1 | Max Rubner-Institut | n/a | Kjærbølling I. et.al. Nat Commun. 2020 Feb 27;11(1):1106. |
| *Aspergillus fijiensis* | CBS 313.89 | GCA_003184825.1 | DOE Joint Genome Institute | n/a | Vesth TC et al., Nat Genet, 2018 Dec;50(12):1688-1695 |
| *Aspergillus felis* | FM324 | GCA_016413765.1 | ZHEJIANG UNIVERSITY | n/a | n/a |
| *Aspergillus viridinutans* | IFM 47045 | GCA_018404265.1 | Medical Mycology Research Center | No | Takahashi H et al., Frontiers in Fungal Biology, 2021;2 |
| *Aspergillus chevalieri* | M1 | GCA_016861735.1 | Kagoshima University | n/a | Kadooka C et al., J Gen Appl Microbiol, 2020 Sep 30;66(4):239-243 |
| *Aspergillus sydowii* | CBS 593.65 | GCA_001890705.1 | DOE Joint Genome Institute | YES | n/a |
| *Aspergillus ruber* | CBS 135680 | GCA_000600275.1 | DOE Joint Genome Institute | n/a | Kis-Papo T et al., Nat Commun. 2014 May 9;5:3745. |
| *Aspergillus uvarum* | CBS 121591 | GCA_003184745.1 | DOE Joint Genome Institute | n/a | Vesth TC et al., Nat Genet, 2018 Dec;50(12):1688-1695 |
| *Aspergillus arachidicola* | CBS 117612 | GCA_009193545.1 | DOE Joint Genome Institute | n/a | Kjærbølling I et al., Nat Commun, 2020 Feb 27;11(1):1106 |
| *Aspergillus clavatus* | NRRL 1 | GCA_000002715.1 | J. Craig Venter Institute | Yes | Fedorova ND et al., PLoS Genet. 2008 Apr 11;4(4):e1000046. |
| *Aspergillus spinulosporus* | NRRL 2395 | GCA_003574815.1 | Vanderbilt University | n/a | Steenwyk JL et al., Curr Biol. 2020 Jul 6;30(13):2495-2507.e7. |
| *Aspergillus niger* | CBS 513.88 | GCA_000002855.2 | DSM, The Netherlands | n/a | Pel HJ et al., Genome Nat Biotechnol. 2007 Feb;25(2):221-31. |
| *Aspergillus neoniger* | CBS 115656 | GCA_003184625.1 | DOE Joint Genome Institute | Yes | Vesth TC et al., Nat Genet, 2018 Dec;50(12):1688-1695 |
| *Aspergillus brasiliensis* | CBS 101740 | GCA_001889945.1 | DOE Joint Genome Institute | n/a | n/a |
| *Aspergillus fumigatiaffinis* | CNM-CM6805 | GCA_012656285.1 | UNICAMP | n/a | n/a |
| *Aspergillus japonicus* | CBS 114.51 | GCA_003184785.1 | DOE Joint Genome Institute | Yes | n/a |
| *Aspergillus pseudonomiae* | CBS 119388 | GCA_009193645.1 | DOE Joint Genome Institute | Yes | Kjærbølling I et al., Nat Commun, 2020 Feb 27;11(1):1106 |
| *Aspergillus flavus* | NRRL3357 | GCA_014117465.1 | University of Georgia | Yes | Kjærbølling I. et.al. Nat Commun. 2020 Feb 27;11(1):1106. |
| *Aspergillus alliaceus* | CBS 536.65 | GCA_009176365.1 | DOE Joint Genome Institute | Yes | Kjærbølling I. et.al. Nat Commun. 2020 Feb 27;11(1):1106. |
| *Aspergillus hiratsukae* | CNM-CM5793 | GCA_014281905.1 | UNICAMP | n/a | n/a |
| *Aspergillus amoenus* | S2904 | GCA_023624915.1 | Dalian Medical University | n/a | n/a |
| *Aspergillus lentulus* | IFM 47457 | GCA_010723975.1 | Medical Mycology Research Center | n/a | Takahashi H et al., Frontiers in Fungal Biology, 2021;2 |
| *Aspergillus nomiae* | NRRL 13137 | GCA_001204775.2 | USDA-ARS-SRRC | Yes | Moore GG et al., BMC Genomics. 2015 Jul 28;16(1):551. |
| *Aspergillus ochraceus* | fc-1 | GCA_004849945.1 | Institute of Agro-Products Processing Science and Technology, Chinese Academy of Agricultural Sciences (CAAS) | n/a | Wang Y et al., Appl Environ Microbiol. 2018 Sep 17;84(19):e01009-18. |
| *Aspergillus cristatus* | GZAAS20.1005 | GCA_001717485.1 | Guizhou academy of Agricultural Sciences | n/a | Ge Y et al., BMC Genomics. 2016 Jun 7;17:428. |
| *Aspergillus tubingensis* | WU-2223L | GCA_013340325.1 | Waseda University | Yes | n/a |
| *Aspergillus fischeri* | NRRL 181 | GCA_000149645.4 | TIGR | Yes | Fedorova ND et al., PLoS Genet. 2008 Apr 11;4(4):e1000046. |
| *Aspergillus tanneri* | NIH1004 | GCA_003426965.1 | JCVI | Yes | n/a |
| *Aspergillus luchuensis* | IFO 4308 | GCA_016861625.1 | Kagoshima University | n/a | Futagami T et al., Eukaryot Cell, 2011 Nov;10(11):1586-7 |
| *Aspergillus calidoustus* | SF006504 | GCA_001511075.1 | Leibniz Institute for Natural Product Research and Infection Biology e.V. Hans-Kn.ll-Institute, Germany | n/a | Horn F et al., Genome Announc, 2016 Mar 10;4(2) |
| *Aspergillus eucalypticola* | CBS 122712 | GCA_003184535.1(only) | DOE Joint Genome Institute | Yes | *Vesth TC. et. al* Nat Genet. 2018 |
| *Aspergillus bombycis* | NRRL 26010 | GCA_001792695.1(only) | USDA-ARS-SRRC | n/a | Moore GG. et.al. Genome Biol Evol. 2016 Dec 14;8(11):3297-3300. |
| *Aspergillus candidus* | CBS 102.13 | GCA_002847045.1(Only) | DOE Joint Genome Institute | n/a | n/a |
| *Aspergillus oerlinghausenensis* | CBS 139183 | GCA_014250555.1(only) | Vanderbilt University | n/a | n/a |
| *Aspergillus novoparasiticus* | CBS 126849 | GCA_009176405.1(only) | DOE Joint Genome Institute | n/a | Kjærbølling I. et.al. Nat Commun. 2020 Feb 27;11(1):1106. |
| *Aspergillus pseudotamarii* | CBS 117625 | GCA_009193445.1(only) | DOE Joint Genome Institute | n/a | Kjærbølling I. et.al. Nat Commun. 2020 Feb 27;11(1):1106. |
| *Aspergillus caelatus* | CBS 763.97 | GCA_009193585.1(only) | DOE Joint Genome Institute | n/a | Kjærbølling I. et.al. Nat Commun. 2020 Feb 27;11(1):1106. |
| *Aspergillus carbonarius* | ITEM 5010 | GCA_001990825.1(only) | DOE Joint Genome Institute | n/a | n/a |
| *Aspergillus phoenicis* | ATCC 13157 | GCA_003344505.1(only) | DOE Joint Genome Institute | n/a | n/a |
| *Aspergillus thermomutatus* | HMR AF 39 | GCA_002237265.2(only) | National Center for Biotechnology Information | n/a | n/a |
| *Aspergillus brunneoviolaceus* | CBS 621.78 | GCA_003184695.1(only) | DOE Joint Genome Institute | n/a | Vesth TC. Et.al., Nat Genet. 2018 Dec;50(12):1688-1695. |
| *Aspergillus leporis* | CBS 151.66 | GCA_009176345.1(only) | DOE Joint Genome Institute | n/a | Kjærbølling I. et.al. Nat Commun. 2020 Feb 27;11(1):1106. |
| *Aspergillus pseudoviridinutans* | IFM 55266 | GCA_018340605.1(only) | Medical Mycology Research Center | n/a | n/a |
| *Aspergillus vadensis* | CBS 113365 | GCA_003184925.1(only) | DOE Joint Genome Institute | n/a | n/a |
| *Aspergillus protuberus* | HOSP050413_4_129 | GCA_021436995.1(only) | Normandie University | n/a | n/a |
| *Aspergillus glaucus* | CBS 516.65 | GCA_001890805.1(only) | DOE Joint Genome Institute | n/a | n/a |
| *Aspergillus steynii* | IBT 23096 | GCA_002849105.1(only) | DOE Joint Genome Institute | n/a | n/a |
| *Aspergillus jensenii* | C4_18042019 | GCA_021437015.1(only) | Normandie University | n/a | n/a |
| *Aspergillus sclerotiicarbonarius* | CBS 121057 | GCA_003184635.1(only) | DOE Joint Genome Institute | n/a | n/a |
| *Aspergillus aculeatus* | ATCC 16872 | GCA_001890905.1(only) | JGI | n/a | n/a |
| *Aspergillus homomorphus* | CBS 101889 | GCA_003184865.1(only) | DOE Joint Genome Institute | n/a | Vesth TC et al., Nat Genet, 2018 Dec;50(12):1688-1695 |
| *Aspergillus quadrilineatus* | NRRL 201 | GCA_013305525.1(only) | Vanderbilt University | n/a | Steenwyk JL et al., Curr Biol, 2020 Jul 6;30(13):2495-2507.e7 |
| *Aspergillus novofumigatus* | IBT 16806 | GCA_002847465.1(only) | DOE Joint Genome Institute | n/a | n/a |
| *Aspergillus melleus* | CBS 546.65 | GCA_016097325.1(only) | National Center for Biotechnology Information | n/a | Gil-Serna J et al., Toxins (Basel), 2020 Nov 29;12(12) |
| *Aspergillus saccharolyticus* | JOP 1030-1 | GCA_003184585.1(only) | DOE Joint Genome Institute | n/a | n/a |
| *Aspergillus ibericus* | CBS 121593 | GCA_003184845.1(only) | DOE Joint Genome Institute | Yes | Vesth TC et al., Nat Genet, 2018 Dec;50(12):1688-1695 |
| Aspergillus piperis | CBS 112811 | GCA_003184755.1(only) | DOE Joint Genome Institute | Yes | Vesth TC et al., Nat Genet, 2018 Dec;50(12):1688-1695 |
| Aspergillus mulundensis | DSM 5745 | GCA_003369625.1(only) | The University of Texas Health Science Center at Houston | n/a | Wingfield BD et al., IMA Fungus, 2018 Jun;9(1):199-223 |
| Aspergillus sclerotioniger | CBS 115572 | GCA_003184525.1(only) | DOE Joint Genome Institute | n/a | Vesth TC. Et.al., Nat Genet. 2018 Dec;50(12):1688-1695. |
| Aspergillus ustus | 3.3904 | GCA_000812125.1(only) | Institute of developmentive and regenerative biology | n/a | Pi B et al., PLoS One, 2015;10(2):e0116089 |
| Aspergillus sergii | CBS 130017 | GCA_009193525.1(only) | DOE Joint Genome Institute | n/a | Kjærbølling I et al., Nat Commun, 2020 Feb 27;11(1):1106 |

**Table S2 The DNA barcodes sequences of these 77 *Aspergillus* species.**

| species | ITS | BenA | Cam |
| --- | --- | --- | --- |
| Aspergillus terreus | >NR_131276.1 Aspergillus terreus ATCC 1012 ITS region  TCCGTAGGTGAACCTGCGGAAGGATCATTACCGAGTGCGGGTCTTTATGGCCCAACCTCCCACCCGTGACTATTGTACCTTGTTGCTTCGGCGGGCCCGCCAGCGTTGCTGGCCGCCGGGGGGCGACTCGCCCCCGGGCCCGTGCCCGCCGGAGACCCCAACATGAACCCTGTTCTGAAAGCTTGCAGTCTGAGTGTGATTCTTTGCAATCAGTTAAAACTTTCAACAATGGATCTCTTGGTTCCGGCATCGATGAAGAACGCAGCGAAATGCGATAACTAATGTGAATTGCAGAATTCAGTGAATCATCGAGTCTTTGAACGCACATTGCGCCCCCTGGTATTCCGGGGGGCATGCCTGTCCGAGCGTCATTGCTGCCCTCAAGCCCGGCTTGTGTGTTGGGCCCTCGTCCCCCGGCTCCCGGGGGACGGGCCCGAAAGGCAGCGGCGGCACCGCGTCCGGTCCTCGAGCGTATGGGGCTTCGTCTTCCGCTCCGTAGGCCCGGCCGGCGCCCGCCGACGCATTTATTTGCAACTTGTTTTTTTCCAGGTTGACCTCGGATCAGGTAGGGATACCCGCTGAACTTAAGCATATCAATAAGCGGAGGAAAAGAAACCAACCGGGATTGCCTC | >KT150510.1 Aspergillus terreus  TGGATTGGATCACCTGGGGATGCTGGCTCTCGCGGAACGCAGAGTCTTATGGACATGCGTTCTCGGGCTAGGAAAGGTTCTGTGGTGGCGTGATTCTGACAACCTGTACAGGCAAACCATCTCTGGCGAGCACGGCCTTGATGGCTCCGGTGTGTAAGTGTCTCCGACGCCCGCTCAATGGGCTCCCATAATGGAGAATTACACGACAATGGACGATTCTGATGGAAGAACAGCTTCAATGGCTCCTCCGACCTCCAGCTCGAGCGCATGAACGTCTACTTCAACGAGGTACGTCCACTCCCACACCATCTTATAACAGACTCTCCACACTCCAATGACCTCGACACTAATTTCCACCCCCTATAGGCCAGCGGAAACAAGTATGTTCCTCGTGCCGTCCTCGTTGACCTTGAGCCCGGTACCATGGACGCCGTCCGTGCCGGTCCCTTCGGTCAGCTCTTCCGTCCCGACAACTTCGTCTTCGGCCAGTCTGGTGCCGGTAACAACTGG | >LN734851.1 Aspergillus terreus strain IMR-MF 724  AAGGAGCTCGTACAGTCGATCCGGCCATCACCATCCTGGTCCGCCTCGCGGATCATCTCATCCACTTCGTCGTCGGTGAGCTTCTCGCCGATGGAGGTCATCACATGGCGCAGCTCGGCGGCGGAGATGAAACCGTTGTTATCGCGGTCAAAGACCTTGAAAGCTTCCCGGATTTCCTCCTCGGAGTCGGTGTCTTTCATCTTGCGGGCCATCATCGTGAGGAACTCTGTTCAAGGAAATGTCAACAACTGGTTCCAGCGACTCGTGTAGAACGGAACGTACCAGGGAAGTCAATGGTGCCGTTGTTGTCAGCATCAACCTCGTTGATCATGTCCTGGAGCTCCGACTCGGAGGGGTTCTGGCCCAGCGAGCGCATGACGGTTCCCAGCTCCTTGGTGGTGATCTGGCCTACATGGTGTTAAATGATCGACCATAACAGAAGATAAAGTCAAGACTCGAGATTCGACATAGAACGATGCAATCGATCGGTCGCATGAGGCAATCGGAGCGGGAAATTGCACTAACCATCACCATCCTTGTCCTAAAACAGAGGAGAATCCCAGTTAGCTTGTGTTTCGGAAAACGAGCCTGGAACACGGAGAGATGGTCCATTCAGGGAAGAGGGGTAAGTCAAGACATACGAAGAGGGAGAAGGCCTCCTTGTATTCGGAGACCTGCT |
| Aspergillus eucalypticola | >KC796400.1 Aspergillus eucalypticola isolate NRRL 62632 its  AAGGATCATTACCGAGTGCGGGTCCTTTGGGCCCAACCTCCCATCCGTGTCTATTATACCCTGTTGCTTCGGCGGGCCCGCCGCTTGTCGGCCGCCGGGGGGGCGCCTTTGCCCCCCGGGCCCGTGCCCGCCGGAGACCCCAACACGAACACTGTCTGAAAGCGTGCAGTCTGAGTTGATTGAATGCAATCAGTTAAAACTTTCAACAATGGATCTCTTGGTTCCGGCATCGATGAAGAACGCAGCGAAATGCGATAACTAATGTGAATTGCAGAATTCAGTGAATCATCGAGTCTTTGAACGCACATTGCGCCCCCTGGTATTCCGGGGGGCATGCCTGTCCGAGCGTCATTGCTGCCCTCAAGCCCGGCTTGTGTGTTGGGTCGCCGTCCCCCTCTCCGGGGGGACGGGCCCGAAAGGCAGCGGCGGCACCGCGTCCGATCCTCGAGCGTATGGGGCTTTGTCACATGCTCTGTAGGATTGGCCGGCGCCTGCCGACGTTTTCCAACCATTTTTTCCAGGTTGACCTCGGATCAGGTAGGGATACCCGCTGAACTTAA | >KC796360.1 Aspergillus eucalypticola isolate NRRL 62632 beta-tubulin gene, partial cds  TGGTACGTATTCACTGCCACTGGATTGGGGATGGAACATCATCTCTCAAGCTATCTCAGCTTGAGTTCAGATGTCATCCATCGGGTATATATGTCTAACAACTCAACAGGCAGACCATCTCTGGCGAGCACGGCCTTGACGGCTCCGGTGTGTAAGTGCAACTTTTTCACACCTCTCAATTGGTCAACAATGTGGAAAGGATTGGGTATTCTGACACGCAGGATAGTTACAATGGCACCTCCGACCTCCAGCTGGAGCGCATGAACGTCTACTTCAACGAGGTTAGATCACACCGTACCTGAGTTTTTTTCACGACAATATCATCAATGTCCTGACCACTTCAGCAGGCTAGCGGTAACAAGTATGTCCCCCGTGCCGTCCTCGTCGATCTCGAGCCCGGTACCATGGACGCCGTCCGTGCCGGTCCCTTCGGCCAGCTCTTCCGTCCCGACAACTTCGTCTTCGGCCAGTCCGGTGCTGGTAACAACTGG | >KC796376.1 Aspergillus eucalypticola isolate NRRL 62632 calmodulin gene, partial cds  CAAGTTTCTGAGTACAAGGAGGCCTTCTCCCTCTTTGTGAGTGCTTCCTGAATGAACCCCCGGTCACTCATCTTCACCGGCTCATAATGCTAATGTATTTTCGTACTCAATAGGACAAGGATGGCGATGGTGGGTGGAATTCTGTCCCCTTCACGTTTTACCTGTAGCGCCCGATCCGACCGCGGGATTTCGACAGCCATTCCCCCATCAATCTGAATCATTATACTGATGTACTCTGGAAATAGGCCAGATCACCACCAAGGAGCTCGGCACTGTGATGCGCTCCCTCGGCCAGAACCCCTCCGAGTCTGAGCTTCAGGATATGATCAACGAGGTTGACGCTGACAACAACGGAACGATCGACTTCCCCGGTATGTGATAGATCTACGCCTGTAAGGCGGGAATGCCGTATGGGTTGTGATTGACTTTTGCCGCCAGAATTCCTCACCATGATGGCTCGTAAGATGAAGGACACCGACTCCGAGGAGGAAATCCGCGAGGCTTTCAAGGTCTTCGACCGCGACAACAATGGCTTCATCTCCGCCGCGGAGTTGCGCCATGTCATGACCTCCATTGGCGAGAAGCTCACCGACGACGAAGTCGATGAGATGATCCGTGAGGCTGACCAGGACGGTGATGGCCGCATCGACTGTATGTTTCCCATTCTTGATAAGCCCATGATATGACATGCTAACTCTGCTACCAGACAACGAGTTC |
| Aspergillus bombycis | >AJ874121.1 Aspergillus bombycis strain NRRL 25593 ITS CCGAGTGTAGGGTTCCTAGCGAGCCCAACCTCCCACCCGTNTTTACTGTACCTTAGTTGCTTGCGGGGGCGCCGTCAAGGCCGCCGGNCATCAGCCCCGCGGCCGCGCCCGCCGGAGACACCACGAACTCTGTCTGATCTAGTGAAGTCTGAGTTGATTGTATCGCAATCAGTTAAAACTTTCAACAATGGATCTCTTGGTTCCGGCATCGATGAAGAACGCAGCGAAATGCGATAACTAGTGTGAATTGCAGAATTCCGTGAATCATCGAGTCTTTGAACGCACATTGCGCCCCCTGGTATTCCGGGGGNCATGCCTGTCCGAGCGTCATTGCTGCCCATCAAGCACGGCTTGTGTGTTGGGTCGTCGTCCCCTCTTCGGGGGGGACGGGCCCTAAAGGCAGCGGCGGCACCGGTCCCGATCCTCGAGCGTATGGGGCTTTGTCACCCGCTCTGTAGGCCCGGCCGGCGCTTGCGAACGCAAAACAACCATTCTTTCCAGG | >EF203121.1 Aspergillus bombycis strain CBS 117817 beta-tubulin (benA)  TGGTATGTCTCGATGTCTCCGAGTCAATATGCTTTGGACCCAGGACCTCAGCAGAAACATGAGCTTGGATGTATCATATCGTGTCTGACACGTGTTTGCTAATATCTTTGCAGGCAGACCATCTCCGGCGAGCACGGCCTCGACGGCTCCGGTGTGTAAGTACAACCCGTGTACAACTCGAACGAATGACGACCAGATGGGCATTGGAAGAGTTTGAATGGGTCTGACGGGAAGGATAGTTACAATGGCTCCTCCGATCTCCAGCTGGAGCGCATGAACGTCTACTTCAACGAGGTGCGTACCTCAAAATTTTCAGCCTCTATGAAAACGCTTTGCAAGTCCTGACCGCTTCTCCAGGCCAGCGGAAACAAGTATGTCCCTCGTGCCGTCCTCGTCGATCTTGAGCCCGGTACCATGGACGCCGTCCGTGCCGGTCCCTTCGGTCAGCTGTTCCGTCCCGACAACTTCGTTTTCGGCCAGTCCGGTGC | >EF661532.1 Aspergillus bombycis isolate NRRL 25593 calmodulin gene  CCGAGTACAAGGAGGCCTTCTCTCTATTCGTAAGTCATTATCGTCGTTCGCGAAAATCGGTTGTCGTTAGCCTTTATCTGAGCACAAGCTGACTTCGCTCCTCTTGGGTCCCCAATAGGACAAGGACGGTGATGGTTAGTACAAGTCATTTTTATTCAATCTCCCTTCAAGTGCGACCAATATCTTTTAGCCGCCATAATTTTATCCATTTTCTGTTCGATCGGCTGAAGTCTTGGCGTCGATGAATTGACATGATATGCAGGCCAGATCACCACCAAGGAGTTGGGCACTGTCATGCGCTCTCTGGGCCAAAACCCCTCTGAGTCGGAACTCCAGGACATGATTAACGAGGTTGACGCCGATAACAATGGCACCATTGACTTTCCTGGTACGAGAGGGCTTCCGTACGATCCACACTTGAAATAGTTGTTAATGTTCAATCAGAGTTTCTTACGATGATGGCGAGAAAGATGAAGGATACCGACTCTGAGGAGGAGATCCGGGAGGCTTTCAAGGTTTTCGACCGCGATAACAACGGCTTCATCTCCGCTGCCGAACTGCGCCACGTCATGACCTCTATCGGCGAGAAGCTTACCGATGACGAAGTTGATGAGATGATCCGCGAGGCGGATCAGGATGGTGACGGCAGGATTGACTGTACGTTTTGAGAAGCTTTCCCGATCTAGAGCGGCTGTGAAACTGGTGATACTGACCGATTTTAGACAACGAGTTC |
| Aspergillus candidus | >MH854613.1 Aspergillus candidus strain CBS 102.13 ITS  ATCATTACCGAGTGAGGGTTTCTCTGAAGCCCAACCTCCCACCCGTGTATACCGTACCCTGTTGCTTCGGCGGGCCCGCCTCACGGCCGCCGGGGGACCTCGCGTCCCCGGGCCCGCGCCCGCCGAAGACCCCAACACGAACACTGTCTGAAAAGTGCAGTCTGAGTCGATTGTTACCAATCAGTCAAAACTTTCAACAATGGATCTCTTGGTTCCGGCATCGATGAAGAACGCAGCGAAATGCGATAACTAATGTGAATTGCAGAATTCAGTGAATCATCGAGTCTTTGAACGCACATTGCGCCCCCTGGTATTCCGGGGGGCATGCCTGTCCGAGCGTCATTGCTGCCCTCAAGCACGGCTTGTGTGTTGGGCCCCGTCCCCGGTACCCCCGGGGACGGGCCCGAAAGGCAGCGGCGGCACCGCGTCCGGTCCTCGAGCGTATGGGGCTTTGTCACCCGCTCTGCAGGCCCGGCCGGCGCCAGCCGACCAACCCAACCATTTTTTACAGGTTGACC | >MZ062547.1 Aspergillus candidus isolate BMU11063 beta-tubulin gene  TGGTACGTCTGGACAGTGTGCAGGATGGGCATGGCGGGGTTACATGGAATGAGAGACTCCGTCGTGTCACTGCCAATTAAGTCTCTCTTCGATAACATTGATTCGCTGACAACCATGCAGGCAGACCATCTCCGGCGAGCACGGCCTTGACGGCTCCGGTGTGTAAGTATTGCCGAGACATCCCATGGATACACCCTCTTCAAGGATAGAGAAGAGCGATTGGAAAAGGGGAAGTCGGTCCCTCTAACACAAATGATAGGTACAATGGCACCTCCGACCTCCAGCTGGAGCGTATGAACGTCTACTTCAACGAGGTTCGTACTCGTGATCGTCTGCTCTGAAAATCCCCTGCAGATCCCTAATTCCTTCCTTAGGCTAGCGGCAACAAGTATGTTCCCCGTGCCGTCCTCGTCGACCTGGAGCCCGGTACCATGGACGCCGTCCGTGCCGGTCCTTTCGGCCAGCTCTTCCGTCCCGACAACTTCGTCTTCGGCCAGTCCGGTGCTGGTAACAACTGG | >EU076308.1 Aspergillus candidus strain CBS 225.80 calmodulin gene  CTGCGTTCGGGGTGTTGGGAACCGGAAGATGGAACGGCGCTGATCGCAGGTCGCTTTTTCTCGTGTAACAGGATAAGGATGGCGATGGTTAGTACTCCGTTCGGACGACGCGACCGGCGTCGATCGACTCTGATGATCACGTTTCTTTGCGAACTTCTTATCTGACGTACGGCAGGACAAATCACCACCAAGGAGCTGGGCACCGTCATGCGGTCGCTGGGCCAGAACCCCTCCGAGTCGGAACTCCAGGACATGATCAACGAGGTTGACGCGGACAACAACGGCACCATCGATTTCCCTGAATTCCTCACCATGATGGCTCGTAAGATGAAGGACACCGACTCGGAGGAGGAGATCCGGGAAGCGTTCAAGGTCTTTGATCGCGACAACAACGGCTTCATCTCCGCCGCGGAGCTGCGCCACG |
| Aspergillus oerlinghausenensis | >NR_138362.1 Aspergillus oerlinghausenensis CBS 139183 ITS region; from TYPE material  CCGAGTGAGGGCCCTCTGGGTCCAACCTCCCACCCGTGTCTATCGTACCTTGTTGCTTCGGCGGGCCCGCCGTTTCGACGGCCGCCGGGGAGGCCTCGCGCCCCCGGGCCCGCGCCCGCCGAAGACCCCAACATGAACGCTGTTCTGAAAGTATGCAGTCTGAGTTGATTATCATAATCAGTTAAAACTTTCAACAACGGATCTCTTGGTTCCGGCATCGATGAAGAACGCAGCGAAATGCGATAAGTAATGTGAATTGCAGAATTCAGTGAATCATCGAGTCTTTGAACGCACATTGCGCCCCCTGGTATTCCGGGGGGCATGCCTGTCCGAGCGTCATTGCTGCCCTCAAGCACGGCTTGTGTGTTGGGCCCCCGTCCCCCTCTCCCGGGGGACGGGCCCGAAAGGCAGCGGCGGCACCGCGTCCGGTCCTCGAGCGTATGGGGCTTTGTCACCCGCTCTGTAGGCCCGGCCGGCGCCAGCCGACACCCAACTTTATTTCTAA | >KT359603.1 Aspergillus oerlinghausenensis strain CBS 139183 beta-tubulin (BenA) gene  GCTTTCTGGTATGTCTCGACCTCAATGCTTGGATGACGGGTGATTGGGACCTCTCATCTTAGCAGGCTGCCCTCCGTGGGTTCAGCCTCGCTGTCATGGGTATCAGCTAACAAATCTACAGGCAGACCATCTCTGGTGAGCACGGCCTTGACGGCTCTGGCCAGTAAGTTCGACCTATATCCTCCCAATTGAGAAAGCGGCGGAAACACGAAAGGCAAGGAAGAAGAGGACGCGTGTCTGATGGGAATAATAGCTACAATGGCTCCTCCGATCTCCAGCTGGAGCGTATGAACGTCTACTTCAACGAGGTGCGTGGATGAAACCCTCGACTCTATACTATTTCGGCAACATCTCACGATCTGACTCGCTACTAGGCCAACGGTGACAAGTATGTTCCTCGTGCCGTTCTGGTCGATCTCGAGCCTGGTACCATGGACGCTGTCCGTGCTGGTCCCTTCGGCGAGCTCTTCCGTCCCGACAACTTCGTCTTCGGCCAGTCTGGTGCTGGTAACAACTGGGCCAA | >KT359605.1 Aspergillus oerlinghausenensis strain CBS 139183 calmodulin (CaM) gene  TTCTCTCTCTTCGTAAGTGAACTGTCCAAGTCCCTAGTCGTTGCATAGGAGGGATCTCCACAATATTGAGGGTGTGCGCTCACACGAGATTCGACGTATAGGACAAGGATGGTGATGGTTAGTGACCCTTTTTCCGCTCCTCGAACTTCGGCTTCCATGCGATCATGTTCAAACGCCGACTTACAATACCCGGAAATGACCCGTCAGTACTGATAATATCTATGTTTGACTATTAGGCCAGATCACCACCAAGGAATTGGGCACTGTGATGCGCTCCCTGGGCCAGAACCCTTCCGAGTCAGAGCTGCAAGATATGATCAACGAGGTGGATGCTGACAACAACGGCACCATCGATTTCCCTGGTATGTGATGCATTTGGTGTGACCTCGGGAGGGAGGAGAACAATCATTAACTCGTATTCAGAATTCCTTACTATGATGGCTCGGAAGATGAAGGACACCGACTCCGAAGAGGAAATCCGGGAAGCTTTCAAGGTCTTCGACCGTGACAACAACGGTTTCATCTCCGCTGCGGAGCTGCGCCA |
| Aspergillus oryzae |  | >MT410160.1 Aspergillus oryzae isolate CCMFBH-925 beta-tubulin (benA) gene, partial cds  TGGTAACCAAAACGGTGCTGCTTTCTGGTATGTCTCAATGCCTTCGAGTTAGTATGCTTTGGACCAAGGAACTCCTCAAAAGCATGATCTCGGATGTGTCCTGTTATATCTGCCACATGTTTGCTAACAACTTTGCAGGCAAACCATCTCTGGCGAGCACGGCCTTGACGGCTCCGGTGTGTAAGTACAGCCTGTATACACCTCGAACGAACGACGACCATATGGCATTAGAAGTTGGAATGGATCTGACGGCAAGGATAGTTACAATGGCTCCTCCGATCTCCAGCTGGAGCGTATGAACGTCTACTTCAACGAGGTGCGTACCTCAAAATTTCAGCATCTATGAAAACGCTTTGCAACTCCTGACCGCTTCTCCAGGCCAGCGGAAACAAGTATGTCCCTCGTGCCGTCCTCGTTGATCTTGAGCCTGGTACCATGGACGCCGTCCGTGCCGGTCCCTTCGGTCAGCTCTTCCGTCCCGACAACTTCGTTTTCGGCCAGTCCGGTGCTGGTAACAACTGGGCCAAGGGTC | >MT410160.1 Aspergillus oryzae isolate CCMFBH-925 beta-tubulin (benA) gene, partial cds  TGGTAACCAAAACGGTGCTGCTTTCTGGTATGTCTCAATGCCTTCGAGTTAGTATGCTTTGGACCAAGGAACTCCTCAAAAGCATGATCTCGGATGTGTCCTGTTATATCTGCCACATGTTTGCTAACAACTTTGCAGGCAAACCATCTCTGGCGAGCACGGCCTTGACGGCTCCGGTGTGTAAGTACAGCCTGTATACACCTCGAACGAACGACGACCATATGGCATTAGAAGTTGGAATGGATCTGACGGCAAGGATAGTTACAATGGCTCCTCCGATCTCCAGCTGGAGCGTATGAACGTCTACTTCAACGAGGTGCGTACCTCAAAATTTCAGCATCTATGAAAACGCTTTGCAACTCCTGACCGCTTCTCCAGGCCAGCGGAAACAAGTATGTCCCTCGTGCCGTCCTCGTTGATCTTGAGCCTGGTACCATGGACGCCGTCCGTGCCGGTCCCTTCGGTCAGCTCTTCCGTCCCGACAACTTCGTTTTCGGCCAGTCCGGTGCTGGTAACAACTGGGCCAAGGGTC |
| Aspergillus versicolor |  | >MT410167.1 Aspergillus versicolor isolate CCMFBH-950 beta-tubulin (benA)  CCAAATTGGTGCTGCTTTCTGGTGCGTCGAAAATTTCATCCATTTCAGATGGTATTTCCTTTGGTGCTTTTTGCTAACGACCCTATAGGCAGACCATCTCCGGCGAGCACGGCCTTGATGGCTCCGGTGTGTGAGTACAACCCGTCCAAGACTCGATCAAAACACGACCGAACACAACCCCTGATATAATCCAGTTACAATGGTACCTCCGACCTCCAGCTCGAGCGTATGAACGTCTACTTCAACGAGGCCAGCGGCAACAAGTACGTTCCCCGTGCCGTTCTCGTCGATCTCGAGCCCGGTACTATGGACGCTGTCCGTGCCGGTCCCTTCGGTCAGCTCTTCCGTCCCGACAACTTCGTCTTTGGCCAGTCCGGTGCTGGTAACAACTGGGCCAAGGGTCA | >MT410167.1 Aspergillus versicolor isolate CCMFBH-950 beta-tubulin (benA) gene, partial cds  CCAAATTGGTGCTGCTTTCTGGTGCGTCGAAAATTTCATCCATTTCAGATGGTATTTCCTTTGGTGCTTTTTGCTAACGACCCTATAGGCAGACCATCTCCGGCGAGCACGGCCTTGATGGCTCCGGTGTGTGAGTACAACCCGTCCAAGACTCGATCAAAACACGACCGAACACAACCCCTGATATAATCCAGTTACAATGGTACCTCCGACCTCCAGCTCGAGCGTATGAACGTCTACTTCAACGAGGCCAGCGGCAACAAGTACGTTCCCCGTGCCGTTCTCGTCGATCTCGAGCCCGGTACTATGGACGCTGTCCGTGCCGGTCCCTTCGGTCAGCTCTTCCGTCCCGACAACTTCGTCTTTGGCCAGTCCGGTGCTGGTAACAACTGGGCCAAGGGTCA |
|  |  | >MT410139.1 Aspergillus wentii isolate CCMFBH-873 beta-tubulin (benA) gene  TCGGTGCTGCTTTCTGGTATGTCCCACTGATGATATAATGGAGGGGACAAAAGAATTGGATTCCTCCACTTTTAAAGGCAATTTGCTAACAGGTTCATAGGCAAACCATCTCTGGTGAGCACGGCCTTGACGGTGCTGGTGTGTGAGTACATCCTGTATTTTGGCGGTGGAGGAAAAAAATATGATCAAATGTCTGATGAAACGACAGTTACAATGGCACCTCCGACCTCCAGTTGGAGCGTATGAACGTCTACTTCAACGAGGTCCGTTATTCGAAATCCTTCCGATGGATATAGCGGTATGCTGACAACCTACCCAGGCTAGCGGACACAAGTATGTTCCCCGTGCCGTTCTGGTCGACCTTGAGCCCGGTACCATGGACGCTGTCCGTTCCGGTCCTTTCGGCCAGCTCTTCCGCCCCGACAACTTCGTCTTCGGCCAGTCTGGTGCTGGTAACAACTGG |  |
| Aspergillus novoparasiticus | >NR_159637.1 Aspergillus novoparasiticus CBS 126849 ITS region  ATTACCGAGTGTAGGGTTCCTAGCGAGCCCAACCTCCCACCCGTGTTTACTTGTACCTTAGTTGCTTCGGCGGGCCCGCCGTCATGGCCGCCGGGGGCGTTCAGCCCCGGGCCCGCGCCCGCCGGAGACACCACGAACTCTGTCTGATCTAGTGAAGTCTGAGTTGATTGTATCGCAATCAGTTAAAACTTTCAACAATGGATCTCTTGGTTCCGGCATCGATGAAGAACGCAGCGAAATGCGATAACTAGTGTGAATTGCAGAATTCCGTGAATCATCGAGTCTTTGAACGCACATTGCGCCCCCTGGTATTCCGGGGGGCATGCCTGTCCGAGCGTCATTGCTGCCCATCAAGCACGGCTTGTGTGTTGGGTCGTCGTCCCCTCTCCGGGGGGGACGGGCCCCAAAGGCAGCGGCGGCACCGCGTCCGATCCTCGAGCGTATGGGGCTTTGTCACCCGCTCTGTAGGCCCGGCCGGCGCTTGCCGAACGCAAAACAATCTTTCCA | >MG517684.1 Aspergillus novoparasiticus strain DTO 223-C3 beta-tubulin (BenA) gene  TGGTATGTCTCGATACCTTCGAGTTAATATGCTTTGGACCAAGGAACTCCTCAAAAGCATGATCTCGGATGTGTCCTATTATATCTGCCACGTGTTTGCTAACAACTTTGCAGGCAAACCATCTCTGGCGAGCACGGCCTTGACGGCTCCGGTGTGTAAGTACAGCCTGTATACACCTCGAACGAACGACGACCATATGGCATTAGAAGTTGGAATGGATCTGACGGCAAGGATAGTTACAATGGCTCCTCTGATCTCCAGCTGGAGCGTATGAACGTCTACTTCAACGAGGTGCGTACCTCAAAATTTTCAGCATCTATGAAAACGCTTTGCAACTCCTGACCGCTTCTCCAGGCCAGCGGAAACAAGTATGTCCCTCGTGCCGTCCTCGTCGATCTTGAGCCTGGTACCATGGACGCCGTCCGTGCCGGTCCCTTCGGTCAGCTCTTCCGTCCCGACAACTTCGTTTTCGGCCAGTCCGGTGCTGGTAACAACTGGGCCAAG | >MG518055.1 Aspergillus novoparasiticus strain DTO 223-C3 calmodulin (CaM) gene,  TCGTAAGTAGTTATCGTCGTTCGCGAAAATTGGTTTTTGTTAGTCGTCTTGATCTGAACACAAGCTGACTTGGCTTTTCTTGGGTTTCCTATAGGACAAGGACGGTGATGGTTAGTACAGTTTATTTTATTCATTCTCCCTTCAAATGCGACCAAGATGTTTTAGCCGCCATAAGTTTATCAAGTTTCTGTTCGATCGGCTGAAGTCTTGGCATTGATGAATTGACTTGATATGCAGGCCAGATCACCACCAAGGAGTTGGGCACTGTCATGCGCTCTCTGGGTCAAAACCCCTCTGAGTCGGAACTCCAGGACATGATTAACGAGGTTGACGCCGACAACAATGGCACCATTGACTTCCCTGGTACGAGACGGCTTCCGTACGATTCATAAATGAAATAGTTGTTAATCGTCCAAATAGAATTCCTTACGATGATGGCGAGAAAGATGAAGGATACCGACTCTGAGGAGGAGATCCGGGAGGCTTTCAAGGTTTTCGACCGCGATAACAACGGCTTCATCTCCGCTGCCGAA |
| Aspergillus awamori | >NR_077143.1 Aspergillus awamori ITS region  AAAGTCGTAACAAGGTTTCCGTAGGTGAACCTGCGGAAGGATCATTACCGAGTGCGGGTCCTTTGGGCCCAACCTCCCATCCGTGTCTATTGTACCCTGTTGCTTCGGCGGGCCCGCCGCTTGTCGGCCGCCGGGGGGGCGCCTCTGCCCCCCGGGCCCGTGCCCGCCGGAGACCCCAACACGAACACTGTCTGAAAGCGTGCAGTCTGAGTTGATTGAATGCAATCAGTTAAAACTTTCAACAATGGATCTCTTGGTTCCGGCATCGATGAAGAACGCAGCGAAATGCGATAACTAATGTGAATTGCAGAATTCAGTGAATCATCGAGTCTTTGAACGCACATTGCGCCCCCTGGTATTCCGGGGGGCATGCCTGTCCGAGCGTCATTGCTGCCCTCAAGCCCGGCTTGTGTGTTGGGTCGCCGTCCCCCTCTCCGGGGGGACGGGCCCGAAAGGCAGCGGCGGCACCGCGTCCGATCCTCGAGCGTATGGGGCTTTGTCACATGCTCTGTAGGATTGGCCGGCGCCTGCCGACGTTTTCCAACCATTCTTTCCAGGTTGACCTCGGATCAGGTAGGGATACCCGCTGAACTTAAGCATATCAATAA | >MH781284.1 Aspergillus awamori strain cmaf108 beta-tubulin (benA)  TGCTGCTTTCTGGTACGTATACAACTGCCATTGGATTGGGGATGGAACATCGTCTCTTAGGCTATCTCAGCTTGAGTTCAGATGTTGTCCATTAGGTACATGCTATCGGTCTAAGAACACGTCTAACAATTCAACAGGCAGACCATCTCTGGCGAGCACGGCCTTGACGGCTCCGGTGTGTAAGTGCAACTTTTTCACACCTCTCAATTGGTCAACAATGGGCAAAGGGTTGGGTCTTCTGACACGCAGGATAGTTACAATGGCACCTCCGACCTCCAGCTGGAGCGCATGAACGTCTACTTCAACGAGGTGAGATCCATCGGACCTTTGCTTTTACACGACAATATCATCAATGTCCTAATCACTTCAGCAGGCTAGCGGTAACAAGTATGTTCCTCGTGCCGTCCTCGTCGACCTCGAGCCCGGTACCATGGACGCCGTCCGTGCCGGTCCTTTCGGCCAGCTCTTCCGCCCCGACAACTTCGTCTTCGGCCAGTCCGGTGCTGGTAACAACTGGGCCAAG | >LN890517.1 Aspergillus awamori CaM gene for Calmodulin, strain FCBP1525  CCCCTGAATGAACCCCCGATCACTCAAATTGATGTCCTATCTTAACCGGCTCATAATGCTAATGTATTTTCAAACTCAATAGGACAAGGATGGCGATGGTGGGTGGAATTCTGTCCCCTTCACGTTTTACCTGTAGCGCCCGATCCGACCGCGGGATTTCGACAGCTATTTCCCCCTTCGATCTGAATCATAATACTGATGTAATCTGGAAATAGGCCAGATCACCACCAAGGAGCTCGGCACTGTGATGCGCTCCCTCGGCCAGAACCCCTCCGAGTCTGAGCTTCAGGACATGATCAACGAGGTTGACGCTGACAACAACGGAACGATCGACTTCCCCGGTATGTGATAGATCTACGCCTGTAAGGCGGGAATGCCGTATGGATTGTGATTGACTTTTGCCGCCAGAATTCCTTACCATGATGGCTCGTAAGATGAAGGACACCGACTCCGAGGAGGAAATCCGCGAGGCTTTCAAGGTCTTCGACCGCGACAACAATGGTTTCATCTCCGCCGCGGAGTTGCGCCACGT |
| Aspergillus nidulans | > Aspergillus nidulans ATCC 10074 ITS  TCCGTAGGTGAACCTGCGGAAGGATCATTACCGAGTGCGGGCTGCCTCCGGGCGCCCAACCTCCCACCCGTGACTACCTAACACTGTTGCTTCGGCGGGGAGCCCCCCAGGGGCGAGCCGCCGGGGACCACTGAACTTCATGCCTGAGAGTGATGCAGTCTGAGCCTGAATACAAATCAGTCAAAACTTTCAACAATGGATCTCTTGGTTCCGGCATCGATGAAGAACGCAGCGAACTGCGATAAGTAATGTGAATTGCAGAATTCAGTGAATCATCGAGTCTTTGAACGCACATTGCGCCCCCTGGCATTCCGGGGGGCATGCCTGTCCGAGCGTCATTGCTGCCCTCAAGCCCGGCTTGTGTGTTGGGTCGTCGTCCCCCCCGGGGGACGGGCCCGAAAGGCAGCGGCGGCACCGTGTCCGGTCCTCGAGCGTATGGGGCTTTGTCACCCGCTCGATTAGGGCCGGCCGGGCGCCAGCCGGCGTCTCCAACCTTATTTTTCTCAGGTTGACCTCGGATCAGGTAGGGATACCCGCTGAACTTAAG | >MH781291.1 Aspergillus nidulans strain cmaf113 beta-tubulin (benA)  TGCTGCTTTCTGGTGAGCGAAAATTTCACCTTTTTGGAATGATTTCTTTCTTCGTACACCTGCTAACAAGCCAACAGGCAGACCATCTCCGGTGAGCACGGCCTCGATGGCTCCGGTGTGTGAGTACAATCCGTCCAGCATCCGCTGAAAACACGACACCTCAACAGAACCTGACACAACCCAGTTACAATGGTACCTCCGACCTTCAACTCGAGCGTATGAACGTCTACTTCAATGAGGTCAGTGGATGAGATATCAGGTAGCAGTTTGGTGTGTCTGACTTCGTCCAGGCCAGCGGTAACAAGTACGTTCCCCGTGCCGTCCTCGTCGATCTCGAGCCCGGTACTATGGATGCCGTCCGCGCCGGTCCCTTCGGCGAGCTCTTCCGTCCCGACAACTTCGTTTTCGGCCAGTCCGGTGCTGGTAACAACTGGGCCAAG | > Aspergillus nidulans NRRL 187 CaM gene for calmodulin,  AGAGCAAGTTTCCGAGTACAAGGAGGCCTTCTCCCTATTTGTAAGTGCCATTGGTTACTGTTATATCAAAATCGAATTTGTATTGAGAGTATACTAATACATTCCGCACTAAACAGGACAAGGATGGCGATGGTTAGTGCATCTGTCCCCCCAGGCTTGATCGCATTCGCCCAGCATGTCTGCTGTAGCTCTATATAACAGTTTCTGACAAACGGCGACAGGCCAGATTACCACTAAGGAGCTTGGCACTGTCATGCGCTCGCTCGGTCAGAATCCTTCAGAGTCTGAGCTTCAGGACATGATCAACGAAGTTGACGCCGACAACAATGGCACCATTGACTTTCCAGGTACGCGAACTCCCCAATCTACTTCGCACCAGCCTAGAAATGTACTAATGCTAAACAGAGTTCCTTACCATGATGGCCAGAAAGATGAAGGACACCGATTCCGAGGAGGAAATTCGGGAGGCGTTCAAGGTCTTCGACCGTGACAACAATGGTTTCATCTCCGCTGCTGAGCTGCGTCACGTCATGACCTCGATCGGTGAGAAGCTCACCGATGACGAAGTCGACGAGATGATCCGCGAGGCGGACCAGGATGGCGACGGCCGAATTGACTGTACGTTGGCTCCCCGCTTATCCTTGACCGTAGAAGAGGTATGATACTGATCGGCTGCAGACAACGAATTC |
| Aspergillus heteromorphus | > Aspergillus heteromorphus CBS 117.55 ITS regionCCGAGTGCGGGTCCTTCGGGGCCCAACCTCCCACCCGTGTCTACCGTACCATGTTGCTTCGGCGGGCCCGCCGCCTGTCGGCCGCCGGGGGGGCTCTGCCCCCCGGGCCCGTGCCCGCCGAAGACCCCAACACGAACACTGTCTGAAAGCATGCAGTCTGAGTTGATTCTTTGCAATCAGTTAAAACTTTCAACAATGGATCTCTTGGTTCCGGCATCGATGAAGAACGCAGCGAAATGCGATAACTAATGTGAATTGCAGAATTCAGTGAATCATCGAGTCTTTGAACGCACATTGCGCCCCCTGGTATTCCGGGGGGCATGCCTGTCCGAGCGTCATTGCTGCCCTCAAGCCCGGCTTGTGTGTTGGGTCGTCGTCCCCCCCCCGGGGGGGACGGGCCCGAAAGGCAGCGGCGGCACCGCGTCCGATCCTCGAGCGTATGGGGCTTTGTCACCCGCTCCGTAGGACCGGCCGGCGCCTGCCGACCTCTCCAACCATTTTCCA | >MT410181.1 Aspergillus heteromorphus isolate CCMFBH-999 beta-tubulin (benA) gene  GTGCTGCTTTCTGGTATGTCAGCATGAGTGCATTGCACTTTCCACGGGGGTCCCATGAAGCTTTGGGAGCATAAATTCATGGTGTCTCGCGTTTGTTTTGGCTTGCGGATTATCTGACAACTCAACAGGCAGACCATCTCTGGTGAGCACGGCCTCGACGGCTCCGGTGTGTAAGTGCAACCCGCCTACGTCTATCGATCAACAACAAGAAAGGATGGGTTTCCTGACAAGGAAACAGTTACAATGGCTCCTCCGACCTCCAGCTGGAGCGCATGAACGTCTACTTCAACGAGGTGCGTGTCTGCCATTCGTGTTTTCCGCCATGTCTCAATTTCTGACCCCTTCATCAGGCTAGCGGCAACAAGTATGTTCCCCGTGCCGTCCTCGTCGACCTTGAGCCCGGTACCATGGACGCCGTCCGTGCCGGTCCTTTCGGCCAGCTCTTCCGTCCCGACAACTTCGTCTTCGGTCAGTCCGGTGCCGGTAACAACTGG | >MH645031.1 Aspergillus heteromorphus voucher IHEM 5801 calmodulin (caM) gene  CAAGTTTCCGAGTACAAGGAGGCCTTCTCCCTTTTTGTGAGTAAATTCAGAAACCCCTTACTGGAACACAGGCTAACGAGCCCTTTTGCGTAAATAGGACAAGGATGGCGATGGTGGGTTCCCGCTCCTCACCCCTCAACGTTCGGCGTTGTCAACCGCCCCGACCGCTGGGCCTCGAAGGCGATAATTCGATGAAATAGACCCATAATGCTGATTCTATTCGGTGTACAGGCCAGATCACCACCAAGGAGCTGGGCACTGTGATGCGCTCTCTCGGCCAGAACCCCTCCGAGTCTGAGTTGCAGGACATGATCAACGAGGTCGACGCTGACAACAACGGCACCATCGATTTCCCTGGTATGTGATCGGCTCCATCCCACGAGGGGCAGGAATGCGGACTAGATGTTGACTTTGGCTAGAATTCCTTACCATGATGGCCCGTAAGATGAAGGACACCGATTCCGAGGAGGAGATCCGTGAGGCGTTCAAGGTCTTCGACCGCGACAACAACGGTTTCATCTCCGCCGCGGAGCTGCGCCACGTCATGACCTCCATCGGCGAGAAGCTCACCGATGATGAGGTTGATGAGATGATCCGCGAGGCGGACCAGGACGGTGACGGCCGTATTGACTGTTTGTTTCCCACCCATGCCCCGCTCTGCTTGACATACTAATCGCCTCCCAGACAATGAGTTC |
| Aspergillus fumigatus | > Aspergillus fumigatus ATCC 1022 ITS region  AACGACTCCCCAGAGCCGGAAAGTTGGTCAAACCCGGTCATTTAGAGGAAGTAAAAGTCGTAACAAGGTTTCCGTAGGTGAACCTGCGGAAGGATCATTACCGAGTGAGGGCCCTCTGGGTCCAACCTCCCACCCGTGTCTATCGTACCTTGTTGCTTCGGCGGGCCCGCCGTTTCGACGGCCGCCGGGGAGGCCTTGCGCCCCCGGGCCCGCGCCCGCCGAAGACCCCAACATGAACGCTGTTCTGAAAGTATGCAGTCTGAGTTGATTATCGTAATCAGTTAAAACTTTCAACAACGGATCTCTTGGTTCCGGCATCGATGAAGAACGCAGCGAAATGCGATAAGTAATGTGAATTGCAGAATTCAGTGAATCATCGAGTCTTTGAACGCACATTGCGCCCCCTGGTATTCCGGGGGGCATGCCTGTCCGAGCGTCATTGCTGCCCTCAAGCACGGCTTGTGTGTTGGGCCCCCGTCCCCCTCTCCCGGGGGACGGGCCCGAAAGGCAGCGGCGGCACCGCGTCCGGTCCTCGAGCGTATGGGGCTTTGTCACCTGCTCTGTAGGCCCGGCCGGCGCCAGCCGACACCCAACTTTATTTTTCTAAGGTTGACCTCGGATCAGGTAGGGATACCCGCTGAACTTAAGCATATCAATAAGCGGAGGAAAAGAAACCAACAGGGATTGCCTCAGTAACGGCGAGTGAA | > Aspergillus fumigatus strain NRRL 62428 BenA (BenA) gene  ATGTTGTGCCTAATACCCCCCCCTGTCCCCTTTAGGAGACGCGTCGATTCGGGCTCTCCCTGGATAGAAGAACCCCACTTGTTCCTCAAGAATGCAGCTTAGCTAACTCGTTTTTTTTTATTCTACAGGTTCACCTTCAGACCGGTCAGTGTGTAAGTACTGCCTGCCGTGGGTGAAGAAGCATAGGGATGGTGTTTGCATTGAGCAGAAGCTAAACTTGATTCTTGGTGACAGGGTAACCAAATTGGTGCCGCTTTCTGGTATGTCTTGACCTCAAAGCTTGGATGACGGGTGATTGGGATCTCTCATCTTAGCAGGCTACCTCCATGGGTTCAGCCTCACTGTCATGGGTATCAGCTAACAAATCTACAGGCAGACCATCTCTGGTGAGCATGGCCTTGACGGCTCTGGCCAGTAAGTTCGACCTATATCCTCCCAATTGAGAAAGCGGCGGAAACACGGAAAACAAGGAAGAAGCGGACGCGTGTCTGATGGGAAATAATAGCTACAATGGCTCCTCCGATCTCCAGCTGGAGCGTATGAACGTCTATTTCAACGAGGTGTGTGGATGAAACTCTTGATTTATACTATTTCGGCAACATCTCACGATCTGACTCGCTACTAGGCCAACGGTGACAAATATGTTCCTCGTGCCGTTCTGGTCGATCTCGAGCCTGGTACCATGGACGCTGTCCGTGCCGGTCCCTTCGGCGAGCTATTCCGTCCCGACAACTTCGTCTTCGGCCAGTCCGGTGCTGGTAACAACTGG | > Aspergillus fumigatus strain NRRL 62427 CaM (CaM)  AGGTTTCCGAGTACAAGGAAGCTTTCTCTCTCTTCGTAAGTGAACTGTCCAAGTCCCTGGTCGTTGTATAGGAGGGATCTCCAGAATATTGAGGGTGTGCGCTGACACGAGATTTGACGTATAGGACAAGGATGGTGATGGTTAGTGACCCTTTTTCCACTCCTCGAACTTCGGCTTCCATGCGATCATGTTCAAACGCCGACTCACAAT  ATCCGGAAATGACCCGTCAGTACTGATAATATCTATGTTTGACTATCAGGCCAGATCACCACCAAGGAATTGGGCACTGTAATGCGCTCTCTGGGCCAGAACCCTTCCGAGTCAGAGCTGCAAGATATGATCAACGAGGTGGATGCTGACAACAACGGCACCATCGATTTCCCCGGTATGTGATACTTTCGGTATGAACTCGGGAGGGGAGAGAACAATCATTAACTTGTAATCAGAATTCCTTACCATGATGGCTCGGAAGATGAAGGACACCGACTCCGAAGAGGAAATTCGGGAAGCTTTCAAGGTCTTCGACCGCGACAACAACGGTTTCATCTCCGCTGCGGAGCTGCGCCACGTTATGACCTCTATCGGGGAGAAGCTCACTGACGACGAAGTTGACGAGATGATTCGCGAGGCGGATCAGGACGGTGACGGCCGGATTGATTGTACGTTCATCAAACAGCTTTCTTCTGTTGCACCTCGATCTTCATGGGACATTTCGCTGATTGTTTCTAGACAAC |
| Aspergillus parasiticus | > Aspergillus parasiticus var. globosus CBS 260.67 ITS region  TCCTAGCGAGCCCAACCTCCCACCCGTGTTTACTGTACCTTAGTTGCTTCGGCGGGGCCCGCCGTCATGGCCGCCGGGGGCGTCAGCCCCGGGCCCGCGCCCGCCGGAGACACCACGAACTCTGTCTGATCTAGTGAAGTCTGAGTTGATTGTATCGCAATCAGTTAAAACTTTCAACAATGGATCTCTTGGTTCCGGCATCGATGAAGAACGCAGCGAAATGCGATAACTAGTGTGAATTGCAGAATTCCGTGAATCATCGAGTCTTTGAACGCACATTGCGCCCCCTGGTATTCCGGGGGGCATGCCTGTCCGAGCGTCATTGCTGCCCATCAAGCACGGCTTGTGTGTTGGGTCGTCGTCCCCTCTCCGGGGGGGACGGGCCCCAAAGGCAGCGGCGGCACCGCGTCCGATCCTCGAGCGTATGGGGCTTTGTCACCCGCTCTGTAGGCCCGGCCGGCGCTTGCCGAACGCAAAACAACCATTTTTTCCA | > Aspergillus parasiticus gene for beta tubulin, strain FMR 15447  GCTTTCTGGTATGTCTCGATACCTTCGAGTTAATATGCTTTGGACCAAGGAACTCCTCAAAAGCATGATCTCGGATGTGTCCTATTATATCTGCCACGTGTTTGCTAACAACTTTGCAGGCAAACCATCTCTGGCGAGCACGGCCTTGACGGCTCCGGTGTGTAAGTACAGCCTGTATACACCTCGAACGAACGACGACCATATGGCATTAGAAGTTGGAATGGATCTGACGGCAAGGATAGTTACAATGGCTCCTCTGATCTCCAGCTGGAGCGTATGAACGTCTACTTCAACGAGGTGCGTACCTCAAAATTTTCAGCATCTATGAAAACGCTTTGCAACTCCTGACCGCTTCTCCAGGCCAGCGGAAACAAGTATGTCCCTCGTGCCGTCCTCGTCGATCTTGAGCCTGGTACCATGGACGCCGTCCGTGCCGGTCCCTTCGGTCAGCTCTTCCGTCCCGACAACTTCGTTTTCGGCCAGTCCGGTGCTGGTAACAACTGGGCCAAG | >MG518129.1 Aspergillus parasiticus strain DTO 303-C2 calmodulin (CaM) gene, partial cds  TCGTAAGTAGTTATCGTCGTTCGCGAAAATTGGTTTTTGTTAGTCGTCTTGATTTGAACACAAGCTGACTTGGCTTTTCTTGGGTTTCCTATAGGACAAGGACGGTGATGGTTAGTACAGTTTATTTTATTCATTCTCCCTTCAAATGCGACCAATATGTTTTGGCCGCCATAAGTTTATCAAGTTTCTGTTCGATCGGCTGAAGTCTTGGCATTGATGAATTGACTTGATATGCAGGCCAGATCACCACCAAGGAGTTGGGCACTGTCATGCGCTCTCTGGGTCAAAACCCCTCTGAGTCGGAACTCCAGGACATGATTAACGAGGTTGACGCCGACAACAATGGCACCATTGACTTCCCTGGTACGAGACGGCTTCGTACGATTCATAAATGAAATAGTTGTTAATCGTCCAAATAGAATTCCTTACGATGATGGCGAGAAAGATGAAGGATACCGACTCTGAGGAGGAGATCCGGGAGGCTTTCAAGGTTTTCGACCGCGATAACAACGGCTTCATCTCCGCTGCCGAA |
| Aspergillus aculeatinus | > Aspergillus aculeatinus CBS 121060 ITS  TCGTAACAAGGTTTCCGTAGGTGAACCTGCGGAAGGATCATTACCGAGTGCTGGGTCCTTCGGGGCCCAACCTCCCACCCGTGCTTACCGTACCCTGTTGCTTCGGCGGGCCCGCCTTCGGGCGGCCCGGGGCCTGCCCCCGGGACCGCGCCCGCCGGAGACCCCAATGGAACACTGTCTGAAAGCGTGCAGTCTGAGTCGATTGATACCAATCAGTCAAAACTTTCAACAATGGATCTCTTGGTTCCGGCATCGATGAAGAACGCAGCGAAATGCGATAACTAATGTGAATTGCAGAATTCAGTGAATCATCGAGTCTTTGAACGCACATTGCGCCCCCTGGTATTCCGGGGGGCATGCCTGTCCGAGCGTCATTTCTCCCCTCCAGCCCCGCTGGTTGTTGGGCCGCGCCCCCCCGGGGGCGGGCCTCGAGAGAAACGGCGGCACCGTCCGGTCCTCGAGCGTATGGGGCTCTGTCACCCGCTCTATG  GGCCCGGCCGGGGCTTGCCTCGACCCCCAATCTTCTCAGATGACCTC | >EU159227.1 Aspergillus aculeatinus strain CBS 121871 beta-tubulin (benA) gene, partial cds  CATCTCTGGTGAGCACGGCCTCGATGGCGCCGGTGTGTAAGTGCAGACTTCCCGGGCGGCAGGACCAGCGGGTCGCGGAAAATGGTGAAGGATATTAATGGAAAGGACAGTTACAATGGCTCCTCCGACCTCCAGCTGGAGCGCATGAACGTCTACTTTAACGAGGTTCGTCTTCGAGTCCTTCCTCCGGCTTTCCCGCATCAGCTCCTGACTGCTTCACCAGGCTAGCGGCAACAAGTATGTTCCCCGTGCCGTCCTCGTCGACCTCGAGCCCGGTACCATGGACGCCGTCCGCGCCGGTCCTTTCGGCCAGCTTTTCCGCCCCGACAACTTCGTCTTCGGTCAATCCGGTGCTGGTAACAACTGG | >HE578095.2 Aspergillus aculeatinus partial caM gene for calmodulin, exons 1-5, isolate F-596  TGACTGAGGAGCAAGTTTCCGAGTACAAGGAGGCCTTCTCCCTCTTTGTAAGCGACCCCGATGCTTTCTCGTCTGCTATGAAACAATGTTTACTGATTCGTTTCTCGTGATCCCTGATAGGACAAGGATGGCGATGGTTAGTGTAGTTCCTTACCCCTCAACGAAACCCGCCTGGCACCACTATAACCACCACACCGCAAACGCCACAGAAACTATCAAGAATTAATCGCATTTCTGACACAACCTATGCTACAGGACAAATTACCACCAAGGAGTTGGGTACCGTTATGCGCTCTCTCGGCCAGAACCCCTCCGAGTCTGAGCTCCAGGACATGATCAACGAGGTTGACGCTGACAACAACGGCACTATCGACTTCCCCGGTATGTGATGGCTATGCATCGGGTTCCGGACATAGTATTGACAGCTGCCCAGAGTTCCTTACCATGATGGCCCGTAAGATGAAGGATACCGACTCCGAGGAGGAGATCCGTGAGGCCTTCAAGGTCTTCGATCGCGATAACAACGGCTTCATCTCTGCCGCGGAGCTGCGCCACGTCATGACCTCTATCGGTGAGAAGCTCACCGATGACGAAGTCGATGAGATGATCCGTGAGGCGGACCAGGACGGGGACGGCCGTATTGACTGTATGTTTCACACATTCCCTGCTTGAGTGTCCGTCCGATGACTAATTTTTGCTCCTTCCTCCAGACAATGAGTTTGTCCA |
| Aspergillus pseudotamarii | > Aspergillus pseudotamarii NRRL 25517 ITS  AAGGATCATTACCGAGTGTAGGGTTCCTAGCGAGCCCAACCTCCCACCCGTGTTTACTGTAACCTTAGTTGCTTCGGCGGGCCCGCCGTTTACGGCCGCCGGGGGGCATCAGCCCCCGGGCCCGCGCCCGCCGGAGACACCACGAACTCTGTCTGATCTAGTGAAGTCTGAGTTGATTGTATCGCAATCAGTTAAAACTTTCAACAATGGATCTCTTGGTTCCGGCATCGATGAAGAACGCAGCGAAATGCGATAACTAGTGTGAATTGCAGAATTCCGTGAATCATCGAGTCTTTGAACGCACATTGCGCCCCCTGGTATTCCGGGGGGCATGCCTGTCCGAGCGTCATTGCTGCCCATCAAGCACGGCTTGTGTGTTGGGTCGTCGTCCCCTCTCCGGGGGGGACGGGCCCCAAAGGCAGCGGCGGCACCGCGTCCGATCCTCGAGCGTATGGGGCTTTGTCACCCGCTCTGTAGGCCCGGCCGGCGCTTGCCGAACGCAAAACAACCATTCTTTCCA | > Aspergillus pseudotamarii isolate NRRL 443 beta-tubulin gene  TGGTATGTCTCGATGCCTTCGAGTTAATATGCTTTGGACCAAGGAACTTCACAGAAGCATGAACTCGGATGTGCCCTATTGCGTTTGCTAACATCCTTGCAGGCAGACCATCTCTGGCGAGCACGGCCTTGACGGCTCCGGTGTGTAAGTACAGTCCGTGTACACCTCGAACGAACGACGAGCAGATGGCATTGGAAGAGTTGGAATGGGTCTGACGGGAAGGATAGTTACAATGGCTCCTCTGATCTCCAGCTGGAGCGTATGAACGTCTACTTCAACGAGGTGCGTACCTCAAATTTTTCAGCCTCTATGAAAACGCTTTGCAAGTCCTGACCGCTTCTCCAGGCCAGCGGAAACAAGTATGTCCCTCGTGCCGTCCTCGTCGATCTTGAGCCCGGTACCATGGACGCCGTCCGTGCCGGTCCCTTCGGTCAGCTGTTCCGTCCCGACAACTTCGTTTTCGGCCAGTCCGGTGCTGGTAACAACTGG | > Aspergillus pseudotamarii isolate NRRL 443 calmodulin gene  CCGAGTACAAGGAGGCCTTCTCCCTATTCGTAAGTCATGATCATCGTTCGCGAAAATCGGTTTTTATTAGTCGTCTTTATTTGAACACAAGCTGACTGGGATTCTCTTGGGTTTCCTATAGGACAAGGACGGTGATGGTTAGTACAGTTATATTTCATTCCGTCTCCCTTCAAGTGCGACCAATATCTTTTAGCCGCCATAATTTTATCCATTTTCTGTTCGATCGGCTGAAGTCTTGGCATTGATGGATTGACTTGATATGCAGGCCAGATCACCACCAAGGAGTTGGGCACTGTCATGCGCTCTCTGGGCCAAAACCCGTCTGAGTCGGAACTCCAGGACATGATTAACGAGGTTGACGCCGATAACAATGGCACCATTGACTTTCCTGGTACGAGAGGGCTTCCGTACGATTCACAAATAATATAGCTGTTAATATTCAATCAGAGTTCCTCACGATGATGGCGAGAAAGATGAAGGATACCGACTCTGAGGAGGAGATCCGGGAGGCTTTCAAGGTTTTCGACCGCGATAACAACGGTTTCATCTCCGCTGCCGAATTGCGCCACGTCATGACCTCCATCGGCGAGAAGCTTACTGATGACGAAGTTGATGAGATGATCCGCGAAGCGGATCAGGATGGTGACGGTCGGATTGACTGTACGTTTCGAGAAGCCCTCCCACACCTATTGCGGCTGTGAAACCGGTGATACTGATCGATTTTAGACAACGAGTTC |
| Aspergillus caelatus | > Aspergillus caelatus IMI 375694 ITS  CCCAACCTCCCACCCGTGTTTACTGTAACCTTAGTTGCTTCGGCGGGCCCGCCGTTTACGGCCGCCGGGGGGCATCAGCCCCCGGGCCCGCGCCCGCCGGAGACACCACGAACTCTGTCTGATCTAGTGAAGTCTGAGTTGATTGTATCGCAATCAGTTAAAACTTTCAACAATGGATCTCTTGGTTCCGGCATCGATGAAGAACGCAGCGAAATGCGATAACTAGTGTGAATTGCAGAATTCCGTGAATCATCGAGTCTTTGAACGCACATTGCGCCCCCTGGTATTCCGGGGGGCATGCCTGTCCGAGCGTCATTGCTGCCCATCAAGCACGGCTTGTGTGTTGGGTCGTCGTCCCCTCTCCGGGGGGGACGGGCCCCAAAGGCAGCGGCGGCACCGCGTCCGATCCTCGAGCGTATGGGGCTTTGTCAC | > Aspergillus caelatus isolate NRRL 25528 beta-tubulin gene  TGGTATGTCTCGATGCCTTCGAGTTAATGTGCTTTGGGCCAAGGAACTCCACAGAAGCATGAACTCGGATGTGCCCTATTGTGTCTGCCACGTGTTTGCTAACATCTTTGCAGGCAGACCATCTCTGGCGAGCATGGCCTTGACGGCTCCGGTGTGTAAGTACAATCCGTGTACACCTCGAACAAACGACGACCAGATGGCATTGGAAGAGTTGGAATGGGTCTGACGGGAAGGATAGTTACAATGGCTCCTCCGATCTCCAGCTGGAGCGTATGAACGTCTACTTCAACGAGGTGCGTACCTCAAAATTTTCAGCCTCTATAAAAACGCTTTGCAAGTCCTGACCGCTTCTCCAGGCCAGCGGAAACAAGTATGTCCCTCGTGCCGTCCTCGTCGATCTTGAGCCCGGTACCATGGACGCCGTCCGTGCCGGTCCCTTCGGTCAGCTGTTCCGTCCCGACAACTTCGTTTTCGGCCAGTCCGGTGCTGGTAACAACTGG | > Aspergillus caelatus isolate NRRL 25528 calmodulin gene  CCGAGTACAAGGAGGCCTTCTCCCTATTCGTAAGTCATGATCGTCGTTCGCGAAAATTGGTTTTTGTTAGTCGTCTTTATTTGAACACAAGCTGACTGGGCTTCTCTTGGGTTTGCTATAGGACAAGGACGGTGATGGTTAGTACAGTCTATTTTATTCCGTCTCCCTTCAAGTGCGACCAATATCTTTTAGCCGCCATAATTTTATCCATTTTCTGTTCGATCGGCTGAAGTCTTGGCATTGATGGATTGACTTGATATGCAGGCCAGATCACCACCAAGGAGTTGGGCACTGTCATGCGCTCTCTGGGTCAAAACCCCTCTGAGTCGGAACTCCAGGACATGATTAACGAGGTTGACGCCGATAACAATGGCACCATTGATTTTCCTGGTACGAAAGGGCTTCCGTACGATTCACAAATAAAATAGCTGTTAATGTTCAATCAGAGTTCCTCACGATGATGGCGAGAAAGATGAAGGATACCGACTCTGAGGAGGAGATCCGGGAGGCTTTCAAGGTTTTCGACCGCGATAACAACGGTTTCATCTCCGCTGCCGAATTGCGCCACGTCATGACCTCTATCGGCGAGAAGCTTACCGATGACGAAGTTGATGAGATGATCCGCGAGGCGGATCAGGATGGTGACGGTCGGATTGACTGTACGTTTCGAGAAGCCCTCCCAGACCTATTGCGGCTGTGAAACCGGTGATACTGATTGATTATAGACAACGAGTTC |
| Aspergillus sojae | > Aspergillus sojae CBS 100928 ITS region  TCCTAGCGAGCCCAACCTCCCACCCGTGTTTACTGTACCTTAGTTGCTTCGGCGGGCCCGCCGTCATGGCCGCCGGGGGCGTCAGCCCCGGGCCCGCGCCCGCCGGAGACACCACGAACTCTGTCTGATCTAGTGAAGTCTGAGTTGATTGTATCGCAATCAGTTAAAACTTTCAACAATGGATCTCTTGGTTCCGGCATCGATGAAGAACGCAGCGAAATGCGATAACTAGTGTGAATTGCAGAATTCCGTGAATCATCGAGTCTTTGAACGCACATTGCGCCCCCTGGTATTCCGGGGGGCATGCCTGTCCGAGCGTCATTGCTGCCCATCAAGCACGGCTTGTGTGTTGGGTCGTCGTCCCCTCTCCGGGGGGGACGGGCCCCAAAGGCAGCGGCGGCACCGCGTCCGATCCTCGAGCGTATGGGGCTTTGTCACCCGCTCTGTAGGCCCGGCCGGCGCTTGCCGAACGCAAAACAACCATTTTTTCCA | >Aspergillus sojae isolate SMF118 beta-tubulin (benA) gene  TGGTATGTCTCGATACCTTCGAGTTAATATGCTTTGGACCAAGGAACTCCTCCAAAGCATGATCTCGGATGTGTCCTATTATATCTGCCACGTGTTTGCTAACAACTTTGCAGGCAAACCATCTCTGGCGAGCACGGCCTTGACGGCTCCGGTGTGTAAGTACAGCCTGTATACACCTCGAACGAACGACGACCATATGGCATTAGAAGTTGGAATGGATCTGACGGCAAGGATAGTTACAATGGCTCCTCTGATCTCCAGCTGGAGCGTATGAACGTCTACTTCAACGAGGTGCGTACCTCAAAATTTTCAGCATCTATGAAAACGCTTTACAACTCCTGACCGCTTCTCCAGGCCAGCGGAAACAAGTATGTCCCTCGTGCCGTCCTCGTCGATCTTGAGCCTGGTACCATGGACGCCGTCCGTGCCGGTCCCTTCGGTCAGCTCTTCCGTCCCGACAACTTCGTTTTCGGCCAGTCCGGTGCT | > Aspergillus sojae strain DTO 173-C3 calmodulin (CaM) gene,  TCGTAAGTAGTTATCGTCGTTCGCGAAAATTGGTTTTTGTTAGTCGTCTTGATTTGAACACAAGCTGACTTGGCTTTTCTTGGGTTTCCTATAGGACAAGGACGGTGATGGTTAGTACAGTTTATTTTATTCATTCTCCCTTCAAATGCGACCAATATGTTTTGGCCGCCATAAGTTTATCAAGTTTCTGTTCGATCGGCTGAAGTCTTGGCATTGATGAATTGACTTGATATGCAGGCCAGATCACCACCAAGGAGTTGGGCACTGTCATGCGCTCTCTGGGTCAAAACCCCTCTGAGTCGGAACTCCAGGACATGATTAACGAGGTTGACGCCGACAACAATGGCACCATTGACTTCCCTGGTACGAGACGGCTTCGTACGATTCATAAATGAAATAGTTGTTAATCGTCCAAATAGAATTCCTTACGATGATGGCGAGAAAGATGAAGGATACCGACTCTGAGGAGGAGATCCGGGAGGCTTTCAAGGTTTTCGACCGCGATAACAACGGCTTCATCTCCGCTGCCGAA |
| Aspergillus udagawae | >Aspergillus udagawae CBM FA-0702 ITS region;  CCGAGTGAGGGCCCTCTGGGTCCAACCTCCCACCCGTGTCTATCGTACCTTGTTGCTTCGGCGGGCCCGCCGTTTCGACGGCCGCCGGGGAGGCCTCGCGCCCCCGGGCCCGCGCCCGCCGAAGACCCCAACATGAACTCTGTTCTGAAAGTATGCAGTCTGAGTTGATTATCATAATCAGTTAAAACTTTCAACAACGGATCTCTTGGTTCCGGCATCGATGAAGAACGCAGCGAAATGCGATAAGTAATGTGAATTGCAGAATTCAGTGAATCATCGAGTCTTTGAACGCACATTGCGCCCCCTGGTATTCCGGGGGGCATGCCTGTCCGAGCGTCATTGCTGCCCTCAAGCACGGCTTGTGTGTTGGGCCCCCGTCCCCGGTTTCCCCCGGGGACGGGCCCGAAAGGCAGCGGCGGCACCGCGTCCGGTCCTCGAGCGTATGGGGCTTTGTCACCCGCTCTGTAGGCCCGGCCGGCGCCAGCCGACACCCAACTTTATTTCCTAAGG | >Aspergillus udagawae isolate 18-058-04848 beta-tubulin (benA) gene,  GGTCAGTGTGTAAGTACTGCCCATCGTGGGTGATGGACTGTTATGGGAGAAGCGTAGGGATGGCGTTTGCATTGAGCTGAGACTAAACTCGATTCTTGGTGATAGGGTAACCAAATTGGTGCTGCTTTCTGGTATGCCTCGACCTCAATGCTTGGATGATGGGTGATTAGGACCTGTCAACCTAGCAGGCTGTCCTCCATGGTTTCAGCGTCGCTGTGATGGGTATCAGCTAACAAATTTATAGGCAGACCATCTCTGGTGAGCACGGCCTTGACGGCTCTGGCCAGTAAGTTCGACCTTTATCCTCCCAATCGAGAAAGCGGGGGAAACACGAAAGCAAGCAGGAAGAGGGCGCGTGTCTGATGAGGATAATAGCTACAATGGCTCCTCCGATCTCCAGCTGGAGCGTATGAACGTCTATTTCAACGAGGTGCGTGGATGAAACTCGACTCTACACTACTTCGGCAACATCTCACGATCTGACTCGCTACTAGGCCAACGGCGACAAATATGTTCCTCGTGCCGTTCTGGTCGATCTCGAGCCCGGTACCATGGACGCTGTCCGTGCCGGTCCCTTCGGCGAGCTATT | >Aspergillus udagawae strain det 18.043M calmodulin (CaM) gene,  TTCTCTCTCTTCGTAAGTGAACTGTTTTAGTCCCTAGTCGTTGTATAAGAAGAATTTCCACAATATTGAGAGTTTGCGCTCACACGAGATTCGACCTATAGGACAAGGATGGTGATGGTTAGTGACCTTTTTTTCCGCTCCTCGAACGTCGGATTCTGTGCGATCATGTTCAAATGCCAACTTACAATATCCGAGAATGGCCCCATCAATACTGATAATATCTATGTTTGACTATTAGGCCAGATCACCACCAAGGAACTGGGCACTGTGATGCGCTCCCTGGGCCAGAACCCTTCCGAGTCAGAGCTGCAAGATATGATCAACGAGGTGGATGCTGACAACAACGGCACCATCGATTTCCCTGGTACGCGGATGCATCTAGCGTGACCCGGGAGGGAGGAGAACAATCATTAACTCGTATACAGAATTCCTTACAATGATGGCTCGGAAGATGAAGGACACCGACTCCGAAGAGGAAATCCGGGAAGCTTTTAAGGTCTTCGACCGCGACAACAACGGTTTCATTTCCGCTGCGGAGCTGCGCCACG |
| Aspergillus westlandensis | > Aspergillus westlandensis ITS region  AAGGATCATTACTGAGTGAGGGTCCCTCGGGGCCCAACCTCCCACCCGTGTATACCGTACCTTGTTGCTTCGGCGGGCCCGCCGTTTACGCGGCCGCCGGGGGGCTAACCCCCCCGGGCGAGCGCCCGCCGGAGACACCAACGTGAACACTGTCTGAAGTTTTGTTTGTCTGAGTCGATTGTATCGCAATCAGTTAAAACTTTCAACAATGGATCTCTTGGTTCCGGCATCGATGAAGAACGCAGCGAAATGCGATAATTAATGTGAATTGCAGAATTCAGTGAATCATCGAGTCTTTGAACGCACATTGCACCCCCTGGTATTCCGGGGGGTATGCCTGTCCGAGCGTCATTGCTGCCCTCAAGCACGGCTTGTGTGTTGGGTCGTCGTCCCCCCCGGGGGACGGGCCCGAAAGGCAGCGGCGGCACCGCGTCCGGTCCTCGAGCGTATGGGGCTTTGTCACCCGCTCTTGTAGGCCCGGCCGGCGCTGGCCGACGCTGAAAAGCAACCATTATTTCTCCA | >Aspergillus westlandensis strain DTO:231-B1 beta-tubulin (tub2) gene,  GTTATCAAGGATCGCCTCCTCACACATTGGCTTCGATGATGCCCAATCCTTGATACTTGGACACTAACAGAGTGACTAGGCAACAAATCTCTGGCGAGCACGGCCTTGACGGCGCCGGTGTGTAAGTACAACCCGCATTTACACCTGCCGAAATCAAATTCGAGGAGAAAAGAAGGAAACGGCCACGGTGGAATTGATGGTCTGATGGGATAAACAGTTACAATGGCTCCTCCGACCTCCAGCTGGAGCGCATGAACGTCTACTTCAACGAGGTTCGTTGCCCGAAAATTTCACATCTCCTCTTCGCCTATCCGAAACGCACCCCCGTGCAAGGTTCTGACCCACGTTTTGTTCATCTTCTAGGCCACCGGTGGCAAGTATGTTCCCCGTGCCGTTCTGGTCGATCTTGAGCCCGGTACCATGGACGCTGTCCGTGCCGGTCCCTTCGGCCAGCTTTTCCGCCCCGACAACTTCGTCTTCGGCCAGTCTGGTGCCGGTAACAACT | >Aspergillus westlandensis strain DTO:231-B1 calmodulin (cmdA)  AATAATGAAAGCTCACATGAGATTTTGCGTCTCTTAACAGGACAAGGATGGCGATGGTTAGTGCTATTCGGTCTCCCTTCCAGTTTATCACCCTCACGCGACCGGCCATCTCTCGTCGATATGGAGATTTTTTTTTTTTTTCGATGGTCCCCCGCGATCGAATTCGCAAGCAGCAGGCTAATTTTTTGAATTCACGCTGCAGGCCAGATCACCACCAAGGAGCTGGGCACTGTGATGCGCTCGCTGGGCCAGAACCCCTCCGAGTCTGAGTTGCAGGACATGATCAACGAGGTTGACGCCGACAACAACGGCACCATTGATTTCCCCGGTATGCGATGATGGACAGGTCGGGTTCCGGAAAGCGAAACCAGCTGCTAACTCGCCCACGCAGAGTTCCTGACGATGATGGCCCGAAAGATGAAGGACACCGATTCTGAGGAGGAAATCCGG |
| Aspergillus carbonarius | >Aspergillus carbonarius CBS 111.26 ITS region;  TAAAAGTCGTAACAAGGTTTCCGTAGGTGAACCTGCGGAAGGATCATTACCGAGTGCGGGTCCTTTGGGCCCAACCTCCCACCCGTGTCTATTGTACCCTGTTGCTTCGGCGGGCCCGCCGCTTGTCGGCCGCCGGGGGGGCATCTCTGCCCCTCGGGCCCGTGCCCGCCGGAGACACCAACACGAACACTGTCTGAAATCGTGAAGTCTGAGTCGATTGTTTTCAATCAGTTAAAACTTTCAACAATGGATCTCTTGGTTCCGGCATCGATGAAGAACGCAGCGAAATGCGATAACTAATGTGAATTGCAGAATTCAGTGAATCATCGAGTCTTTGAACGCACATTGCGCCCCCTGGTATTCCGGGGGGCATGCCTGTCCGAGCGTCATTGCTGCCCTCAAGCCCGGCTTGTGTGTTGGGTCGCCGTCCCCCTGTCTGGGGGGACGGGCCCGAAAGGCAGCGGCGGCACCGCGTCCGATCCTCGAGCGTATGGGGCTTTGTCACATGCTCTGTAGGATTGGCCGGCGCCTGCCGACAACTCCAACCTTTTTTTCCAGGTTGACCTCGGATCAGGTAGGGATACCCGCTGAACTTAAGCATATCA | >Aspergillus carbonarius strain A-2160 beta-tubulin gene  TCGGTGCTGCTTTCTGGTATGTCTCCCGGTCACTAGACTTGGGATGGGATCTATCTCAGGCTTTGTAGCATGAGTCTAGATGCCCATTGTTACTCTTGCTTCGGAATTCATCTAACAACCCAACAGGCAGACCATCTCTGGTGAGCACGGCCTCGACGGCACCGGTGTGTAAGTACACCCTTTTCACGTCTCAATCGGTCAACATAACGGAAGAATCGGGTTCCTGACGTGGAGGATAGTTACAATGGCACCTCCGATCTCCAGCTGGAGCGCATGAACGTCTACTTCAACGAGGTGGGTGTCAACCAATCCTAGGTTTTACCACGTGTACATGTGGTGTCTCGATTCCTGACTCCTTCACCAGGCTAGCGGCAACAAGTATGTTCCTCGTGCCGTCCTTGTCGACCTTGAGCCCGGTACCATGGATGCCGTCCGTGCCGGTCCTTTCGGTCAGCTCTTCCGCCCCGACAACTTCGTCTTCGGCCAGTCCGGTGCTGGTAACAACTGGGCCAAGGGTCACTACACTGAGGGT | >Aspergillus carbonarius partial caM gene for calmodulin, isolate CCF 3388,  GAAGAGCAAGTTTCTGAATACAAGGAGGCCTTCTCCCTCTTTGTAAGTACCTCCTGAATGTCTTCCGACCAGCGAAAGCGATCGATAGTCCACAAGAATACACGCTCATATGCTCTAATCCAATAGGACAAGGATGGCGATGGTGGGTGGAATCCTATTCCCTTCATATTTACCTATGGCGACCGAACCGACCGCGGGTTTCCGAAAGCCGTTTTCCAAGCGACTTGAGCCGTGATACTGATGTGACATGGGAAATAGGCCAGATCACCACCAAGGAGCTGGGCACTGTCATGCGCTCGCTCGGCCAGAACCCCTCTGAGTCTGAGCTTCAGGACATGATCAATGAGGTCGATGCTGACAACAACGGCACGATCGACTTCCCCGGTATGTGGCGGGCTTGTGTTCACACGAGGCAAGAATGCGAACCGGATATTAACTTCTGCCAGAATTCCTTACCATGATGGCTCGTAAGATGAAGGACACCGACTCCGAGGAGGAGATTCGCGAGGCTTTCAAGGTCTTCGACCGCGATAACAACGGTTTTATCTCCGCCGCTGAGCTGCGTCATGTCATGACCTCCATCGGTGAGAAGCTCACCGACGACGAGGTCGATGAGATGATCCGTGAGGCGGACCAGGACGGCGACGGCCGCATTGATTGTATGTTTTCTATCGACAACCATCTGCGT |
| Aspergillus phoenicis | > Aspergillus phoenicis genes for, strain: RIB 2622  CCGAGTGCGGGTCCTTTGGGCCCAACCTCCCATCCGTGTCTATTGTACCCTGTTGCTTCGGCGGGCCCGCCGCTTGTCGGCCGCCGGGGGGGCGCCTCTGCCCCCCGGGCCCGTGCCCGCCGGAGACCCCAACACGAACACTGTCTGAAAGCGTGCAGTCTGAGTTGATTGAATGCAATCAGTTAAAACTTTCAACAATGGATCTCTTGGTTCCGGCATCGATGAAGAACGCAGCGAAATGCGATAACTAATGTGAATTGCAGAATTCAGTGAATCATCGAGTCTTTGAACGCACATTGCGCCCCCTGGTATTCCGGGGGGCATGCCTGTCCGAGCGTCATTGCTGCCCTCAAGCCCGGCTTGTGTGTTGGGTCGCCGTCCCCCTCTCCGGGGGGACGGGCCCGAAAGGCAGCGGCGGCACCGCGTCCGATCCTCGAGCGTATGGGGCTTTGTCACATGCTCTGTAGGATTGGCCGGCGCCTGCCGACGTTTTCCAACCATTCTTTCCAGG | >Aspergillus phoenicis strain NRRL 4757 bt gene, beta-tubulin gene,  TGGTACGTATTCACTGCCACTGGATTGGGGATGGAACATCATCTCTCAAGCTATCTTAGCTTGAGTTCAGATGTTATCCATCGGGTATATAGCTATCGGGTTAAGAACACGTCTAACAACTCAACAGGCAGACCATCTCTGGCGAGCACGGCCTTGACGGCTCCGGTGTGTAAGTACAACTTTTTCACACCTCTCAATTGGTCAACAATGTGGAAAGGATTGGGTTTCCTGACGCGCAGGATAGTTACAATGGCACCTCCGACCTCCAGCTGGAGCGCATGAACGTCTACTTCAACGAGGTTAGATCACACCGTCCCTGAGTTTTTTCACGACAATATCATCAATGTCCTGACCACTTCAGCAGGCTAGCGGTAACAAGTATGTCCCCCGTGCCGTCCTCGTCGATCTCGAGCCCGGTACCATGGACGCCGTCCGTGCCGGTCCCTTCGGCCAGCTCTTCCGCCCCGACAACTTCGTCTTCGGCCAGTCCGGTGCTGGTAACAACTGG | >Aspergillus phoenicis isolate N4018 calmodulin (cmd) gene,  CCCTTCACGTTTTACCTGTAGCGCCCGATCCGACCGCGGGATTTCGACAGCTATTTCCCCCTTCGATCTGAATCATAATACTGATGTAATCTGGAAATAGGCCAGATCACCACCAAGGAGCTCGGCACTGTGATGCGCTCCCTCGGCCAGAACCCCTCCGAGTCTGAGCTTCAGGACATGATCAACGAGGTTGACGCTGACAACAACGGAACGATCGACTTCCCCGGTATGTGATAGATCTACGCCTGTAAGGCGGGAATGCCGTATGGATTGTGATTGACTTTTGCCGCCAGAATTCCTTACCATGATGGCTCGTAAGATGAAGGACACCGACTCCGAGGAGGAAATCCGCGAGGCTTTCAAGGTCTTCGACCGCGACAACAATGGTTTCATCTCCGCCGCGGAGTTGCGCCACGTCATGACCTCCATTGGCGAGAAGCTCACTGACGACGAAGTCGATGAGATGATCCGTGAGGCTGACCAGGACGGTGATGGCCGCATCGACTGTATGTTTCCCATTCCTGATATGCCCGTGATATGACATGCTAACTCTGCTACCAGACAACGAGTTCGTC |
| Aspergillus thermomutatus | >Aspergillus thermomutatus NRRL 20748 ITS  AAGGATCATTACCGAGTGAGGGCCCTCTGGGTCCAACCTCCCACCCGTGTCTATTGTACCTTGTTGCTTCGGCGGGCCCGCCGTTTCTACGGCCGCCGGGGAGGCCTCGCGCCCCCGGGCCCGCGCCCGCCGAAGACCACAACATGAACGCTGTTCTGAAAGTATGCAGTCTGAGTCGATTATCATAATCAGTTAAAACTTTCAACAACGGATCTCTTGGTTCCGGCATCGATGAAGAACGCAGCGAAATGCGATAAGTAATGTGAATTGCAGAATTCAGTGAATCATCGAGTCTTTGAACGCACATTGCGCCCCCTGGTATTCCGGGGGGCATGCCTGTCCGAGCGTCATTGCTGCCCTCAAGCACGGCTTGTGTGTTGGGCCGCCGTCCCCGGTTTCTCCCCGGGGACGGGCCCGAAAGGCAGCGGCGGCACCGCGTCCGGTCCTCGAGCGTATGGGGCTTCGTCACCCGCTCTGTAGGCCCGGCCGGCGCCAGCCGACGACCAACCCAACTTTTCTAA | > Aspergillus thermomutatus isolate TLS66 beta-tubulin-like (benA)  TGGTATGTCTTGATCTCAATGCTCGGATGATGGAAGATGAGGACCAGGCTGTCCTCCATGGGCTCTGGTTCGCTGTCATGGGTATCAGCTGACAAATTTACAGGCAGACCATCTCTGGTGAGCACGGCCTCGACGGCTCTGGCCAGTAAGTTCGACCTTTGTCCTCCCAATTGAGAAAGCGGGGGAAACACGAAAGGCAAGCAGGAAGAGGACCCGTGTCTGATGGGGATAATAGCTACAATGGCTCCTCCGATCTCCAGCTGGAGCGTATGAACGTCTATTTCAACGAGGTGCGTGGATGAAACTCTCCGGTCGACGCTATTTCGGGCAACATCTCATGATCTGACTTGCTACCAGGCCAACGGTGACAAGTATGTTCCTCGTGCCGTCCTGGTCGATCTCGAGCCCGGTACCATGGACGCTGTCCGTGCCGGTCCCTTCGGCGAGCTCTTCCGTCCCGACAACTTCGTCTTCGGCCAGTCTGGTGCTGGTAACAACTGG | > Aspergillus thermomutatus gene for calmodulin, isolate FWiP_155  GAGGAGCAGGTTTCCGAGTACAAGGAGGCTTTCTCTCTTTTCGTAAGTGCACTGTTCTAGCCCCTAGTCGTTGCATAAGTGGAATTTCCACAATGTTGAGGATGTGCGCTCACATGAGATTCGACCTATAGGACAAGGATGGTGATGGTTAGTGAACCGCTCCTCGAACGTCTGCTTCCGTGCGACCGTGTGCAAATGCTCAATATCGCGAAATGATCCATCAATACTGATATATCTATGTTTGACTCTTAGGCCAGATCACCACCAAGGAATTGGGCACTGTTATGCGCTCCCTGGGCCAGAACCCTTCCGAGTCGGAGCTGCAAGATATGATCAACGAGGTGGATGCTGACAACAATGGCACCATCGATTTCCCTGGTATGCGATGCATCTGGCATGACTTGGCCGGGAGGGAGGAGAACAACCGCTAACCCGTACTCAGAATTCCTTACCATGATGGCTCGGAAGATGAAGGATACCGACTCCGAAGAGGAAATCCGGGAAGCCTTCAAGGTTTTCGACCGCGACAACAACGGTTTCATTTCCGCTGCGGAGCTGCGCCACGTTATGACCTCTATCGGAGAGAAGCTCACTGATGACGAAGTTGACGAGATGATCCGCGAGGCGGATCAGGATGGTGACGGCCGGATTGATTGTATGTTGATTAAACCGCTTTGCTCTGTTGAATCCCCGGAATATTTCGCTAATTGTTTGTAGACAACGAGTTCGTCCA |
| Aspergillus brunneoviolaceus | > Aspergillus brunneoviolaceus NRRL 4912 ITS region;  CGAGTGCTGGGTCCTTCGGGGCCCAACCTCCCACCCGTGCTTACCGTACCCTGTTGCTTCGGCGGGCCCGCCTTCGGGCGGCCCGGGGCCTGCCCCCGGGACCGCGCCCGCCGGAGACCCCAATGGAACACTGTCTGAAAGCGTGCAGTCTGAGTCGATTGATACCAATCAGTCAAAACTTTCAACAATGGATCTCTTGGTTCCGGCATCGATGAAGAACGCAGCGAAATGCGATAACTAATGTGAATTGCAGAATTCAGTGAATCATCGAGTCTTTGAACGCACATTGCGCCCCCTGGTATTCCGGGGGGCATGCCTGTCCGAGCGTCATTTCTCCCCTCCAGCCCCGCTGGTTGTTGGGCCGCGCCCCCCCGGGGGCGGGCCTCGAGAGAAACGGCGGCACCGTCCGGTCCTCGAGCGTATGGGGCTCTGTCACCCGCTCTATGGGCCCGGCCGGGGCTTGCCTCGACCCCCAATCTTCTCA | > Aspergillus brunneoviolaceus partial benA gene for beta-tubulin, strain IHEM 4062  TGTGTAAGTGAATTCGTGCTGGGACACACAACCTTTGCGGTTCGATATATTCATGCAGTGGACTAAAACATGGGTGCTGGTGACAGGGTAACCAAATTGGTGCCGCTTTCTGGTATGTCTCTACTCGAGTGAATGGATAGATGACATGTTCTGTAGACTCTCACCCAGAGTCAAGAACATTACTGTCAAAGCTTCTCTGTGGTCAATTGAGCTAACAATCTTCCAGGCAGACCATCTCTGGTGAGCACGGCCTCGATGGCGCCGGTGTGTAAGTGCAGACTTCCCGGGCGGCAGGACCAGCGGGTCGCGGAAAATGGTGAAGGATATTAATGGAAAGGACAGTTACAATGGCTCCTCCGACCTCCAGCTGGAGCGCATGAACGTCTACTTCAACGAGGTTCGTCTTCGAGTCCTCCCTCCGGCTTTCCCGCATCAGCTCCTGACTGCTTCACCAGGCTAGCGGCAACAAGTATGTTCCCCGTGCCGTCCTCGTCGACCTCGAGCCCGGTACCATGGACGCCGTCCGTGCCGGTCCTTTCGGCCAGCTTTTCCGCCCCGACAACTTCGTCTTCGGTCAATCCGGTGCTGGTAACAACTGGGCCAAGGGTC | > Aspergillus brunneoviolaceus PW4048 CaM gene for calmodulin,  AGAGCAAGTTTCCGAGTACAAGGAGGCCTTCTCCCTTTTTGTAAGCGACCCCGATGCTTTCTCGTCTGCTATAAAACAATGTTTACTGATTCGTTTCTCGTGATCCCTGATAGGACAAGGATGGCGATGGTTAGTGTAGTTCTTTACCCCTCAACGAAACCCGCCTGGCATCACCTGGCACTATAGCCACCATACCGCAAACGCCACAGAAACTATCAAGAATCGATCGCATTTCTGACAGAATCCATGCTACAGGACAAATTACCACCAAGGAGTTGGGTACCGTTATGCGCTCTCTCGGCCAGAACCCCTCCGAGTCTGAGCTCCAGGACATGATCAACGAGGTTGACGCTGACAACAACGGCACTATCGACTTTCCCGGTATGTGATGGCCATGCATCGAGCTCCGGACATAGTATTGACAGCTGCCCAGAATTCCTTACCATGATGGCCCGTAAGATGAAGGATACCGACTCTGAGGAGGAGATCCGTGAGGCCTTCAAGGTCTTCGATCGCGATAACAACGGCTTCATCTCTGCCGCGGAGCTGCGCCACGTCATGACCTCTATCGGCGAGAAGCTCACCGATGACGAAGTCGATGAGATGATCCGTGAGGCGGACCAGGATGGTGACGGCCGTATTGACTGTATGTTTCACACATTCCCTGCTTGAGTGTCCGCCCGATGACTAATTTTCGCTCCTTCCTCCAGACAATGAGTTT |
| Aspergillus leporis | >Aspergillus leporis NRRL 3216 ITS region; AAGGATCATTACCGAGTGAGGGTTCCACTAGGGCCCAACCTCCCACCCGTGTTTATTACTACCTTGTTGCTTCGGCGGGCCCGCCGCAAGGCCGCCGGGGGGCATCAGCTCCCTGGCCCGCGCCCGCCGGAGACACCTGAACACTGTTTGATACCATGCAGTCTGAGTTGATTGTCTTGCAATCAGTTAAAACTTTCAACAATGGATCTCTTGGTTCCGGGATCGATGAAGAACGCAGCGAAATGCGATAACTAATGTGAATTGCAGAATTCCGTGAATCATCGAGTCTTTGAACGCACATTGCGCCCCCTGGTATTCCGGGGGGCATGCCTGTCCGAGCGTCATTGCTGCCCATCAAGCACGGCTTGTGTGTTGGGTCCCGTCCCCCTCTCCCGGGGGACGGACCCGAAAGGCAGCGGCGGCACCGCGTCCGGTCCTCGAGCGTATGGGGCTTTGTCACCCGCTCTTGTAGGCCCGGCCGGCGCTTGCCGACACCCAACCATTTTTTTCAGGTTGACCTCGGATCAGGTAGGGATACCCGCTGAACTTAAGCATATCAATAAGCGGAGGAAAAGAAACCAACCGGGATTGCCTCAGTAACGGCGAGTGAA | > Aspergillus leporis strain DTO 303-C5 beta-tubulin (BenA) gene,  TGGTATGTCTCAGCCGACGGCATAATATGTCTTGGACAAAAGGACTTGGCGGAAGCATAGTCTGATATGTTCTGTCACATTGGTCATGTGTTTTGCTAACAGCTTCGCAGGCAGACCATCTCTGGCGAGCACGGCCTTGATGGCTCCGGTGTGTAAGTACAAACTGCGTATATATCGAATGGAGGACAATTAGATGGCAGTGGAATAGTTCGAAAGGTCTGACGGAAAGGATAGCTACAATGGCTCCTCCGATCTTCAGCTGGAGCGCATGAACGTCTACTTCAACGAGGTTCGTACCTCAGAATTTTTCTGGATTCAAGCTTCCGCGAAAACATTTGCAGTTCCTGACCTCTTCTCCAGGCTAGCGGAAACAAGTATGTTCCTCGTGCCGTCCTCGTCGATCTGGAGCCCGGTACCATGGACGCTGTCCGTGCCGGCCCCTTCGGTCAGCTCTTCCGTCCCGACAACTTCGTTTTCGGCCAGTCCGGTGCTGGTAACAACTGGGCCAAG | >MG518130.1 Aspergillus leporis strain DTO 303-C5 calmodulin (CaM) gene, partial cds  TCGTAAGTTGTTTTATCCGTTCCCGAAACATGGTCCTTTTGCGTGGGCTTTGTAGAGTACATGCTTACATGCCCTTTTTGGGGCTCTCAATAGGACAAGGATGGCGATGGTTAGTACAAATACGATTCCTTTTCAGTGCGACCGACAGTTTTCAATCCCGCAAATCCCATATCATTATTTTGTTCGGTCGGCTCAAGTCTTGGCATTGGTAAATTGACTCGATATGCAGGCCAGATCACCACCAAGGAGTTGGGCACTGTGATGCGCTCGCTGGGTCAGAACCCTTCTGAGTCGGAACTCCAGGACATGATCAACGAGGTTGACGCCGATAACAATGGCACCATCGACTTCCCTGGTACGCGAGAGCCTTAATGCGGCCCAAATGCAGAATCAGTTGTTAATTTCTGATTAGAGTTCCTTACGATGATGGCGAGAAAGATGAAGGACACCGACTCTGAGGAGGAGATCCGGGAGGCTTTCAAGGTCTTCGACCGCGACAACAATGGCTTCATTTCTGCTGCGGAG |
| Aspergillus pseudoviridinutans | > Aspergillus pseudoviridinutans isolate LSPQ-01141 ITS  AAGGATCATTACCGAGTGAGGGCCCTCTGGGTCCAACCTCCCACCCGTGTCTATTGTACCTTGTTGCTTCGGCGGGCCCGCCGTTTCGACGGCCGCCGGGGAGGCCTCGCGCCCCCGGGCCCGCGCCCGCCGAAGACCCCAACATGAACGCTGTTCTGAAAGTATGCAGTCTGAGTTGATTATCATAATCAGTTAAAACTTTCAACAACGGATCTCTTGGTTCCGGCATCGATGAAGAACGCAGCGAAATGCGATAAGTAATGTGAATTGCAGAATTCAGTGAATCATCGAGTCTTTGAACGCACATTGCGCCCCCTGGTATTCCGGGGGGCATGCCTGTCCGAGCGTCATTGCTGCCCTCAAGCACGGCTTGTGTGTTGGGCCGCCGTCCCCGGTTTCCCCCGGGGACGGGCCCGAAAGGCAGCGGCGGCACCGCGTCCGGTCCTCGAGCGTATGGGGCTTTGTCACCCGCTCTGTAGGCCTGGCCGGCGCCAGCCGACACCCAACTTTATTTCTAAGGTTGACCTCGGATCAGGTAGGGATACCCGCTGAACTTAAG | > Aspergillus pseudoviridinutans strain LSPQ-01141 beta-tubulin (BenA) gene,  TATGTCTCGACGTCAATGCTTGGATGATGGGAGATTAAGACCTGTCATCTTGGCAGGCTGTCCTCCATGGATTCAGCTTCGCTGTCATGGGTATCAGCTAACAGATTTACAGGCAGACCATCTCTGGTGAGCACGGCCTTGACGGCTCTGGCCAGTAAGTTCGACCTTTATCCTCCCAATTGAGAAAGCGGGGGAAACACGAAAGGCAAGCAGGAAGAGGACGCGTGTCTGATCTGGGATAATAGTTACAATGGCTCCTCCGATCTCCAGCTGGAGCGCATGAACGTCTATTTCAACGAGGTGCGTGGATGGAACTCTCGACTCTACATTACTTCGGCAACATCTCACGATCTGACTCGCTACTAGGCCAGCGGTGACAAGTATGTTCCTCGTGCCGTTCTGGTCGATCTCGAGCCCGGTACCATGGACGCTGTCCGTGCCGGTCCTTTCGGCGAG | > Aspergillus pseudoviridinutans strain LSPQ-01141 calmodulin gene,  CCGAGTACAAGGAGGCCTTCTCTCTCTTCGTAAGTGAATTGTTTGGCCCCTAGTCGTTGCATAAGAGGAATTTCCAAAATATTGAGAGTGTGCGCTCACGCGAGATTCGGCCTATAGGACAAGGATGGTGATGGTTAGTGACCTTTTTTCCGCTCCTCGAACGTCGGATTCCTCGCGATCATGTTCAAATGCCAACTTTCAATATCCGGAAATGACCCATCAATACTGATAATATCTATGTTTGACTATTAGGCCAGATCACCACCAAGGAACTGGGCACTGTGATGCGCTCCCTGGGCCAGAACCCTTCCGAGTCAGAGCTGCAAGATATGATCAACGAGGTGGATGCTGACAACAATGGCACCATCGATTTCCCTGGTATGCGGATGCATCTAGTGTGACCCTAGGAGGGAGGAGAACAATCATTAACTTGTATTCAGAATTTCTTACTATGATGGCTCGGAAGATGAAGGACACCGACTCCGAAGAGGAAATCCGGGAAGCCTTCAAGGTCTTCGACCGTGACAACAACGGTTTTATTTCCGCTGCGGAGCTGCGCCACGTTATGACCTCTATCGG |
| Aspergillus vadensis | > Aspergillus vadensis strain CBS 113365 ITS  GCGGGTCCTTTGGGCCAACCTCCATCCGTGTCTATTATACCCTGTTGCTTCGGCGGGCCCGCCGCTTGTCGGCCGCCGGGGGGGCGCCTTTGCCCCCCGGGCCCGTGCCCGCCGGAGACCCCAACACGAACACTGTCTGAAAGCGTGCAGTCTGAGTTGATTGAATGCAATCAGTTAAAACTTTCAACAATGGATCTCTTGGTTCCGGCATCGATGAAGAACGCAGCGAAATGCGATAACTAATGTGAATTGCAGAATTCAGTGAATCATCGAGTCTTTGAACGCACATTGCGCCCCCTGGTATTCCGGGGGGCATGCCTGTCCGAGCGTCATTGCTGCCCTCAAGCCCGGCTTGTGTGTTGGGTCGCCGTCCCCCTCTCCGGGGGGACGGGCCCGAAAGGCAGCGGCGGMACCGYGTCCGATCCTCGAGCGTATGGGGCTTTGTCACATGCTCTGTAWGATTGGCCCGGCGCCTGCCGACGT | > Aspergillus vadensis strain DTO 422-H3 beta-tubulin (tub2) gene,  CGGTGCTGCTTTCTGGTACGTTTTCACTACCACTGGATTGGGGATGGAACATCATCTCTCAAGCTATCTCAGCTTGAGTTCAGATGTTATCCATCGGGTATATAGCTATCGGGTTAAGAACACGTCTAACAACTCAACAGGCAGACCATCTCTGGCGAGCACGGCCTTGACGGCTCCGGTGTGTAAGTGCAACTTTTTCACACCTCTCAATTGGTCAACAATGTGGAAAGGATTGGGTTTCCTGACGCGCAAGATAGTTACAATGGCACCTCCGACCTCCAGCTGGAGCGCATGAACGTCTACTTCAACGAGGTTAGATCACACCGTCCCTGAGTTTCTTCACGACAATATCATCAATGTCCTGACCACTTCAGCAGGCTAGCGGTAACAAGTATGTCCCCCGTGCCGTCCTCGTCGATCTGGAGCCCGGTACCATGGACGCCGTCCGTGCCGGTCCCTTCGGCCAGCTTTTCCGCCCCGACAACTTCGTCTTCGGCCAGTCCGGTGCTGGTAACAACTGGGCCAAGGGTCACT | > Aspergillus vadensis strain DTO 422-H3 calmodulin (cmdA) gene,  GCCTTCTCCCTCTTTGTGAGTGCTCCCTGAATGGACCCCCGTTCCCTCAAATTGATGACCTATCTTTACCGACTCATAATGCTAATGTATCTTCGAACTCAATAGGACAAGGATGGCGATGGTGGGTGTTTCTGTCCCCTTCACGTTTTACCTGTAGCGCCCAATCCGACCGCGGGATTTCGACAGCCATTCCCCCATCGATCTGAATCATTATACTGATGTAATCGGGAAATAGGCCAGATCACCACCAAGGAGCTCGGCACTGTGATGCGCTCCCTCGGCCAGAACCCCTCTGAGTCTGAGCTTCAGGACATGATCAACGAGGTTGACGCTGACAACAACGGAACGATCGACTTCCCCGGTATGTGATAGATCTACGCCTGTAAGGCGGGAATGCCGTATGGGTTGTGATTGACTTTTGCCGCCAGAATTCCTCACCATGATGGCTCGTAAGATGAAGGACACCGACTCCGAGGAGGAAATCCGCGAGGCTTTCAAGGTCTTCGATCGCGACAACAATGGTTTCATCTCCGCCGCGGAGTTGCGCCACGT |
| Aspergillus protuberus | >Aspergillus protuberus strain DTO:268-C4 ITS TGAGTGCGGGCTGCCTCCGGGCGCCCAACCTCCCACCCGTGACTACCTAACACTGTTGCTTCGGCGGGGAGCCCTCTCGGGGGCGAGCCGCCGGGGACTACTGAACTTCATGCCTGAGAGTGATGCAGTCTGAGTCTGAATATAAAATCAGTCAAAACTTTCAACAATGGATCTCTTGGTTCCGGCATCGATGAAGAACGCAGCGAACTGCGATAAGTAATGTGAATTGCAGAATTCAGTGAATCATCGAGTCTTTGAACGCACATTGCGCCCCCTGGCATTCCGGGGGGCATGCCTGTCCGAGCGTCATTGCTGCCCATCAAGCCCGGCTTGTGTGTTGGGTCGTCGTCCCCCCCGGGGGACGGGCCCGAAAGGCAGCGGCGGCACCGTGTCCGGTCCTCGAGCGTATGGGGCTTTGTCACCCGCTCGATTTAGGGCCGGCCGGGCGCCAGCCGACGTCCAACCATTTTTCTTCA | > Aspergillus protuberus isolate NRRL 3505 beta-tubulin gene  TGGTGCGTCGAAAATTTCATCCATTTCAGATGGTATTTCTTTTCGTGCTTTTTGCTAACGACCCTATAGGCAGACCATCTCCGGCGAGCACGGCCTTGATGGCTCCGGTGTGTGAGTACAACCCGTCCAAGACTCGATCAAAACACGACAGAACACAACCCCTGATATAATGCAGTTACAATGGTACCTCCGACCTCCAGCTCGAGCGTATGAACGTCTACTTCAACGAGGCCAGCGGCAACAAGTACGTTCCCCGTGCCGTTCTCGTCGATCTCGAGCCCGGTACTATGGATGCTGTTCGTGCCGGTCCCTTCGGTCAGCTCTTCCGTCCCGACAACTTCGTCTTTGGCCAGTCCGGTGCTGGTAACAACTGG | > Aspergillus protuberus strain CMV008B2 calmodulin gene,  GCCTTCTCCCTATTTGTAAGTGCCATTGATCCCATCGGTATCAATTATGTGGCTTTTCCGACAGGGCACTAATTTATTCTGCGCTTAACAGGACAAGGATGGCGATGGTTAGTGTGCTCCCCGCGATAACTTAGTCGCATACGCCCAATGTCGACATCATAATTCTATGTAATTGGATATTGACACACCATTCAGGCCAGATCACTACCAAGGAGCTCGGCACCGTGATGCGCTCGCTCGGCCAGAACCCCTCAGAGTCTGAGCTTCAGGACATGATCAACGAAGTCGATGCTGACAACAACGGCACCATTGATTTCCCGGGTACGTGGCCTTGCAAAAATAAAAATTACCGTCAAAGCGAACAACTGTTAACTGTCAAACAGAGTTCCTTACGATGATGGCTAGAAAGATGAAGGACACCGACTCCGAGGAGGAAATTCGGGAGGCATTCAAGGTTTTCGACCGTGATAACAATGGCTTCATCTCGGCTGCCGAGCTGCGCCACGT |
| Aspergillus tamarii | >NR_135325.1 Aspergillus tamarii NRRL 20818 ITS  AAGGATCATTACCGAGTGTAGGGTTCCTAGCGAGCCCAACCTCCCACCCGTGTTTACTGTAACCTTAGTTGCTTCGGCGGGCCCGCCTTTAAGGCCGCCGGGGGGCATCAGCCCCCGGGCCCGCGCCCGCCGGAGACACCACGAACTCTGTCTGATCTAGTGAAGTCTGAGTTGATTGTATCGCAATCAGTTAAAACTTTCAACAATGGATCTCTTGGTTCCGGCATCGATGAAGAACGCAGCGAAATGCGATAACTAGTGTGAATTGCAGAATTCCGTGAATCATCGAGTCTTTGAACGCACATTGCGCCCCCTGGTATTCCGGGGGGCATGCCTGTCCGAGCGTCATTGCTGCCCATCAAGCACGGCTTGTGTGTTGGGTCGTCGTCCCCTCTTCGGGGGGGACGGGCCCCAAAGGCAGCGGCGGCACCGCGTCCGATCCTCGAGCGTATGGGGCTTTGTCACCCGCTCTGTAGGCCCGGCCGGCGCTTGCCGAACGCAAAACAACCATTCTTTCCA | >MN787910.1 Aspergillus tamarii strain DTO 390-F5 tubulin (BenA) gene, partial cds  GCTTTCTGGTATGTCTCGATGCTTTCGAGTTAATATGCTTTGGACCAAGGAACTGCACAGAAGCATGAACTCAGATGTGCCCTACTGTGTCTGCCACGTGTTTGCTAACATCTTTGCAGGCAGACCATCTCTGGCGAGCACGGCCTTGACGGCTCCGGTGTGTAAGTACAATCCGTGTACACCTCGAACGAACGACAACCAGATGGCATTGGAAGAGTTGGAATGGGTCTGACGGGAAGGATAGTTACAATGGCTCCTCCGATCTCCAGCTGGAGCGTATGAACGTCTACTTCAACGAGGTGCGTACCTCACATTTTTCAGCCTCTTTGACAACGCTTTGCAAGTCCTGACCGCTTCTCCAGGCCAGCGGAAACAAGTATGTCCCTCGTGCCGTCCTTGTCGATCTTGAGCCCGGTACCATGGACGCCGTCCGTGCCGGTCCCTTCGGTCAGCTGTTCCGTCCCGACAACTTCGTTTTCGGCCAGTCCGGTGCTGGTAACAACTGGGCCAAGG | >MN787891.1 Aspergillus tamarii strain DTO 390-F5 calmodulin (CaM) gene, partial cds  AGGCCTTCTCCCTATTCGTAAGTCATGAACGTCGTTCGCGAAAATCGGCTTTGTGAGTAGACTTTATTTGAACACAAGCTGACGGGGCTTCTCTTGGGTTTCCTATAGGACAAGGACGGTGATGGTTAGTACAGTCTCTTTCATTCCGTCTCCCTTCAAATGCGACCAGTATCTTTTAGCCGGCATAGTTTTATCCATTTTCTGTTCGATCGGCTGAAGTCTTTGGCATTGATGGATTGACTTGATATGCAGGCCAGATCACCACCAAGGAGTTGGGCACTGTCATGCGCTCTCTGGGCCAAAACCCCTCTGAGTCGGAACTCCAGGACATGATTAACGAAGTTGACGCCGATAACAATGGCACCATTGACTTTCCTGGTACGAGAGGGCTTCCGTACATTTTACAAATAAAATAGCTGTTAATGTTCAACCAGAGTTCCTCACGATGATGGCGAGAAAGATGAAGGATACCGACTCTGAGGAGGAGATCCGGGAGGCTTTCAAGGTTTTCGACCGCGATAACAACGGTTTCATCTCCGCTGCCGAATTGCGCCACGT |
| Aspergillus glaucus | > Aspergillus glaucus NRRL 116 ITS region  AAGGATCATTACCGAGTGCGGGCCCTCTGGGTCCAACCTCCCATCCGTGTCTATCTGTACCCTGTTGCTTCGGCGTGGCCACGGCCCGCCGAAGACTAACATTTGAACACTGTCTGAAGTTTGCAGTCTGAGTTTTTAGTTAAACAATAATTAAAACTTTCAACAACGGATCTCTTGGTTCCGGCATCGATGAAGAACGCAGCGAAATGCGATAATTAATGTGAATTGCAGAATTCAGTGAATCATCGAGTCTTTGAACGCACATTGCGCCCCCTGGTATTCCGGGGGGCATGCCTGTCCGAGCGTCATTGCTGCCCTCAAGCACGGCTTGTGTGTTGGGCTTCCGTCCCTGGTAACGGGGACGGGCCCAAAAGGCAGTGGCGGCACCATGTCTGGTCCTCGAGCGTATGGGGCTTTGTCACCCGCTCCCGTAGGTCCAGCTGGCAGCTAGCCTCGCAACCAATCTTTTTAACCAGGTTGACCTCGGATCAGGTAGGGATACCCGCTGAACTTAAGCATATCAATAAGCGGAGGAAAAGAAACCAACAGGGATTGCCTCAGTAACGGCGAGTGAA | >LC589346.1 Aspergillus glaucus NRRL 116 BenA gene for beta-tubulin, partial cds  TGGTATGTCTACAATATTGGGAGGGTCGGATTAAGTGATATACTAACAGTATCATAGGCAGACTATCTCCGGCGAGCACGGTCTCGACGGCTCTGGTGTGTAAGTACCATCGGGTCTTCGGGATGGACGCGTATCGGATATGGATATCTAATGGACTGCAGCTACAATGGCTCCTCTGACCTCCAGTTGGAGCGGATGAACGTCTACTTC  AACGAGGTTTGTACATTCATTTGTGTTTGTGTGGAAACAAGTCTGACAGTGACAGGCCTCCAACAACAAATATGTCCCCCGTGCCGTCCTCGTCGACCTTGAGCCCGGTACCATGGACGCCGTCCGTGCCGGTCCCTTCGGTCAGCTCTTCCGCCCCGACAACTTCGTCTTCGGTCAGTCCGGTGCTGGTAACAACTGG | >LC589321.1 Aspergillus glaucus NRRL 116 CaM gene for calmodulin, partial cds  AGAGCAAGTCTCCGAGTACAAGGAGGCTTTCTCCCTCTTCGTAAGTGCGCTGGCCATCTCGATGTGGCATTTTCCCCGTCGGGCGTCGAGGACAGTGAACTGACCACGATTTTTGCATTATAACAGGACAAAGATGGCGATGGTTAGTGATCCCGCTATACTCTAAATTTAATGCCTCTTTACCCCGCCTATCGATTGGTTCCGACCGTGATATGATATTCGCTGTTGAGATAATGCTTTTCTGGTTAACGACACAATACTGATGGATTTCCGCGATTACAGGCCAGATCACCACCAAGGAGCTGGGTACCGTCATGCGCTCGCTGGGCCAGAACCCCTCCGAGTCGGAGTTGCAGGACATGATCAACGAGGTCGACGCTGACAACAACGGCACCATCGATTTCCCTGGTATGCGATCGTCCCGACATGAAAGCCCCCGTAACAAGATAGGCGAATCTGACCATAATAGAATTCCTTACCATGATGGCACGGAAGATGAAGGACACCGACTCCGAGGAGGAGATCCGGGAAGCTTTCAAGGTCTTCGATCGCGACAACAACGGTTTTATTTCTGCCGCGGAGTTGCGCCACGTTATGACCTCCATTGGCGAGAAGCTCACCGATGACGAAGTTGACGAGATGATTCGCGAGGCTGACCAGGACGGTGACGGCCGTATTGACTGTATGTGGACCTGGTCTTTTTCCCGGTCGAATTTGAGGAAGCTAACCGATATCAGACAACGAATTC |
| Aspergillus steynii | >NR_077197.1 Aspergillus steynii CBS 112812 ITS  CTTCCGTAGGGTGAACCTGCGGAAGGATCATTACTGAGTGAGGGTTCCTTCGGGGCCCAACCTCCCACCCTTGTATACCGTACCTAGTTGCTTCGGCGGGCCCGCCGTCCACGCGGCCGCCGGGGGAGGGGCTTCCCCCCCGGGCGAGCGCCCGCCGGAGACACCAACGTGAACACTGTCTGAAGTTTTGTCGTCTGAGTCGATTGTATCGCAATCAGTTAAAACTTTCAACAATGGATCTCTTGGTTCCGGCATCGATGAAGAACGCAGCGAAATGCGATAATTAATGTGAATTGCAGAATTCAGTGAATCATCGAGTCTTTGAACGCACATTGCACCCCCTGGTATTCCGGGGGGTATGCCTGTCCGAGCGTCATTTCTGCCCTCAAGCACGGCTTGTGTGTTGGGTCCTCGTCCCCCCCGGGGACGGGCCCGAAAGGCAGCGGCGGCACCGCGTCCGGTCCTCGAGCGTATGGGGCTTTGTCACCCGCTCTTGTAGGCCCGGCCGGCGCTGGCCGACGCTGAAAAGCAACCAATTATTTTTCCAGGTTGACCTCGGATCAGGTAGGGATACCCGCTGAACTTAAGCATATCAATAAG | >OQ446673.1 Aspergillus steynii strain EXF-15582 beta-tubulin (benA) gene,  GCAACCCCGTGTTTACACCTGTCGAAATGAAACTCGAGGAGAAACGAAACAAAATACCGTGGTGGGATTGATTGTCTGATGGGATGAACAGTTACAATGGCTCCTCCGACCTTCAGCTGGAGCGCATGAACGTCTACTTCAACGAGGTTTGTCGCCCGAAAATTTCCTCCCTTCGTGTATCCGACACGCCCCGTGCATCAAGTTCTGACCCACGCTTTCTGTACCTTCTAGGCCACCGGTGGCAAGTATGTTCCCCGTGCCGTTCTCGTCGACCTTGAGCCCGGTACCATGGACGCCGTCCGTGCCGGTCCCTTCGGTCAGCTTTTCCGCCCCGACAACTTCGTCTT | > Aspergillus steynii strain DTO:245-I5 calmodulin (cmdA) gene,  CTGTTCGTAAGTGATTCATCTGCCTGAATTAATGATGGAAAGGGGGAAAAATGCTGGCATGCTCTTTTTTCCCGTCATCAGCCTTATGGGAAACTCTAAGCTCACATGAGATTTTGCGTCTGAACAGGACAAGGATGGTGATGGTTAGTGCCACTCGGTCTCCCTTTCTATTTATCAGCCTCACGCGACCGGTTATCTCCCGTCGACTTGGAATTTTTTGATTCTTTGATACTCGCCGATCGATTCACAACATGTCGCTAATTCTGACTTCCCGCTGCAGGCCAGATCACCACCAAGGAGTTGGGCACTGTTATGCGCTCGCTGGGCCAGAACCCCTCCGAGTCCGAGTTGCAGGACATGATCAACGAGGTTGACGCCGACAACAATGGCACCATTGATTTCCCCGGTATGCGATGACGGACACACCGAATTCCAGAACGGGAAATGGCCTGAAACCAGCTGCTAATTCTCCCCCGCAGAGTTCCTGACGATGATGGCCCGAAAGATGAAGGATACCGATTCTGAGGAGGAAATCCGGGAAGCTTTCAAGGTCTTCGATCGCGACAACAACGGGTTCATCTCGGCCGC |
| Aspergillus jensenii | > Aspergillus jensenii NRRL 58600 ITS region;  AAGGATCATTACCGAGTGCGGGCTGCCTCCGGGCGCCCAACCTCCCACCCGTGACTACCTAACACTGTTGCTTCGGCGGGGAGCCCTCTCGGGGGCGAGCCGCCGGGGACTACTGAACTTCATGCCTGAGAGTGATGCAGTCTGAGTCTGAATATAAAATCAGTCAAAACTTTCAACAATGGATCTCTTGGTTCCGGCATCGATGAAGAACGCAGCGAACTGCGATAAGTAATGTGAATTGCAGAATTCAGTGAATCATCGAGTCTTTGAACGCACATTGCGCCCCCTGGCATTCCGGGGGGCATGCCTGTCCGAGCGTCATTGCTGCCCATCAAGCCCGGCTTGTGTGTTGGGTCGTCGTCCCCCCCGGGGGACGGGCCCGAAAGGCAGCGGCGGCACCGTGTCCGGTCCTCGAGCGTATGGGGCTTTGTCACCCGCTCGATTAGGGCCGGCCGGGCGCCAGCCGACGTCTCCAACCATTTTCTTCA | >MK450977.1 Aspergillus jensenii strain CMV003H2 beta-tubulin gene, partial cds  TGGTGCGTCGAAAATTTCATCCATTTCAGATGGTATCTCCTTTCGTGCTTTTTGCTAACGACTCTATAGGCAGACCATCTCCGGTGAGCACGGCCTCGATGGCTCCGGTGTGTGAGTACAACCCGTCCAGGACTCGATCAAAACACGAGACAGAACACATCCCCTGATATAATGCAGTTACAATGGTACCTCCGACCTCCAGCTCGAGCGTATGAACGTCTACTTCAACGAGGCCAGCGGCAACAAGTACGTTCCTCGTGCCGTCCTCGTCGATCTCGAGCCCGGTACCATGGACGCTGTCCGTGCCGGTCCCTTCGGTCAGCTTTTCCGTCCCGACAACTTCGTCTTTGGCCAGTCCGGTGCTGGTAACAACTGGGCCAAGGGTC | >MK451435.1 Aspergillus jensenii strain CMV007A9 calmodulin gene, partial cds  GCCTTCTCCCTATTTGTAAGTGCCATTGATCCCATTGGTATCAATTAAATCGCTTTTCCGGCAGGGTACTAATTTACTCTGCGCTTAACAGGACAAGGATGGCGATGGTTAGTGTGCCCCCCGCGATAACTTAGTCGCATACGCCCAATGTCGACATCATAATTCTACGTAATTGGACATTGACACACGATTCAGGCCAGATCACTACCAAGGAGCTCGGCACCGTGATGCGCTCGCTCGGCCAGAACCCTTCAGAGTCTGAGCTTCAGGACATGATCAACGAAGTCGACGCTGACAACAATGGCACCATTGATTTCCCAGGTACGTGGCCTTGCAAGAATTAAAAATGATCGTGAAATCGAACAACTTTTAACTGTCATTTAGAGTTCCTCACGATGATGGCTAGAAAGATGAAGGACACCGACTCCGAGGAGGAAATTCGGGAGGCATTCAAGGTTTTCGACCGTGATAACAATGGCTTCATCTCGGCTGCCGAGCTGCGCCACGT |
| Aspergillus costaricensis | >NR_103604.1 Aspergillus costaricensis CBS 115574 ITS region; from TYPE material  CCGAGTGCGGGTCCTTTGGGCCCAACCTCCCATCCGTGTCTATTATACCCTGTTGCTTCGGCGGGCCCGCCGCTTGTCGGCCGCCGGGGGGGCGCCTTTGCCCCCCGGGCCCGTGCCCGCCGGAGACCCCAACACGAACACTGTCTGAAAGCGTGCAGTCTGAGTTGATTGAATGCAATCAGTTAAAACTTTCAACAATGGATCTCTTGGTTCCGGCATCGATGAAGAACGCAGCGAAATGCGATAACTAATGTGAATTGCAGAATTCAGTGAATCATCGAGTCTTTGAACGCACATTGCGCCCCCTGGTATTCCGGGGGGCATGCCTGTCCGAGCGTCATTGCTGCCCTCAAGCCCGGCTTGTGTGTTGGGTCGCCGTCCCCCTCTCCGGGGGGACGGGCCCGAAAGGCAGCGGCGGCACCGCGTCCGATCCTCGAGCGTATGGGGCTTTGTCACATGCTCTGTAGGATTGGCCGGCGCCTGCCGACGTTTTCCAACCATTTTTTCCA | >GU296699.1 Aspergillus costaricaensis strain CBS 115574 beta-tubulin (tub2) gene, partial cds  AGGCAGACCATCTCTGGCGAGCACGGCCTTGACGGCTCCGGTGTGTAAGTGCAACTTTTTCACACCTCTCAATTGGTCAACAATGTGGAAAGGATTGGGTTTCCTGACACGCAGGATAGTTACAATGGCACCTCCGACCTCCAGCTGGAGCGCATGAACGTCTACTTCAACGAGGTTAGATCACACCGTCCCTGAGTTTTTTCACGACAATATCATCAATGTCCTGACCACTTCAGCAGGCTAGCGGTAACAAGTATGTCCCCCGTGCCGTCCTCGTCGATCTCGAGCCCGGTACCATGGACGCCGTCCGTGCCGGTCCCTTCGGCCAGCTCTTCCGTCCCGACAACTTCGTCTTCGGCCAGTCCGGTGCTGGTAACAACTGG | >MH644993.1 Aspergillus costaricensis voucher IHEM 21971 calmodulin (caM) gene, exons 1 through 5 and partial cds  CAAGTTTCTGAGTACAAGGAGGCCTTCTCCCTCTTTGTGAGTGCTCCCTGAATGAACCCCCGTTCACTCAAATCGATGACCTATCTTTCCCGGCTCATAACGCTAATGTATTTTCGAACTCAATAGGACAAGGATGGCGATGGTGGGTGGAATTCTGTCCCCTTCACGTTTTACCTGTAGCGCTCGATCCGACCGCGGGATTTCGACAGCCATTCCCCCATCGATCTTAATCATTATACTGATGTAATCCGGAAATAGGCCAGATCACCACCAAGGAGCTCGGCACTGTGATGCGCTCCCTCGGCCAGAACCCCTCCGAGTCTGAGCTTCAGGACATGATCAACGAGGTTGACGCTGACAACAACGGAACGATCGACTTCCCCGGTATGTGATAGATCTACGCCTGTAAGGCGGGAATGCCGTATGGGTTGTGATTGACTTTTGCCGCCAGAATTCCTTACCATGATGGCTCGTAAGATGAAGGACACCGACTCCGAGGAGGAAATCCGCGAGGCTTTCAAGGTCTTCGACCGCGACAACAATGGTTTCATCTCCGCCGCGGAGTTGCGCCACGTCATGACCTCCATTGGCGAGAAGCTCACTGACGACGAAGTCGATGAGATGATCCGTGAGGCTGACCAGGACGGTGATGGCCGCATCGACTGTATGTTTCCCATTCTTGATATGCCCCGTCATATGACATGCTAACTCTGCTACCAGACAACGAG |
| Aspergillus sclerotiicarbonarius | >NR_166016.1 Aspergillus sclerotiicarbonarius CBS 121057 ITS region; from TYPE material  CCGAGTGAGGGTCCTTTGGGCCCAACCTCCCACCCGTGTCTATTGTACCCTGTTGCTTCGGCGGGCCCGCCGCTTGTCGGCCGCCGGGGGGGCATCTCTGCCCCTCTGGCCCGCGCCCGCCGGAGACACCAACACGAACACTGTCTGAAATCGTGAAGTCTGAGTCGATTGTTTTCAATCAGTTAAAACTTTCAACAATGGATCTCTTGGTTCCGGCATCGATGAAGAACGCAGCGAAATGCGATAACTAATGTGAATTGCAGAATTCAGTGAATCATCGAGTCTTTGAACGCACATTGCGCCCCCTGGTATTCCGGGGGGCATGCCTGTCCGAGCGTCATTGCTGCCCTCAAGCCCGGCTTGTGTGTTGGGTCGCCGTCCCCCTTTCCGGGGGGACGGGCCCGAAAGGCAGCGGCGGCACCGCGTCCGATCCTCGAGCGTATGGGGCTTTGTCACATGCTCTGTAGAATTGGCCGGCGCCTGCCGACAACTCCAACCTTTTTTTCCA | >EU159230.1 Aspergillus sclerotiicarbonarius strain CBS 121851 beta-tubulin (benA) gene, partial cds  CATCTCTGGTGAGCACGGCCTCGACGGCTCTGGTGTGTAAGTACACCCTCTTCACATCTCAATCGGTCAACCGGAGGGAAGAATCGGGTTTCCTGACGTGGAGGATAGTTACAATGGCTCCTCCGATCTCCAGCTGGAGCGCATGAACGTCTACTTCAACGAGGTGGGTGTCAACCAGCCCCCCTAGCTATACCAACGTGTACGTGTGGTGTCTCGATTCCTAACCCCTTCACCAGGCTAGCGGCAACAAGTATGTTCCTCGTGCCGTCCTTGTCGACCTTGAGCCCGGTACCATGGATGCCGTCCGTGCCGGTCCTTTCGGTCAGCTTTTCCGCCCCGACAACATCGTCTTCGGCCAGTCCGGTGCTGGTAACAACTGG | >EU159239.1 Aspergillus sclerotiicarbonarius strain CBS 121851 calmodulin (cmd) gene, partial cds  GATGGAATACCAATAGGACAAGGATGGTGATGGTGGGTGGAATTCTGTCCCCTTGATGTTTACCTATGGCGACCGAACCGACCGCGGGGTTCCGAAAGCCGTATTCCGATCGACTTGAACCTAATACTGATGTGACTTGGGAAATAGGCCAGATCACCACCAAGGAGCTCGGCACTGTCATGCGCTCGCTCGGCCAGAACCCCTCCGAGTCTGAGCTCCAGGACATGATCAATGAGGTTGACGCTGACAACAACGGCACGATCGACTTCCCTGGTATGTGGCGGCTTTATATTTATAGAAAGCGGGAATGCGGACCAGGTATTAACTTCTGCCAGAATTCCTCACTATGATGGCCCGTAAGATGAAGGACACCGACTCCGAGGAGGAGATCCGCGAGGCCTTCAAGGTCTTCGACCGCGATAACAACGGTTTCATCTCCGCCGCGGAGCT |
| Aspergillus puulaauensis | >Aspergillus puulaauensis isolate NRRL 35641 ACCCGTGACTACCTAACACTGTTGCTTCGGCGGGGAGCCCTTTCGGGGGCGAGCCGCCGGGGACTACTGAACTTCATGCCTGAGAGTGATGCAGTCTGAGTCTGAATATAAAATCAGTCAAAACTTTCAACAATGGATCTCTTGGTTCCGGCATCGATGAAGAACGCAGCGAACTGCGATAAGTAATGTGAATTGCAGAATTCAGTGAATCATCGAGTCTTTGAACGCACATTGCGCCCCCTGGCATTCCGGGGGGCATGCCTGTCCGAGCGTCATTGCTGCCCATCAAGCCCGGCTTGTGTGTTGGGTCGTCGTCCCCCCCGGGGGACGGGCCCGAAAGGCAGCGGCGGCACCGTGTCCGGTCCTCGAGCGTATGGGGCTTTGTCACCCGCTCGATTAGGGCCGGCCGGGCGCCAGCCGACGTCTCCAACCATTTTTTTCAGG | > Aspergillus puulaauensis isolate NRRL 58602 beta-tubulin gene, partial cds  TAAGTGCTAGGGAATAATGGAATTGGTTGTCTTGAGATGGGTAAAAGCATACTAAACTTTGATGCGTGACAGGGTAACCAAATTGGTGCTGCTTTCTGGTGCGTCGAAAATTTCATCCATTCCAGATGGTATTTCCTTTCGTGCTTTTTGCTAACGACTCTATAGGCAGACCATCTCCGGTGAGCACGGCCTCGATGGCTCCGGTGTGTGAGTACAACCCGTCCAGGACTCGATCAAAACACGAGACAGAACACATCCCCTGATATAATGCAGTTACAATGGTACCTCCGACCTCCAGCTCGAGCGTATGAACGTCTACTTCAACGAGGCCAGCGGCAACAAGTACGTTCCTCGTGCCGTCCTTGTCGATCTCGAGCCCGGTACCATGGACGCTGTCCGTGCCGGTCCCTTCGGTCAGCTCTTCCGTCCCGACAACTTCGTCTTTGGCCAGTCCGGTGCTGGTAACAACTGGGCCAAGGGTCACTACACTGAGGGTGCTGAGCTCGTCGACAACGTCGTCGATGTCGTCCGTCGCGAGGCTGAGGCTTGCGACTGCCTCCAGGGTTTCCAGATCACTCACTCTCTTGGTGGTGGTACCGGTGCCGGTATGGGTACCCTCCTGATCTCCAAGATCCGTGAGG | > Aspergillus puulaauensis isolate NRRL 58602 calmodulin gene, partial cds  CAGGTTTCCGAATACAAGGAGGCCTTCTCCTTATTTGTAAGTGCCATTGATTCCATTGGTATCAATTAAATAGCTTTTCCGGCAGGGTACTAATCTACTCTGCGCTTAACAGGACAAGGATGGCGATGGTTAGTGTGCTCCCCGCGATAACTTAGTCGCATACGCCCAATGTCGACATCATAATTCTATGTAATTGGACATTGACACACGATTCAGGCCAGATCACTACCAAGGAGCTCGGCACCGTGATGCGCTCGCTCGGCCAGAACCCTTCAGAGTCTGAGCTTCAGGACATGATCAACGAAGTCGACGCTGACAACAATGGCACCATTGATTTCCCAGGTACGTGGCCTTGCAAGAATTAAAAATGATCGTGAAATCGAACAACTTTTAACTGTCATTTAGAGTTCCTCACGATGATGGCTAGAAAGATGAAGGACACCGACTCCGAGGAGGAAATTCGGGAGGCATTCAAGGTTTTCGACCGTGATAACAATGGCTTCATCTCGGCTGCCGAGCTGCGCCACGTCATGACCTCCATTGGTGAGAAGCTCACTGATGATGAAGTCGACGAGATGATTCGTGAGGCGGATCAGGATGGTGATGGCCGGATTGACTGTACGTTGGCTTCCCCGATTATGATTTGACGGTCAGAAAAGATGCTAATTCTCAGCAGACAACGAATTC |
| Aspergillus minisclerotigenes | > Aspergillus minisclerotigenes isolate EV34  GGAAGTAAAAGTCGTAACAAGGTTTCCGTAGGTGAACCTGCGGAAGGATCATTACCGAGTGTAGGGTTCCTAGCGAGCCCACCTCCCACCCGTGTTTACTGTACCTTAGTTGCTTCGGCGGGCCCGCCATTCGTGGCCGCCGGGGGCTCTCAGCCCCGGGCCCGCGCCGCCGGAGACACCACGAACTCTGTCTGATCTAGTGAAGTCTGAGTTGATTGTATCGCAATCAGTTAAAACTTTCAACAATGGATCTCTTGGTTCCGGCATCGATGAAGAACGCAGCGAAATGCGATAACTAGTGTAATTGCAGAATTCCGTGAATCATCGAGTCTTTGAACGCACATTGCGCCCCCTGGTATTCCGGGGGGCATCCTGTCCGAGCGTCATTGCTGCCCATCAAGCACGGCTTGTGTGTTGGGTCGTCGTCCCCTCTCCGGGGGGGACGGGCCCCAAAGGCAGCGGCGGCACCGCGTCCGATCCTCGAGCGTATGGGGCTTTGTCACCCGCTCTGTAGGCCCGGCCGGCGCTTGCCGAACGCAAATCATCTTTTCCAGGTTGACCTCGGATCAGTAGG | > Aspergillus minisclerotigenes strain DTO 228-H5 beta-tubulin (BenA) gene, partial cds  TGGTATGTCTCAATGCCTCCGAGTTGATATGCTTTGGACCAAAGAACTCCTCAAAAGCATGATCTCGGATGTGTCCTATTATATCTGCCACGTGTTTGCTAACATCTTTGCAGGCAAACCATCTCTGGCGAGCACGGCCTTGACGGCTCCGGTGTGTAAGTACAGCCTGTATACACCTCGAATGAACGACGACCATATAGCATTAGAAGTTGGAATGGATCTGACGGCAAGGATAGTTACAATGGCTCCTCCGATCTCCAGCTGGAGCGTATGAACGTCTACTTCAACGAGGTGCGTACCTCAAAATTTTCAGCATCTATGAAAAGGCTTTGCAACTCCTGACCGCTTCTCCAGGCCAGCGGAAACAAGTATGTCCCTCGTGCCGTCCTCGTCGATCTTGAGCCTGGTACCATGGACGCC  GTCCGTGCCGGTCCCTTCGGTCAGCTCTTCCGTCCCGACAACTTCGTTTTCGGCCAGTCCGGTGCTGGTAACAACTGGGCCAAG | > Aspergillus minisclerotigenes strain DTO 228-H1 calmodulin (CaM)  TCGTAAGTAGTTATCGTCGTTCGTGAAAATTGGTTTTGTTAGTCGTCATGATTTGAACACAAGCTGACTTGGCTTTTCTTGGGTTTCCTATAGGACAAGGACGGTGATGGTTAGTACAGTTTATTTTATTCATTCTCCCTTCAAATGCGACCAATATGTTTTAGCCGCCATAATTTTATCCAGTTTCTGTTCGATCGGCTGAAGTCTTGGCATTGATGAATTGACTTGATATGCAGGCCAGATCACCACCAAGGAGCTGGGCACTGTCATGCGCTCTCTGGGCCAAAACCCCTCTGAGTCGGAACTCCAGGACATGATTAACGAGGTTGACGCCGACAACAATGGCACCATTGACTTCCCTGGTACGAGACGGCTTCTGTACGATTCATAAATGAAATAGCTGTTAATGTTCAAATAGAGTTCCTTACGATGATGGCGAGAAAGATGAAGGATACCGACTCTGAGGAGGAGATCCGGGAGGCTTTCAAGGTTTTCGACCGCGATAACAACGGCTTCATCTCCGCTGCCGAA |
| Aspergillus fijiensis | > Aspergillus fijiensis strain CBS 119.49 ITS  ACAAGGTTTCCGTAGGTGAACCTGCGGAAGGATCATTACCGAGTGCTGGGTCCTTCGGGGCCCAACCTCCCACCCGTGCTTACCGTACCCTGTTGCTTCGGCGGGCCCGCCTTCGGGCGGCCCGGGGCCTGCCCCCGGGACCGCGCCCGCCGGAGACCCCAATGGAACACTGTCTGAAAGCGTGCAGTCTGAGTCGATTGATACCAATCAGTCAAAACTTTCAACAATGGATCTCTTGGTTCCGGCATCGATGAAGAACGCAGCGAAATGCGATAACTAATGTGAATTGCAGAATTCAGTGAATCATCGAGTCTTTGAACGCACATTGCGCCCCCTGGTATTCCGGGGGGCATGCCTGTCCGAGCGTCATTTCTCCCCTCCAGCCCCGCTGGTTGTTGGGCCGCGCCCCCCCGGGGGCGGGCCTCGAGAGAAACGGCGGCACCGTCCGGTCCTCGAGCGTATGGGGCTCTGTCACCCGCTCTATGGGCCC  GGCCGGGGCTTGCCTCGACCCCCAATCTTCTCAGATTGACCTCGGATCAGGTAGGGATACCCGCTGAACTTAAGCATATCAA | > Aspergillus fijiensis partial tubb gene for beta tubulin, strain ITEM 14784  GGTGCTGCTTTCTGGTATGTCTCTACTCGAGTGAATGGATAGATGACATGTTCTGTAGACTCTCACCCAGAGTCAAGAACATTACTGTCAAAGCTTCTCTGTGGTCAATTGAGCTAACAATCTTCCAGGCAGACCATCTCTGGTGAGCACGGCCTCGATGGCGCCGGTGTGTAAGTGCAGACTTCCCGGGCGGCAGGACCAGCGGGTCGCGGAAAATGGTGAAGGATATTAATGGAAAGGACAGTTACAATGGCTCCTCCGACCTCCAGCTGGAGCGCATGAACGTCTACTTCAACGAGGTTCGTCTTCGAGTCCTCCCTCCGGCTTTCCCGCATCAGCTCCTGACTGCTTCACCAGGCTAGCGGCAACAAGTATGTTCCCCGTGCCGTCCTCGTCGACCTCGAGCCCGGTACCATGGACGCCGTCCGTGCCGGTCCTTTCGGCCAGCTTTTCCGCCCCGACAACTTCGTCTTCGGTCAATCCGGTGCTGGTAACAACTGGGCCAAGGGTCACTACACTGAGGGT | >HE984428.1 Aspergillus fijiensis partial caM gene for calmodulin, exons 1-5  CAAGGAGGCCTTCTCCCTTTTTGTAAGCGACCCCGATGCTTTCTCGTCTGCTATAAAACAATGTTTACTGATTCGTTTCTCGTGATCCCTGATAGGACAAGGATGGCGATGGTTAGTGTAGTTCTTTACCCCTCAACGAAACCCGCCTGGCATCACCTGGCACTATAGCCACCATACCGCAAACGCCACAGAAACTATTAAGAATCGATCGCATTTCTGACAGAATCCATGCTACAGGACAAATTACCACCAAGGAGTTGGGTACCGTTATGCGCTCTCTCGGCCAGAACCCCTCCGAGTCTGAGCTCCAGGACATGATCAACGAGGTTGACGCTGACAACAACGGCACTATCGACTTTCCCGGTATGTGATCGCCATGCATCGAGCTCCGGACATAGTATTGACAGCTGCCCAGAATTCCTTACCATGATGGCCCGTAAGATGAAGGATACCGACTCTGAGGAGGAGATCCGTGAGGCCTTCAAGGTCTTCGATCGCGATAACAACGGCTTCATCTCTGCCGCGGAGCTGCGCCACGTCATGACCTCTATCGGCGAGAAGCTCACCGATGACGAAGTCGATGAGATGATCCGTGAGGCGGACCAGGATGGTGACGGCCGTATTGACTGTATGTTTCACACATTCCCTGCTTGAGTGTCCGCCCGATGACTAATTTTCGCTCCTCCCTCCAGACAATGAGTTTGTCCAACTCATGATGCAAA |
| Aspergillus felis | >Aspergillus felis CBS 130245 ITS region;  GATGGCTCGGTGAGGCCTTCGGACTGGCTCAGGGGAGTTGGCAACGACTCCCCAGAGCCGGAAAGTTGGTCAAACCCGGTCATTTAGAGGAAGTAAAAGTCGTAACAAGGTTTCCGTAGGTGAACCTGCGGAAGGATCATTACCGAGTGAGGGCCCTCTGGGTCCAACCTCCCACCCGTGTCTATTGTACCTTGTTGCTTCGGCGGGCCCGCCGTTTCGACGGCCGCCGGGGAGGCCTCGCGCCCCCGGGCCCGCGCCCGCCGAAGACCCCAACATGAACGCTGTTCTGAAAGTATGCAGTCTGAGTTGATTATCATAATCAGTTAAAACTTTCAACAACGGATCTCTTGGTTCCGGCATCGATGAAGAACGCAGCGAAATGCGATAAATAATGTGAATTGCAGAATTCAGTGAATCATCGAGTCTTTGAACGCACATTGCGCCCCCTGGTATTCCGGGGGGCATGCCTGTCCGAGCGTCATTGCTGCCCTCAAGCACGGCTTGTGTGTTGGGCCGCCGTCCCCGGTTTCCCCCGGGGACGGGCCCGAAAGGCAGCGGCGGCACCGCGTCCGGTCCTCGAGCGTATGGGGCTTTGTCACCCGCTCTGTAGGCCCGGCCGGCGCCAGCCGACACCCAACTTTATTTCTAAGGTTGACCTCGGATCAGGTAGGGATACCCGCTGAACTTAAGCATATCAATAAGCGGAGGAAAAGAAACCAACAGGGATTGCCTCAGTAACGGCGAGTGAA | >MN969363.1 Aspergillus felis strain CBS 130245 beta-tubulin (BenA) gene, partial cds  GGTGCTGCTTTCTGGTATGTCTCGACCTCAATGCTTTGATGATGGGAGATTTAGGACCTGTGATCTTAGCAGGCTGTCCTCCATGGATTTAGTTTCGCTGTCATGGGTATCAGCTAACAGATTTACAGGCAGACCATCTCTGGTGAGCACGGCCTCGACGGCTCTGGCCAGTAAGTTCGACCTTTATCCTCCCAATTGAGAAAGCAACGAAAGGCAAGCAGGAAGAGGACGCGTATCTGATCCGATATAATAGCTACAATGGCTCCTCCGATCTCCAGCTGGAGCGCATGAACGTCTACTTCAACGAGGTGCGTGGATGAAACTCTCGACTCTACATTAGTTCGGCAACATCTCACGATCTGACTCGCTACTAGGCCAACGGTGACAAGTATGTTCCTCGTGCCGTTCTGGTCGATCTCGAGCCCGGTACCATGGACGCTGTCCGTGCCGGTCCTTTCGGCGAGCTCTTCCGTCCCGACAACTTCGTCTTCGGCCAGTCTGGTGCTGGTAACAACTGGGCCAAGGGTCAC | >KJ914707.1 Aspergillus felis isolate CBS130246 calmodulin gene, partial cds  CAGGTTTCCGAGTACAAGGAGGCTTTCTCTCTCTTCGTAAGTGAATTGTTTAAGCCCCTAGTCGTTGCATAAGAGAAATTTCCACAATATTGAGAGTGTGCGCTCACGCGAGATTCGACCTATAGGATAAGGATGGTGATGGTTAGTGACCTTTTTTCCGCTCCTCGAACGTCGGATTCCTCGCGATCATGTTCAAATGCCGACTTGCAATATCCGGAAATGACCCATCAATACTGATAATATCTATGTTTGACTATTAGGCCAGATCACCACCAAGGAACTGGGCACTGTGATGCGCTCCCTGGGCCAGAACCCTTCCGAGTCAGAGCTGCAAGATATGATCAACGAGGTGGATGCTGACAACAATGGCACCATCGATTTCCCTGGTATGCGGATGCATCTAGCGTGACCCCGGGAGGGAGGAGAACAATCATTAACTTGTATTCAGAATTCCTTACTATGATGGCTCGGAAGATGAAGGACACCGACTCCGAAGAGGAAATCCGGGAAGCTTTCAAGGTCTTCGACCGCGACAACAACGGTTTCATTTCCGCTGCGGAGCTGCGCCACGTTATGACCTCTATCGGAGAGAAGCTCACTGATGACGAAGTTGACGAGATGATCCGCGAGGCGGATCAGGACGGTGACGGCCGGATTGATTGTATGTTGATCAAACAGCTTTCCTTTGTCGGACCCCCATTTACATGGGATATTTTGCTAATTGTTTGTAGACAACGAGTTC |
| Aspergillus aculeatus | >Aspergillus aculeatus CBS 172.66 ITS region  CCGAGTGCTGGGTCCTTCGGGGCCCAACCTCCCACCCGTGCTTACCGTACCCTGTTGCTTCGGCGGGCCCGCCTTCGGGCGGCCCGGGGCCTGCCCCCGGGACCGCGCCCGCCGGAGACCCCAATGGAACACTGTCTGAAAGCGTGCAGTCTGAGTTGATTGATACCAATCAGTTAAAACTTTCAACAATGGATCTCTTGGTTCCGGCATCGATGAAGAACGCAGCGAAATGCGATAACTAATGTGAATTGCAGAATTCAGTGAATCATCGAGTCTTTGAACGCACATTGCGCCCCCTGGTATTCCGGGGGGCATGCCTGTCCGAGCGTCATTTCTCCCCTCCAGCCCCGCTGGTTGTTGGGCCGCGCCCCCCCGGGGGCGGGCCTCGAGAGAAACGGCGGCACCGTCCGGTCCTCGAGCGTATGGGGCTCTGTCACCCGCTCTATGGGCCCGGCCGGGGCTTGCCTCGACCCCCAATCTTCTCA | >Aspergillus aculeatus isolate CCMFBH-993 beta-tubulin (benA) gene,  TGGTAACCAAAATCGGTGCTGCTTTCTGGTACGCTCCAGCCTTTATCACCTCTCCATCCCCGTCATGGCTAACCCCCCACCAGGCAGACCATCTCCGGCGAACATGGCCTCGACGGCGACGGCGTGTCAGTGACCCAGCTGGCCCTCGACTCCTTCCCCTTCTGACATCCATCCTAGATACAATGGCAGCTCCGACCTCCAGCTGGAGCGCATGAACGTCTACTTCAACGAAGCCGGCAACAACAAGTACGTGCCCCGCGCGGTGCTGGTTGATCTGGAGCCCGGTACGATGGACGCTCTGCGCTCCGGCCCCATGGGCGGCCTCTTCCGCCCCGACAACTACGTGTTCGGCCAGTCCGGTGCGGGCAACAACTGGGCCAAGGGTCACTAC | > Aspergillus aculeatus partial caM gene for caolmodulin,  GGAGGCCTTCTCCCTCTTTGTAAGCGACCTCGATGCTTTCTCGTCTGCTATGAAACAATGTTTACTGATCCGTTACTCGTGATCCCTGATAGGACAAGGATGGCGATGGTTAGTGTAGTTCCTTACCCCTCAACGAAACCCGCCTGGCACTACTATAGCCACCAACCGCAAACGTCACAGAAACTACTAAGAATCGATCGCATTTCTGACAGAATCCATGCTACAGGACAAATTACCACCAAGGAGTTGGGTACCGTTATGCGCTCTCTCGGCCAGAACCCCTCCGAGTCTGAGCTCCAGGACATGATCAACGAGGTTGATGCTGACAACAATGGCACTATCGACTTCCCCGGTATGTGATGGCCATGCATTGGGCTCCGGACATAGTATTGACAGGCTGCCCAGAATTCCTTACCATGATGGCCCGTAAGATGAAGGATACCGACTCCGAGGAGGAGATTCGTGAGGCCTTCAAGGTCTTCGATCGCGATAACAACGGCTTCATCTCTGCCGCGGAGCTGCGTCACGTCATGACTTCTATCGGCGAGAAGCTCACCGATGACGAAGTCGATGAGATGATCCGTGAGGCGGACCAGGATGGTGACGGCCGTATTGACTGTATGTTTCACACATTCCCTGCTTGAGTGTCCGTCCGATGACTAATTTTGCTCCTTCCTCCAGACAATGAGTTTGTCCAACTCATGATGC |
| Aspergillus homomorphus | >NR_077189.1 Aspergillus homomorphus CBS 101889 ITS region; from TYPE material  CCGAGTGCTGGGTCCTTCGGGGCCCAACCTCCCACCCGTGCTTACCGTACCCTGTTGCTTCGGCGGGCCCGCCATTGGCGGCCCGGGGCCTGCCCCCGGGATCGCGCCCGCCGGAGACCCCAACACGAACACTGTCTGAAAGCGTGCAGTCTGAGTCGATTGATACCAATCAGTTAAAACTTTCAACAATGGATCTCTTGGTTCCGGCATCGATGAAGAACGCAGCGAAATGCGATAACTAATGTGAATTGCAGAATTCAGTGAATCATCGAGTCTTTGAACGCACATTGCGCCCCCTGGTATTCCGGGGGGCATGCCTGTCCGAGCGTCATTTCTCCCCTCAAGCCCGGCTTGTTATTGGGTCGAGTCCCCCCGGGGGCTGGCCCGTAAAGAAATGGCGGCACCGTCCGGTCCTCGAGCGTATGGGGCTCTGTCACCCGCTCTAAGGGCCCGGTCGGGGCTGTAGCCTCACCCCCAATCTTTTAA | >LR738704.1 Aspergillus homomorphus partial tubb gene for beta tubulin, strain A055  TATCTCTGGCGAGCACGGCCTTGATGGCTCCGGTGTGTAAGTACCGACTTTCCAGGCCGCGGGTCAAACAGATACACGGAAAAGGGTGAAGAATCCTAACCCAAGGACAGTTACAATGGCTCCTCCGATCTCCAGCTGGAGCGCATGAACGTCTACTTCAACGAGGTTCGTTTCGATCGAGTCTTTATGTCTCCGGCTTCCCGCAGCAGC  TCCTGACTCCTTCACCAGGCTAGCGGCAACAAGTATGTTCCTCGTGCCGTCCTCGTCGATCTCGAGCCCGGTACCATGGACGCCGTCCGTGCCGGTCCTTTCGGCCAGCTTTTCCGCCCCGACAACTTCGTCTTC | >AM887865.1 Aspergillus homomorphus partial cam gene for calmodulin, strain ITEM 7556, exons 2-3  GAATGTTTACTGATGCGGTTCTCGTGATCCTCGAACAGGACAAGGATGGCGATGGTTAGTGCAGTTCCTTACCCCTCTCTCAACGATCCCACCCGGCGCCACCACAATAGCCATTATGCTGCGATTAACGGAGGACTCACACGGACACCGAACGCGCTTCTAACAGAATCCCCTTGACAGGACAAATCACCACCAAGGAGTTGGGTACCGTCATGCGTTCCCTCGGCCAGAACCCTTCCGAGTCTGAGCTTCAGGACATGATCAACGAGGTCGACGCTGACAACAACGGCACTATCGACTTCCCCGGTATGTGACGGCGATGAAGTAGGGGTTGAACATGTTGTTGACAGCTGCCAGAATTCCTTACCATGATGGCTCGTAAGATGAAGGACACCGATTCCGAGGAGGAGATCCGCGAGGCATTCAAGGTCTTCGATCGCGATAACAACGGCTTTATCTCCGCCGCGGAGCTGCGCCATGTCATGACCTCCATCGGTGAGAAGCTCACCGATGACGAGGTCGATGAGATGATCCGTGAGGCGGACCAGGACGGTGACGGCCGCATCGACTGTATGT |
| Aspergillus quadrilineatus | > Aspergillus quadrilineatus strain CBS 119.55 ITS  GGCGCCACCTCCCACCCGTGACTACCTAACACTGTTGCTTCGGCGGGGAGCCCCCTAGGGGCGAGCCGCCGGGGACCACTGAACTTCATGCCTAAGAGTGATGCAGTCTGAGCCTGAATACAAATCAGTCAAAACTTTCAACAATGGATCTCTTGGTTCCGGCATCGATGAAGAACGCAGCGAACTGCGATAAGTAATGTGAATTGCAGAATTCAGTGAATCATCGAGTCTTTGAACGCACATTGCGCCCCCTGGCATTCCGGGGGGCATGCCTGTCCGAGCGTCATTGCTGCCCTCAAGCCCGGCTTGTGTGTTGGGTCGTCGTCCCCCCCGGGGGACGGGCCCGAAAGGCAGCGGCGGCACCGTGTCCGGTCCTCGAGCGTATGGGGCTTTGTCACCCGCTCGATTAGGGCCGGCCGGGCGCCAGCCGGCGTCTCCAACCTTATTTTTCTCAGGTTGACC | >EF652317.1 Aspergillus quadrilineatus isolate NRRL 4992 beta-tubulin gene, partial cds  TGGTGAGTCGAAAATTTCACCTTTCTGGAATGATTTCTTTCTTCGTACACCTGCTAACAAGCCAACAGGCAGACCATCTCCGGTGAGCACGGCCTCGATGGCTCCGGTGTGTGAGTACAATCCGTCCAGCATCCGATGAAAACACGACACCACAACAGAACCTGACACAACCCAGTTACAATGGTACCTCCGACCTTCAGCTCGAGCGTATGAACGTCTACTTCAATGAGGTCAGTGGATGAGATATCAGGTAGCAGTTTGGTGTGTCTGACTTCGTCCAGGCCAGCGGTAACAAGTACGTTCCCCGTGCCGTCCTCGTCGATCTCGAGCCCGGTACTATGGATGCCGTCCGCGCCGGTCCCTTTGGCGAGCTCTTCCGTCCCGACAACTTCGTTTTCGGCCAGTCCGGTGCTGGTAACAACTGG | >EF652405.1 Aspergillus quadrilineatus isolate NRRL 4992 calmodulin  GTCTCCGAGTACAAGGAGGCCTTCTCCCTGTTTGTAAGTGCCATTGGTTACTGTTATTTCAAATTCGAATTTATAATGAGAGTATACTAATATATCCCGCACTTAACAGGACAAGGATGGCGATGGTTAGTGCATTTGTCCCCCCAGACCTGATCGCATTCGCCCAGCGTGTCTGCTGTAGCTCTATATAAACCGATTCTGATAAACGGCGACAGGCCAGATTACCACTAAGGAGCTTGGCACTGTCATGCGCTCGCTCGGTCAGAATCCTTCAGAGTCTGAGCTTCAGGACATGATCAACGAAGTGGACGCCGACAACAATGGCACCATTGACTTTCCAGGTACGCGAACTCCCAGTCTACTTCGCACCAAGCTAGAAACTGTACTAATGCTAAACAGAGTTCCTCACCATGATGGCCAGAAAGATGAAGGACACCGATTCCGAGGAGGAAATTCGGGAGGCGTTCAAGGTCTTCGACCGTGACAACAATGGTTTCATCTCCGCTGCTGAGCTGCGTCACGTCATGACCTCTATTGGTGAGAAGCTCACCGATGACGAAGTCGACGAGATGATCCGCGAGGCGGACCAGGATGGTGACGGCCGAATTGACTGTACGTTGGCTCCCCGCTTATCCTTGACCGTAGAAGAGGCATGATACTGATCGGCTGCAGACAACGAATTC |
| Aspergillus viridinutans | > Aspergillus viridinutans NRRL 4365 ITS region;  AAGGATCATTACCGAGTGAGGGCCCTCTGGGTCCAACCTCCCACCCGTGTCTATCGTACCTTGTTGCTTCGGCGGGCCCGCCGTTTCGACGGCCGCCAGGGGAGGCCTCGCGCCCCCGGGCCCGCGCCCGCCGAAGACCCCAACATGAACGCTGTTCTGAAAGTATGCAGTCTGAGTTGATTATCATAATCAGTTAAAACTTTCAACAACGGATCTCTTGGTTCCGGCATCGATGAAGAACGCAGCGAAATGCGATAAGTAATGTGAATTGCAGAATTCAGTGAATCATCGAGTCTTTGAACGCACATTGCGCCCCCTGGTATTCCGGGGGGCATGCCTGTCCGAGCGTCATTGCTGCCCTCAAGCACGGCTTGTGTGTTGGGCCCCCGTCCCCGGTTACCCCCGGGGACGGGCCCGAAAGGCAGCGGCGGCACCGCGTCCGGTCCTCGAGCGTATGGGGCTTTGTCACCCGCTCTGTAGGCCCGGCCGGCGCCAGCCGACACCCAACTTTATTTCTAA | > Aspergillus viridinutans strain NIHAV1 beta tubulin (benA) gene, partial sequence  GATGATGGGAGATTAGGACTTGTCATCTTAGCAGGCTGTCCTCCATGGATTTAGCTTCGCTGTCATGGGTATCAGCTAACAGATTTACAGGCAGACCATCTCTGGTGAGCACGGCCTTGACGGCTCTGGCCAGTAAGTTCGACCTTTATCCTCCCAATTGAGAAAGCGGGGGAAACACGAAAGGCAAGCAGGAAGAGGACGCGTGTCTGATCTGGGATAATAGCTACAATGGCTCCTCCGATCTCCAGCTGGAGCGCATGAACGTCTATTTCAACGAGGTGTGTGGATGGAACTCTCGACTCTACATTACTTCGGCAACATCTCACGATCTGACTCGCTACTAGGCCAACGGTGACAAGTATGTTCCTCGTGCCGTTCTGGTCGATCTCGAGCCCGGTACCATGGACGCTGTCCGTGCCGGTCCTTTCGGCGAG | >GQ144443.1 Aspergillus viridinutans strain NIHAV2 calmodulin gene, partial sequence  TCTCTCTTCGTAAGTGAATTGTTTGGCCCCTAGTCGTTGCATAAGAGGAATTTCCAAAATATTGAGAGTGTGCGCTCACGCGAGATTCGGCCTATAGGACAAGGATGGTGATGGTTAGTGACCTTTTTTCCGCTCCTCGAACGTCGGATTCCTCGCGATCATGTTCAAATGCCAACTTTCAATATCCGGAAATGACCCATCAATACTGATAATATCTATGTTTGACTATTAGGCCAGATCACCACCAAGGAACTGGGCACTGTGATGCGCTCCCTGGGCCAGAACCCTTCCGAGTCAGAGCTGCAAGATATGATCAACGAGGTGGATGCTGACAACAATGGCACCATCGATTTCCCTGGTATGCGGATGCATCTAGTGTGACCCTAGGAGGGAGGAGAACAATCATTAACTTGTATTCAGAATTTCTTACTATGATGGCTCGGAAGATGAAGGACACCGACTCCGAAGAGGAAATCCGGGAAGCCTTCAAGGTCTTCGACCGTGACAACAACGGTTTTAT |
| Aspergillus chevalieri | > Aspergillus chevalieri strain CBS 129311 ITS  TAACAAGGTTTCCGTAGGTGAACCTGCGGAAGGATCATTACCGAGTGCGGGCCCTCTGGGTCCAACCTCCCATCCGTGTCTATCTGTACCCTGTTGCTTCGGCGTGGCCACGGCCCGCCGGAGACTAACATTTGAACGCTGTCTGAAGTTTGCAGTCTGAGTTTTTAGTTAAACAATCGTTAAAACTTTCAACAACGGATCTCTTGGTTCCGGCATCGATGAAGAACGCAGCGAAATGCGATAATTAATGTGAATTGCAGAATTCAGTGAATCATCGAGTCTTTGAACGCACATTGCGCCCCCTGGTATTCCGGGGGGCATGCCTGTCCGAGCGTCATTGCTGCCCTCAAGCACGGCTTGTGTGTTGGGCTTCCGTCCCTGGCAACGGGGACGGGCCCAAAAGGCAGTGGCGGCACCATGTCTGGTCCTCGAGCGTATGGGGCTTTGTCACCCGCTCCCGTAGGTCCAGCTGGCAGCTAGCCTCGCAACCAATCTTTTTAACCAGGTTGACCTCGGATCAGGTAGGGATACCCGCTGAACTTAAGCATATCAA | >MZ826413.1 Aspergillus chevalieri isolate N24 beta-tubulin gene, partial cds  TGGTAACCAAATCGGTGCTGCTTTCTGGTATGTCCGCATTATAATCAGAGTCAGATTGGGTGATATACTAACAGTATCACAGGCAGACTATCTCCGGCGAGCACGGTCTCGACGGCTCTGGTGTGTAAGTACAGTCGGGTCTCCGAGATGGACGCGTATCGGATATGGATATCTAAATGGATTGCAGCTACAATGGCTCCTCCGACCTCCAGTTGGAGCGTATGAACGTCTACTTCAACGAGGTTTGCCTAATTCATTCGTGTCTGTGTGGGAAACAGTTCTGACAGTGACAGGCCTCCAACAACAAATATGTCCCCCGTGCCGTCCTCGTCGACCTTGAGCCCGGTACCATGGACGCCGTCCGTGCCGGTCCCTTCGGCCAGCTCTTCCGTCCCGATAACTTCGTTTTCGGTCAGTCCG  GTGCCGGTAACAACTGGGCCAAGGGTCACTACACTGAGGGTA | > Aspergillus chevalieri strain Dryhams/Hr/2021/12 calmodulin gene, partial cds  TTCTACAGGACAAGGATGGCGATGGTTAGTGATCCCGCTCTACTCTGCATCAAATGCCTCTTTTACTCCACCATTCGATTGGTTCCGACCGTGATATCATATTCGCTGTTGAAATAATATCTTTCCGGTTAACGACATAATACTGATGGATCTCTGCGATTACAGGCCAGATCACCACCAAGGAGCTGGGTACCGTTATGCGCTCGCTGGGCCAGAACCCCTCCGAGTCGGAGTTGCAGGACATGATCAACGAGGTTGACGCTGACAACAACGGCACCATTGATTTCCCTGGTATGCGATCGTCCCGACATGAAAGGTCCCGTAACAAGATAGGCGATTCTGACCATACTAGAATTCCTTACCATGATGGCA |
| Aspergillus sydowii | >NR_131259.1 Aspergillus sydowii CBS 593.65 ITS region; from TYPE material  AGGATCATTACTGAGTGCGGGCTGCCTCCGGGCGCCCAACCTCCCACCCGTGAATACCTAACACTGTTGCTTCGGCGGGGAACCCCCTCGGGGGCGAGCCGCCGGGGACTACTGAACTTCATGCCTGAGAGTGATGCAGTCTGAGTCTGAATATAAAATCAGTCAAAACTTTCAACAATGGATCTCTTGGTTCCGGCATCGATGAAGAACGCAGCGAACTGCGATAAGTAATGTGAATTGCAGAATTCAGTGAATCATCGAGTCTTTGAACGCACATTGCGCCCCCTGGCATTCCGGGGGGCATGCCTGTCCGAGCGTCATTGCTGCCCATCAAGCCCGGCTTGTGTGTTGGGTCGTCGTCCCCCCCGGGGGACGGGCCCGAAAGGCAGCGGCGGCACCGTGTCCGGTCCTCGAGCGTATGGGGCTTTGTCACCCGCTCGACTAGGGCCGGCCGGGCGCCAGCCGACGTCTCCAACCATTTTTCTTCAGG  TTGACCTCGGATCAGGTAGG | >MT410097.1 Aspergillus sydowii isolate CCMFBH-990 beta-tubulin (benA) gene, partial cds  TGGTAACCAAATCGGTGCTGCTTTCTGGTGCGTCGAAAATTTCATCCATTGCAGATGGTCTTTGTTTTCGTGATTTTTACTAACGACTCTATAGGCAGACCATCTCCGGTGAGCACGGCCTCGATGGCTCCGGTGTGTGAGTACAGCCCGTCCAGGACTCGATCAAAACACGACAGAACACAGCCCCTGATATAATACAGTTACAATGGTACCTCCGACCTCCAGCTCGAGCGTATGAACGTCTACTTCAACGAGGCCAGCGGCAACAAGTACGTTCCCCGTGCCGTCCTCGTCGATCTCGAGCCTGGTACTATGGACGCTGTCCGTGCCGGTCCCTTCGGTCAGCTCTTCCGTCCCGACAACTTCGTCTTTGGCCAGTCCGGTGCTGGTAACAACTGGGCCAAGGGTCA | > Aspergillus sydowii isolate CGMCC_3.07939 calmodulin (CaM)  GCCTTCTCCCTATTTGTAAGTGCCATTGATTCCATCGGTATCAATTGGATGGCTTTTCCGGGAGGGTACTAATCTATTCTGCACTTAACAGGACAAGGATGGCGATGGTTAGTGTGCTCCCCGCGATAACTTGGTCGCATACGCCCAATGTCGACACCATAATTCTATGTAATTGGATATTGACACACGATCCAGGCCAGATCACTACCAAGGAGCTCGGCACCGTGATGCGCTCGCTCGGCCAGAACCCCTCAGAGTCTGAGCTTCAGGACATGATCAACGAAGTCGACGCTGACAACAACGGCACCATTGATTTCCCAGGTACGTGCCTTTGCAAAAATAAGATCACCGTGAAATCGGACAACTGTTGACTGTTAAATAGAGTTCCTCACGATGATGGCTAGAAAGATGAAGGACACCGACTCCGAGGAGGAAATTCGGGAGGCATTCAAGGTTTTCGACCGTGATAACAATGGCTTCATCTCGGCTGCCGAGCTGCGCCACGT |
| Aspergillus ruber | > Aspergillus ruber NRRL 52 ITS region AAGGATCATTACCGAGTGCGGGCCCTCTGGGTCCAACCTCCCATCCGTGTCTATCTGTACCCTGTTGCTTCGGCGTGGCTACGGCCCGCCGAAGACTAACATTTGAACACTGTCTGAAGTTTGCAGTCTGAGTTTTTAGTTAAACAATAATTAAAACTTTCAACAACGGATCTCTTGGTTCCGGCATCGATGAAGAACGCAGCGAAATGCGATAATTAATGTGAATTGCAGAATTCAGTGAATCATCGAGTCTTTGAACGCACATTGCGCCCCCTGGTATTCCGGGGGGCATGCCTGTCCGAGCGTCATTGCTGCCCTCAAGCACGGCTTGTGTGTTGGGCTTCCGTCCCTGGTAACGGGGACGGGCCCAAAAGGCAGTGGCGGCACCATGTCTGGTCCTCGAGCGTATGGGGCTTTGTCACCCGCTCCCGTAGGTCCAGCTGGCAGCTAGCCTCGCAACCAATCTTTTTAACCA | >MZ595219.1 Aspergillus ruber strain MBRU_F2040 beta-tubulin (BenA) gene, partial cds  GGTGCTGCTTTCTGGTATGTCCATAATATTGGGGTTCGGATTGAGTGATGTACTAACAGTATCATAGGCAGACTATCTCCGGCGAGCACGGTCTCGACGGCTCTGGTGTGTAAGTATCATCGGGTCTCCGGGATGGACGCGTATCGGATATGGATATCTAATGGACTACAGCTACAATGGCTCCTCTGACCTCCAGTTGGAGCGTATGAACGTCTACTTCAACGAGGTTTGTGCATTTATCTCTCATTGACGGACAGTTCTGACTTGCCAGGCCTCCAACAACAAATATGTCCCTCGTGCCGTCCTTGTCGACCTTGAGCCCGGTACCATGGACGCCGTCCGTGCCGGTCCCTTCGGTCAGCTCTTCCGTCCCGATAACTTCGTCTTCGGTCAGTCCGGTGCTGGTAACAACTGGGCCAAGGG | >MT920939.1 Aspergillus ruber isolate EFA 344.155 calmodulin (caM) gene, partial cds  TTCTCCCTCTTCGTAAGTGCGCTGGCCATCTCCATGTGGCATTTTCCCCGTCGGGCGTCGAGGACTGTAAACTGACCACGATTTTTGCATTTTAACAGGACAAGGATGGCGATGGTTAGTTCTCGTGATTTACTCTAGATTCAATCCCTCTTTTACCCCACCTCTCGATTGGTTCCGACCGTGATATGATATTCGCTGTTGAGATAATGCCTTTCTGGTTAGTGACACAATACTGATGGGTTCCTGCGATTACAGGCCAGATCACCACCAAGGAGCTGGGCACCGTTATGCGCTCGCTGGGCCAGAACCCCTCCGAGTCGGAGTTGCAAGATATGATCAACGAGGTCGACGCTGACAACAACGGCACCATCGATTTCCCTGGTATGCGATCGCCCTGATATAAAACTTCCGTAACAAGATTGACGATTCTGACCATAACAGAATTCCTTACCATGATGGCACGGAAGATGAAGGACACCGACTCCGAGGAGGAGATCCGGGAAGCTTTCAAGGTCTTCGATCGCGACAACAACGGTTTTATTTCCGCCGCGGAGTTGCGCCACGT |
| Aspergillus uvarum | >NR_135330.1 Aspergillus uvarum ITS region; from TYPE material  CCGAGTGCTGGGTCCTTCGGGGCCCAACCTCCCACCCGTGCTTACCGTACCCTGTTGCTTCGGCGGGCCCGCCTTCGGGCGGCCCGGGGCCTGCCCCCGGGACCGCGCCCGCCGGAGACCCCAATGGAACACTGTCTGAAAGCGTGCAGTCTGAGTTGATTGATACCAATCAGTTAAAACTTTCAACAATGGATCTCTTGGTTCCGGCATCGATGAAGAACGCAGCGAAATGCGATAACTAATGTGAATTGCAGAATTCAGTGAATCATCGAGTCTTTGAACGCACATTGCGCCCCCTGGTATTCCGGGGGGCATGCCTGTCCGAGCGTCATTTCTCCCCTCCAGCCCCGCTGGTTGTTGGGCCGCGCCCCCCCGGGGGCGGGCCTCGAGAGAAACGGCGGCACCGTCCGGTCCTCGAGCGTATGGGGCTCTGTCACCCGCTCTATGGGCCCGGCCGGGGCTTGCCTCGACCCCCAATCTTCTCA | >HE984421.1 Aspergillus uvarum partial tubb gene for beta tubulin, strain ITEM 14819  AACCAAATCGGTGCTGCTTTCTGGTATGTCTCTACTTGAGTGAACGGAGACTTGACATGCTCTGAGGACTCTCACTCAGAGTCAAGAACATTGCTGTCAAAGCTTCTCTGTGTCCAATTGAGCTAACAGTCTTCCAGGCAGACCATCTCTGGTGAGCACGGCCTCGACGGCGCCGGTGTGTAAGTACAGACTTCCCAGGCGGCAGGAACAGCGGGTCGCGGAAGATGGTGAAGGATATTAATGGAAAGGACAGTTACAATGGCTCCTCCGACCTCCAGCTGGAGCGCATGAACGTCTACTTCAACGAGGTTCGTCCTCGACTCCTTTCCTCCGGCTTTCCCGCATCGGCTTCCTGACTCCTTCACCAGGCTAGCGGCAACAAGTATGTCCCCCGTGCCGTCCTCGTCGACCTCGAGCCCGGTACCATGGACGCCGTCCGTGCCGGTCCTTTCGGCCAACTGTTCCGCCCCGACAATTTCGTCTTTGGTCAATCTGGCGCTGGTAACAACTGGGCCAAGGGTCACTACACTGA | >MK919493.1 Aspergillus uvarum strain AS13 calmodulin (caM) gene, partial cds  GACCCCGATGCTTTCTCGTCTGCTATGAAACAATGTTTACTGATTCATTTCTCGTGATCCCTGATAGGACAAGGATGGCGATGGTTAGTGTAGTTCCTTACCCCTCAACGAAACCCGCCTGGCACCACTCTAGCCACCACACTGCAGACGCCACCGAAACCACTGAGAATCGATCGCATTTCTGACAGAATCTATGTTACAGGACAAATTACCACCAAGGAGTTGGGTACCGTTATGCGTTCCCTCGGCCAGAACCCCTCCGAGTCTGAGCTCCAGGATATGATCAACGAGGTCGACGCTGACAACAACGGCACTATCGACTTCCCCGGTATGTGATAGCCATGCATCGGGCTCCAGACATAGTATTGACAGCTGCCCAGAATTCCTTACCATGATGGCCCGTAAGATGAAGGATACCGATTCCGAGGAGGAGATCCGTGAGGCTTTCAAGGTCTTCGATCGCGATAACAACGGCTTCATC |
| Aspergillus arachidicola | >Aspergillus arachidicola CBS 117610 ITS region;  GGTTCCTAGCGAGCCCAACCTCCCACCCGTGTTTACTGTACCTTAGTTGCTTCGGCGGGCCCGCCGTCATGGCCGCCGGGGGCGTCAGCCCCGGGCCCGCGCCCGCCGGAGACACCACGAACTCTGTCTGATCTAGTGAAGTCTGAGTTGATTGTATCGCAATCAGTTAAAACTTTCAACAATGGATCTCTTGGTTCCGGCATCGATGAAGAACGCAGCGAAATGCGATAACTAGTGTGAATTGCAGAATTCCGTGAATCATCGAGTCTTTGAACGCACATTGCGCCCCCTGGTATTCCGGGGGGCATGCCTGTCCGAGCGTCATTGCTGCCCATCAAGCACGGCTTGTGTGTTGGGTCGTCGTCCCCTCTCCGGGGGGGACGGGCCCCAAAGGCAGCGGCGGCACCGCGTCCGATCCTCGAGCGTATGGGGCTTTGTCACCCGCTCTGTAGGCCCGGCCGGCGCTTGCCGAACGCAAAACAACCATTCTTTCCA | >MN993909.1 Aspergillus arachidicola voucher KGUT_MD12 beta-tubulin (benA) gene, partial cds  AACTTCCAGTTAATATGCTCTGGTCCAAGGAACTCCTCAAAAGCATGATCTCGGATGTGTCCTATTATATCTGCCACGTGTTTGCTAACAACTTTGCAGGCAAACCATCTCTGGCGAGCACGGTCTTGACGGCTCCGGTGTGTAAGTACAGCCTGTATACACCTCGAACGAACGACGACCATATGGCATTAGAAGTTGGAATGGATCTGACGGCAGGGATAGTTACAATGGCTCCTCTGATCTCCAGCTGGAGCGTATGAACGTCTACTTCAACGAGGTGCGTACCTCAAAATTTTCAGCATCTATGAAAACGCTTTGCAACTCCTGACCGCTTCTCCAGGCCAGCGGAAACAAGTATGTCCCTCGTGCCGTCCTCGTCGATCTTGAGCCTGGTACCATGGACGCCGTCCGTGCCGGTCCCTTCGGTCAGCTCTTCCGTCCCGACAACTTCGTTTTCGGCCAGTCCGGTGAG | >MW525378.1 Aspergillus arachidicola strain 149 calmodulin (CaM) gene, partial cds  AACACAAGCTGACTTGGCTTTTCTTGGGTTTCCTATAGGACAAGGACGGTGATGGTTAGTACAGCTCATTTTATTCATTCTCCTTTCAAATGCGACCAATATATTTTAGCCGCCATAAGTTTATCAAGTTTCTGTTCGATCGGCTGAAGTCTTGGCATTGATGAATTGACTTGATATGCAGGCCAGATCACCACCAAGGAGTTGGGCACTGTCATGCGCTCTCTGGGTCAAAACCCCTCTGAGTCGGAACTCCAGGACATGATTAACGAGGTTGACGCCGACAACAATGGCACCATTGACTTCCCTGGTACGAGACGGCTTCCGTACGATTCATAAATGAAATAATTGTTAATCGTCCAAATAGAATTCCTTACGATGATGGCGAGAAAGATGAAGGATACCGACTCTGAGGAGGAGATCCGGGAGGCTTTCAAGGTTTTCGACCGCGATAACAACGGCTTCATCTCCGCTG |
| Aspergillus clavatus | >NR_121482.1 Aspergillus clavatus ATCC 1007 ITS region; from TYPE material  AACGACCCCCCAGAGCCGGAAAGTTGGTCAAACCCGGTCATTTAGAGGAAGTAAAAGTCGTAACAAGGTTTCCGTAGGTGAACCTGCGGAAGGATCATTACCGAGTGCGGGCCCTCTGGGTCCAACCTCCCACCCGTGTTTATCGTACCTTGTTGCTTCGGCGGGCCCGCCGTCTTCGGACGGCCGCCGGGGAGGCCTCCGCGCCCCCGGGCCCGCGCCCGCCGAAGACCACAACATGAACTCTGTTCTGAAGTTTTGCAGTCTGAGTTGATTATCATAATCAGTTAAAACTTTCAACAACGGATCTCTTGGTTCCGGCATCGATGAAGAACGCAGCGAAATGCGATAACTAATGTGAATTGCAGAATTCAGTGAATCATCGAGTCTTTGAACGCACATTGCGCCCCCTGGTATTCCGGGGGGCATGCCTGTCCGAGCGTCATTGCTGCCCTCAAGCACGGCTTGTGTGTTGGGCCCCCGTCCCCGGTTTCCCGGGGACGGGCCCGAAAGGCAGCGGCGGCACCGCGTCCGGTCCTCGAGCGTATGGGGCTTTGTCACCCGCTCTTGTAGGGCCGGCCGGCGCCTGTCGACACCAACCCAATTTTTCTAAGGTTGACCTCGGATCAGGTAGGGATACCCGCTGAACTTAAGCATATCAATAAGCGGAGGAAAAGAAACCAACAGGGATTGCCTCAGTAACGGCGAGTGAA | >KJ767723.1 Aspergillus clavatus strain LEMI40 beta-tubulin (BenA) gene, partial cds  TTGGTAACCAGATTGGTGCTGCTTTCTGGTATGTATTAATGCATTGATGTTGATGTTGATGATGGAAGATTCAGAGCCATACAATCTCAATTGGCAATTCGCCTGGGGGTTGTGGGTTCCAGTTCGTGGATATGGGCTGACAGATTTATAGGCAGACCATCTCTGGTGAGCACGGCCTTGACGGCTCTGGCCAGTAAGTGCAACCTTTTATTCCGATCGATATGGCGGAAGGAAATATGAGGAGGATTACAACACTGAGCATGGATCTGATGGAAATGATAGCTACAATGGCACCTCCGATCTCCAGCTCGAGCGTATGAACGTCTACTTCAACGAGGTGCGTGGTGGGATTCCGTATGCTTCGACAAAACCTTCACGATCCTAACTCCCTGCCAGGCCAACGGTGACAAGTATGTTCCTCGTGCCGTCCTCGTCGATCTCGAGCCCGGTACCATGGACGCCGTCCGTGCTGGTCCATTCGGCGAGCTTTTCCGTCCCGACAACTTCGTTTTCGGCCAGTCTGGTGCTGGTAACAACTGG | >MK451350.1 Aspergillus clavatus strain CMV013B2 calmodulin gene, partial cds  GCCTTCTCTCTTTTCGTAAGTGATCCTGCCTAATTCTCGAATGGTCTATGAGTGAAATTCGGGCCATGTTATGTTCTTGAAGGCATTCACTTACATGGGATTCTCCGATCTGTACAGGACAAGGATGGTGATGGTTAGTTCGGCCTTCTCTTCCAAGAACCACCTTCTATGCGACTACTTCCGGCCACCGATATTTCTATTCTCCGTAAAGGAAGCGATCCATGTTTGCTGATGTATTTCTCGTCGACCTGCAGGCCAGATCACCACCAAGGAGTTGGGTACCGTTATGCGCTCTCTGGGCCAGAACCCTTCCGAGTCGGAGCTCCAGGACATGATCAATGAGGTTGATGCCGACAACAACGGCACCATTGATTTTCCCGGTATGCGATACTTCCCACCCGAATCACTCAGAGAGATGAATATATATTGACCCGCGTTCAGAGTTCCTCACCATGATGGCTCGGAAGATGAAGGATACCGACTCTGAGGAGGAAATCCGAGAGGCTTTCAAGGTTTTCGATCGTGACAACAACGGTTTCATTTCCGCCGCGGAGCTGCGCCACGT |
| Aspergillus novofumigatus | >NR_171617.1 Aspergillus novofumigatus CBS 117520 ITS region; from TYPE material  CCGAGTGAGGGCCCTCTGGGTCCAACCTCCCACCCGTGTCTATCGTACCTTGTTGCTTCGGCGGGCCCGCCGTTTCGACGGCCGCCGGGGAGGCCTCGCGCCCCCGGGCCCGCGCCCGCCGAAGACCCCAACATGAACGCTGTTCTGAAAGTATGCAGTCTGAGTTGATTATCATAATCAGTTAAAACTTTCAACAACGGATCTCTTGGTTCCGGCATCGATGAAGAACGCAGCGAAATGCGATAAGTAATGTGAATTGCAGAATTCAGTGAATCATCGAGTCTTTGAACGCACATTGCGCCCCCTGGTATTCCGGGGGGCATGCCTGTCCGAGCGTCATTGCTGCCCTCAAGCACGGCTTGTGTGTTGGGCCCCGTCCCCTCCCCCGGGGACGGGCCCGAAAGGCAGCGGCGGCACCGCGTCCGGTCCTCGAGCGTATGGGGCTTTGTCACCCGCTCTGTAGGCCCGGCCGGCGCCAGCCGACACCCAACTTTATTTCTAA | >DQ094886.1 Aspergillus novofumigatus strain IBT 16806 beta-tubulin gene, partial sequence  TGGTAAGTCTCGACCTCAATGCTTGGATGATGGGAGACTGGGACCTGTCATCTTAGCAGGCTGTCCTCCATTGGGATCAGCTTCGCTGTCATAGGTATCAGCTAACAAATTTACAGGCAGACCATCTCTGGTGAGCACGGCCTTGACGGCTCTGGCCAGTAAGTTCGACCTTTATCCTCCCAATTGAGAAAGCGGGGGAAACACGAAAGG  CAAGGAAGAAGAGGACGCGTGTCTGATTGGGATAATAGCTACAATGGCTCCTCCGACCTCCAGCTGGAGCGTATGAACGTCTATTTCAACGAGGTGCGTTGATGAAACTCTCGACTCTGCACTACTTCGGCAATTTCTCACGATCTAACTTGCTACTAGGCCAACGGTGACAAGTATGTTCCTCGTGCTGTTCTGGTCGATCTCGAGCCC  GGTACCATGGACGCTGTCCGTGCCGGTCCCTTCGGCGAGCTCTTCCGTCCCGACAACTTCGTCTTCGGCCAGTCTGGTGCTGGTAACAACTGG | >FR839688.1 Aspergillus novofumigatus partial caM gene for calmodulin, isolate CCF 4078, exons 1-4  TGAGGAACAGGTTTCCGAGTACAAGGAGGCTTTCTCTCTCTTCGTAAGTGAACTGTCCAAGTCCCTAGTCGTTGCATAAGAGGAATTTCCTCAATATTGAGGGTGTGCGCTCACACGAGATTCGACCTATAGGACAAGGATGGTGATGGTTAGTGACCTTTTTTTCGCTCCCCGAACGTCGGCTTCCGTGCGATCATGTTCAAATGCCGACTTGCAATGTCCGGAAATGATCCATCAATACTGATAATATCTATGTTTGACTATTTAGGCCAGATCACCACCAAGGAATTGGGCACTGTGATGCGCTCCCTGGGCCAGAACCCTTCCGAGTCAGAGCTGCAAGATATGATCAACGAGGTGGATGCTGACAACAACGGCACCATCGATTTCCCTGGTATGCGATGCATTTGGTGTGACCTTGGGAGGGAGGAGAACAATCATTAACTCGTATTCAGAATTCCTTACTATGATGGCTCGGAAGATGAAGGACACCGACTCCGAAGAGGAAATCCGGGAAGCTTTCAAGGTCTTCGACCGCGACAACAACGGTTTCATCTCCGCTGCGGAGCTGCGCCACGTTATGACCTCTATCGGGGAGAAGCTCACTGATGACGAAGTTGACGAGATGAT  CCGCGAGGCGGATCAGGACGGTGACGGCCGGATTGATTGTATGTTGATCAAACAGCTT |
| Aspergillus spinulosporus | >NR_137459.1 Aspergillus spinulosporus NRRL 2395 ITS region; from TYPE material  AAGGATCATTACCGAGTGCGGGCTGCCTCCGGGCGCCCAACCTCCCACCCGTGACTACCTAACACTGTTGCTTCGGCGGGGAGCCCCCCAGGGGGCGAGCCGCCGGGGACCACTGAACTTCATGCCTGAGAGTGATGCAGTCTGAGCCTGAATACAAATCAGTCAAAACTTTCAACAATGGATCTCTTGGTTCCGGCATCGATGAAGAACGCAGCGAACTGCGATAAGTAATGTGAATTGCAGAATTCAGTGAATCATCGAGTCTTTGAACGCACATTGCGCCCCCTGGCATTCCGGGGGGCATGCCTGTCCGAGCGTCATTGCTGCCCTCAAGCCCGGCTTGTGTGTTGGGTCGTCGTCCCCCCCCCGGGGGACGGGCCCGAAAGGCAGCGGCGGCACCGTGTCCGGTCCTCGAGCGTATGGGGCTTTGTCACCCGCTCGATTAGGGCCGGCCGGGCGCCAGCCGGCGTCTCCAACCTTATTTTTCTCAGGTTGACCTCGGATCAGGTAGGGATACCCGCTGAACTTAAGCATATCAATAAGCGGAGGAAAAGAAACCAACCGGGATTGCCTCAGTAACGGCGAGTGAA | >EF652269.1 Aspergillus spinulosporus isolate NRRL 2395 beta-tubulin gene, partial cds  TGGTGAGTCGAAAATTTCACTTTTTTTTGGAATGACTTCTTCATACACTTGCTAACAAGCCTACAGGCAGACCATCTCCGGTGAGCACGGCCTCGATGGCTCCGGTGTGTGAGTACAATCCGTCTAGCATTCGATGAAAACACGACACCACAACAGAACCTGACACAACCCAGTTACAATGGTACCTCCGACCTTCAGCTCGAGCGTATGAACGTCTACTTCAATGAGGTCAGTGGATGAGATATCAGGTAGTGGTTGGGGTGTGTCTGACTTCGTCCAGGCCAGCGGTAACAAGTACGTTCCCCGTGCCGTTCTCGTCGATCTCGAGCCCGGTACTATGGACGCTGTCCGCGCCGGTCCTTTCGGCGAGCTCTTCCGTCCCGACAACTTCGTTTTCGGCCAGTCCGGTGCTGGTAACAACTGG | >EF652357.1 Aspergillus spinulosporus isolate NRRL 2395 calmodulin gene, partial cds  GTCTCCGAGTACAAGGAGGCCTTTTCCCTGTTTGTAAGTGCCATTGGTTGATATTATGTCAGTTTCGAGTTATATTGAAAGTATACTAATATATTCCGCGCTTAACAGGACAAGGATGGCGATGGTTAGTGCATTTGTCCCCCCAGAACTGATCGCATTCGCCCAGCGTCGGTGTTGTAGTTCTATATAAACCGATTCTGATAAACGGCGATAGGCCAGATTACCACTAAGGAGCTTGGCACTGTCATGCGCTCGCTCGGTCAGAACCCTTCAGAGTCTGAACTTCAGGACATGATCAACGAAGTTGATGCCGACAACAACGGCACCATTGACTTCCCAGGTCCGTGATCTCCCAACCTACTTCGCACAGCCAGTTAGAAACTGTACTAATTCCTAAACAGAGTTCCTTACCATGATGGCCAGAAAAATGAAGGACACCGATTCCGAGGAGGAAATTCGGGAGGCGTTCAAGGTGTTCGACCGTGACAACAATGGTTTCATCTCCGCTGCTGAGCTGCGTCACGTCATGACCTCTATCGGCGAGAAGCTCACCGATGACGAAGTTGACGAGATGATCCGCGAGGCGGATCAGGATGGTGACGGCCGAATTGACTGTGAGTTGGCTTCCCGCTTATCCTTGACCGTAGAAGAAATATGATGCTGATCAGCGGCAGACAACGAATTC |
| Aspergillus niger | >NR_111348.1 Aspergillus niger ATCC 16888 ITS region; from TYPE material  GTAGGTGAACCTGCGGAAGGATCATTACCGAGTGCGGGTCCTTTGGGCCCAACCTCCCATCCGTGTCTATTGTACCCTGTTGCTTCGGCGGGCCCGCCGCTTGTCGGCCGCCGGGGGGGCGCCTCTGCCCCCCGGGCCCGTGCCCGCCGGAGACCCCAACACGAACACTGTCTGAAAGCGTGCAGTCTGAGTTGATTGAATGCAATCAGTTAAAACTTTCAACAATGGATCTCTTGGTTCCGGCATCGATGAAGAACGCAGCGAAATGCGATAACTAATGTGAATTGCAGAATTCAGTGAATCATCGAGTCTTTGAACGCACATTGCGCCCCCTGGTATTCCGGGGGGCATGCCTGTCCGAGCGTCATTGCTGCCCTCAAGCCCGGCTTGTGTGTTGGGTCGCCGTCCCCCTCTCCGGGGGGACGGGCCCGAAAGGCAGCGGCGGCACCGCGTCCGATCCTCGAGCGTATGGGGCTTTGTCACATGCTCT  GTAGGATTGGCCGGCGCCTGCCGACGTTTTCCAACCATTCTTTCCAGGTTGACCTCGGATCAGGTAGGGATACCCGCTGAACTTAA | >EU982068.1 Aspergillus niger strain ATHUM 2539 BenA (benA) gene, partial cds  TGGTACGTATACAACTGCCATTGGACTTGGGGCTGGCACATCGTCTCTTAGGCTATCTCAGCTTGAGTTCAGACTGTTGTCCATTAGGTCCATGCTATCGGTCTAAGCACACGTCTAACAATTCAACAGGCAGACCATCTCTGGCGAGCACGGCCTTGACGGCTCCGGTGTGTAAGTGCAACTTTTTCACACCTCTCAATTGGTCAACAATGGGCAAAGGGTTGGGTCTTCTGACACGCAGGATAGTTACAATGGCACCTCCGACCTCCAGCTGGAGCGCATGAACGTCTACTTCAACGAGGTGAGATCCATCGGACCTTGGCTTTTTCACGACAATATCATCAATGTCCTAATCACTTCAGCAGGCTAGCGGTAACAAGTATGTTCCTCGTGCCGTCCTCGTCGACCTCGAGCCCGGTACCATGGACGCCGTCCGTGCCGGTCCTTTCGGCCAGCTCTTCCGCCCCGACAACTTCGTCTTCGGCCAGTCCGGTGCTGGTAACAACTGG | >FN594567.1 Aspergillus niger partial cam gene for calmodulin, strain MUCL 30480  TGATCGATGAGCTATCTTTACCGGAGCATAATGCTAATGTGTTTTCGGACTTAATAGGACAAGGATGGCGATGGTGGGTGGAATTCTATCCCCTTCACATTATACCTGTAGCGCTCGATCCGACTGCGGGATTTCGACAGCATTTTCCAGAACGATTTGGATCATAATACTAATTTAAATCGGTGAATCAGGCCAGATCACCACCAAGGAGCTCGGCACTGTGATGCGCTCCCTTGGCCAGAACCCCTCCGAGTCTGAGCTTCAGGACATGATCAACGAGGTTGACGCTGACAACAACGGAACGATCGACTTCCCCGGTATGTGTTAGATTTACGCCTGTAAGGCGGAAATGCGGGCTGGATTGTGATTGACTTTTGCCGCCAGAATTCCTTACCATGATGGCTCGTAAGATGAAGGACACCGACTCCGAGGAGGAAATCCGCGAGGCTTTCAAGGTCTTCGACCGCGACAACAATGGTTTTATCTCCGCCGCGGAGCTGCGCCACGTCATGACCTCCAT |
| Aspergillus melleus | >NR_103610.1 Aspergillus melleus CBS 546.65 ITS region; from TYPE material  CTGAGTGAGGGTCCCTCGGGGCCCAACCTCCCACCCGTGTATACCGTACCTTGTTGCTTCGGCGAGCCCGCCCCCTTCTCGTTAGGGGGGCACAGCGCTCGCCGGAGACACCAACGTGAACACTGTCTGAAGTTTTGTCGTCTGAGTCGATTGTATCGCAATCAGTTAAAACTTTCAACAATGGATCTCTTGGTTCCGGCATCGATGAAGAACGCAGCGAAATGCGATAATTAATGTGAATTGCAGAATTCAGTGAATCATCGAGTCTTTGAACGCACATTGCACCCCCTGGTATTCCGGGGGGTATGCCTGTCCGAGCGTCATTGCTGCCCTCAAGCACGGCTTGTGTGTTGGGTCGTCGTCCCCCCGGGGACGGGCCCGAAAGGCAGCGGCGGCACCGCGTCCGGTCCTCGAGCGTATGGGGCTTTGTCACACCGCTCTTGTAGGCCCGGCCGGCTGCTGGCCGACGCTGAAAAGCAACCAACTATTTCTCCA | >LN851557.1 Aspergillus melleus tub2 gene for beta-tubulin, isolate MUT4199  TCCAGGATCATCTTCGATACCTTGATGACCTATGACTCTCAATCCTTGATACTTGATACTTGTTTACTGATAGGTGAATAGGCAAAACATCTCTGGCGAGCACGGCCTTGATGGCGCCGGTGTGTAAGTACATCCCGCGTTTACACCTATCAAAATCAGAATCGACGAGAGAAGAAAAGAAAGAAATGATCATGGTGGGATTGGTTGTCT  GATGGGGTGAACAGTTACAATGGCTCCTCCGACCTTCAGCTGGAGCGCATGAACGTCTACTTCAACGAGGTTCGTTGCCCGAAAATTTTCTATCTCCTTTCGCCGATCCGAAACGCCCCGTACAAAGCTCTAACCCACGCCTTCTTCATCTTCTAGGCTTCCGGTGGCAAGTATGTTCCCCGTGCCGTTCTGGTCGATCTTGAGCCCGGTACCATGGACGCTGTCCGTGCCGGTCCCTTCGGTCAGCTTTTCCGCCCCGACAACTTCGTCTTCGGCCAGTCTGGTGCCGGTAACAACT | >MZ028008.1 Aspergillus melleus strain DTO 438-H7 calmodulin (cmdA) gene, partial cds  CCGAGTACAAGGAGGCCTTCTCCCTGTTCGTAAGTGTTCCATTCTTCCTGATGTAATGATGGGGAAACGAAGCGATCTTGCTTTGTTTTTTACGCCTTCAGCCTTATGGGAATATTCCAAGCTCACTTGAGATTTTGCCTCTTCACAGGACAAGGATGGCGATGGTTAGTGCTATTCCGGTTTCCCTTCCAGTTCATCGACTCACGCGACCGGCTGTTTTCCGTCGATATGGAATTTTCTTTCACCCTGTTGCTTCCGCCGACCGATTTGCGACCAGGACGCTAATTTGTGAATCCACGCTGCAGGCCAGATCACCACCAAGGAGTTGGGCACTGTTATGCGCTCGCTGGGCCAGAACCCCTCCGAGTCTGAGTTGCAGGACATGATCAACGAAGTTGACGCCGACAACAATGGCACCATCGATTTCCCCGGTATGCGATGATGGACACACCGATATCCGGAAAGGGAGACAATCATGACACTCAGCTGCTAACCTCACGCAGAGTTCCTGACGATGATGGCCCGAAAGATGAAGGATACCGATTCTGAGGAGGAAATCCGGGAAGCTTTCAAGGTCTTCGATCGCGATAACAACGGTTTCATCTCGGCCGCGGAGCTGCGCCACGTCATGA |
| Aspergillus neoniger | >NR_137505.1 Aspergillus neoniger CBS 115656 ITS region; from TYPE material  CCTCCCATCCGTGTCTATTATACCCTGTTGCTTCGGCGGGCCCGCCGCTTGTCGGCCGCCGGGGGGGCGCCTTTGCCCCCCGGGCCCGTGCCCGCCGGAGACCCCAACACGAACACTGTCTGAAAGCGTGCAGTCTGAGTTGATTGAATGCAATCAGTTAAAACTTTCAACAATGGATCTCTTGGTTCCGGCATCGATGAAGAACGCAGCGAAATGCGATAACTAATGTGAATTGCAGAATTCAGTGAATCATCGAGTCTTTGAACGCACATTGCGCCCCCTGGTATTCCGGGGGGCATGCCTGTCCGAGCGTCATTGCTGCCCTCAAGCCCGGCTTGTGTGTTGGGTCGCCGTCCCCCTCTCCGGGGGGACGGGCCCGAAAGGCAGCGGCGGCACCGCGTCCGATCCTCGAGCGTATGGGGCTTTGTCACATGCTCTGTAGGATTGGCCGGCGCCTGCCGACG | >MT410059.1 Aspergillus neoniger isolate CCMFBH-839 beta-tubulin (benA) gene, partial cds  TAATGGTGCTGCTTTCTGGTACGTATTCACTGCCACTGGATTGGGGATGGAACATCATCTCTCAAGCTATCTTAGCTTGAGTTCAGATGTTATCCATCGGATATATAGCTATCGGTTTAAGAACACGTCTAACAACTCAACAGGCAGACCATCTCTGGCGAGCACGGCCTTGACGGCTCCGGTGTGTAAGTACAACTTTTTCACACCTCTCAATTGGTCAACAATGTGGAAAGGATTGGGTTTCCTGACGCGCAGGATAGTTACAATGGCACCTCCGACCTCCAGCTGGAGCGCATGAACGTCTACTTCAACGAGGTTAGATCACACCGTCCCTGAGTTTTTCACGACAATATCATCAATGTCCTGACCACTTCAGCAGGCTAGCGGTAACAAGTATGTCCCCCGTGCCGTCCTCGTCGATCTCGAGCCCGGTACCATGGACGCCGTCCGTGCCGGTCCCTTCGGCCAGCTCTTCCGCCCCGACAACTTCGTCTTCGGCCAGTCCGGTGCTGGTAACAACTGGGCCAAGGGT | >MH644982.1 Aspergillus neoniger voucher IHEM 18106 calmodulin (caM) gene, exons 1 through 5 and partial cds  CAAGTTTCTGAGTACAAGGAGGCCTTCTCCCTCTTTGTGAGTACTCCCTGAATGAACCCCCGATCACTCAAATTGATGACCTATCTTTCCCGGCTCATAACGCTAATGTATTTTCGAACTCAATAGGACAAGGATGGCGATGGTGGGTGGAATCCTGTCCCCTTCACGTTTTACCCGTAGCGCTCGATCCGACCGCGGGATTTCGACTGCAATTCCCCCATCGATCTGAATCATTATACTGATGTAATCTGGAAATAGGCCAGATCACCACCAAGGAGCTCGGCACTGTGATGCGCTCCCTCGGCCAGAACCCCTCCGAGTCTGAGCTTCAGGACATGATCAACGAGGTTGACGCTGACAACAACGGAACGATCGACTTCCCCGGTATGTGATAGATCTACGCCTGTAAGGCGGGAATGCCGTATGGGTTGTGATTGACTTTTGCCGCCAGAATTCCTCACCATGATGGCTCGTAAGATGAAGGACACCGACTCCGAGGAGGAAATCCGCGAGGCTTTCAAGGTCTTCGACCGCGACAACAATGGTTTCATCTCCGCCGCGGAGTTGCGCCACGTCATGACCTCCATTGGTGAGAAGCTCACTGACGACGAAGTCGATGAGATGATCCGTGAGGCTGACCAGGACGGTGATGGCCGCATCGACTGTATGTTTCCCATTCTTGATATGCCCATGATATGACATGCTAACTCTGCTACCAGACAACGAG |
| Aspergillus brasiliensis | >NR_111414.1 Aspergillus brasiliensis ATCC MYA-4553 ITS region; from TYPE material  ATCATTACCGAGTGCGGGTCCTTTGGGCCCAACCTCCCATCCGTGTCTATTGTACCCTGTTGCTTCGGCGGGCCCGCCGCTTGTCGGCCGCCGGGGGGGCGCCTCTGCCCCCCGGGCCCGTGCCCGCCGGAGACCCCAACACGAACCCTGTCTGAAAGCGTGCAGTCTGAGTCGATTGTTTGCAATCAGTTAAAACTTTCAACAATGGATCTCTTGGTTCCGGCATCGATGAAGAACGCAGCGAAATGCGATAACTAATGTGAATTGCAGAATTCAGTGAATCATCGAGTCTTTGAACGCACATTGCGCCCCCTGGTATTCCGGGGGGCATGCCTGTCCGAGCGTCATTGCTGCCCTCAAGCCCGGCTTGTGTGTTGGGTCGCCGTCCCCTCTCTCCGGGGGGACGGGCCCGAAAGGCAGCGGCGGCACCGCGTCCGATCCTCGAGCGTATGGGGCTTTGTCACATGCTCTGTAGGATTGGCCGGCGCCTGCCGACGTTTTCCAACCATTCTTTCCAGGTTGACCTCGGATCAGGTAGGGATACCCGCTGAACTTAAGCATATC | >EU600387.1 Aspergillus brasiliensis strain CBS 122724 beta-tubulin (benA) gene, partial cds  CATCTCTGGCGAGCACGGCCTTGACGGCTCCGGTGTGTAAGTGCAACTTTTTTCACACCTCTCAATTGGT  CAACAATGGGGAAAGGATTGGGTTTCCTGACGCGCAGGATAGTTACAATGGCACCTCCGACCTCCAGCTG  GAGCGCATGAACGTTTACTTCAACGAGGTGAGATCCATCGGTCCTTGGTTTTGGTACGACAATATCATCA  ATGTCCTGACCACTTCAGCAGGCTAGCGGTAACAAGTATGTTCCTCGTGCCGTCCTCGTTGATCTCGAGC  CCGGTACCATGGACGCCGTCCGTGCCGGTCCTTTCGGCCAGCTCTTCCGCCCCGACAACTTCGTCTTCGG  CCAGTCCGGTGCTGGTAACAACTGGG | >AM295179.1 Aspergillus brasiliensis partial cam gene, strain ITEM 4539  TTGTGAGTGCCCCCTGAATGATCCCGATCGTCCAGAACGATAGCCTATCATCACTGGATCATAATACTGATGTACCTTGGGACTCAATAGGACAAGGATGGCGATGGTGGGTGGAATTCTGTCCCCTTCACGTTTTACCTGTAGCGCCCGGACCGACCGCAGGATCTCGACAGCCATTCTCCAAGCGATCTGAATCATGATACTGATATAATGCGGGAAATAGGCCAGATCACCACCAAGGAGCTCGGCACCGTCATGCGCTCCCTCGGCCAGAACCCCTCCGAGTCTGAGCTTCAGGACATGATCAACGAGGTTGACGCTGACAACAACGGAACGATCGACTTCCCCGGTATGTGATAGATCTACACCTTGAAGGCGGAACACGGGCTGGGTTGTGATTGACTTTTGCCGCCAGAATTCCTTACCATGATGGCTCGTAAGATGAAGGACACCGACTCCGAGGAGGAAATCCGTGAGGCTTTCAAGGTCTTCGACCGCGATAACAATGGTTTCATCTCCGCCGCGGAGCTGCGCCACGTCATGACCTCCATTGGTGAGAAGCTCACCGACGACGAAGTCGACGAGATGATCCGTGAGGCGGATCAGGACGGTGACGGCCGCATTGACTGTATGTTTCTTACCCCCGATGATACCCATACCCTGGTATACTAACTTTGCTACCAGACAACGAGT |
| Aspergillus fumigatiaffinis | >Aspergillus fumigatiaffinis strain CMV001G1 ITS CCGAGTGAGGGCCCTCTGGGTCCAACCTCCCACCCGTGTCTATCGTACCTTGTTGCTTCGGCGGGCCCGCCGTTTCGACGGCCGCCGGGGAGGCCTCGCGCCCCCGGGCCCGCGCCCGCCGAAGACCCCAACATGAACGCTGTTCTGAAAGTATGCAGTCTGAGTTGATTATCATAATCAGTTAAAACTTTCAACAACGGATCTCTTGGTTCCGGCATCGATGAAGAACGCAGCGAAATGCGATAAGTAATGTGAATTGCAGAATTCAGTGAATCATCGAGTCTTTGAACGCACATTGCGCCCCCTGGTATTCCGGGGGGCATGCCTGTCCGAGCGTCATTGCTGCCCTCAAGCACGGCTTGTGTGTTGGGCCCCGTCCCCTCCCCCGGGGACGGGCCCGAAAGGCAGCGGCGGCACCGCGTCCGATCCTCGAGCGTATGGGGCTTTGTCACCCGCTCTGTAGGCCCGGCCGGCGCCAGCCGACACCCAACTTTTATTTCTAA | >MK450913.1 Aspergillus fumigatiaffinis strain CMV001G1 beta-tubulin gene, partial cds  CGGTGCTGCTTTCTGGTATGTCTCGACCTCAATGCTTGGATGATGGGAGATAGGAACCTGTCATCTTAGCAGGCTGTCCTCCATGGGTTCAGCTTCGCTGTCGTGGGTATCAGCTAACAAATCTACAGGCAGACCATCTCTGGTGAGCACGGCCTTGACGGCTCTGGCCAGTAAGTTCGACCTTTATCCTCCCAATTGAGAAAGCGGGGG  AAACACAAAGGCAAGGAAGAAGAGGACGCGTGTCTGATTGGGATAATAGCTACAATGGCTCCTCCGACCTTCAGCTGGAGCGTATGAACGTCTATTTCAACGAGGTGCGTTGATGAAACTTTCGACTCTACACTACTTCGGCAATTTCTCACGATCTAACTCGCTCTAGGCCAACGGTGACAAGTATGTTCCTCGTGCCGTTCTGGTCGATCTCGAGCCCGGTACCATGGACGCTGTCCGTGCCGGTCCCTTCGGCGAGCTCTTCCGTCCCGACAACTTCGTCTTCGGCCAGTCTGGTGCTGGTAACAACTGGGCCAAGGGTC | >DQ094890.1 Aspergillus fumigatiaffinis strain IBT 13131 calmodulin gene, partial sequence  GTAAGTGAACTGTCCAGTCCCTAGTCGTTGCATAAGAGGAATTTCCTCAATATTGAGGGTGTGCGCTCACACGAGATTCGACCTGTAGGACAAGGATGGTGATGGTTAGTGACCTTTTTTTCGCTCCTCGAACGTCGGCTTCCGTGCGATCATGTTCCAAAGCCGACTTACAATATCCGGAAATGATCCATCAATACTGATAATATCTAT  GTTTGACTATTAGGCCAGATCACCACTAAGGAATTGGGCACTGTGATGCGCTCCCTGGGCCAGAACCCTTCCGAGTCAGAGCTGCAAGATATGATCAACGAGGTGGATGCTGACAACAACGGCACCATCGATTTCCCTGGTATGCGATGCATTTGGTGTGACCTCGGGAGGGAGGAGAATAATCATTAACTCGTATTCAGAATTCCTTACTATGATGGCTCGGAAGATGAAGGACACCGACTCCGAAGAGGAAATCCGGGAAGCTTTCAAGGTCTTCGACCGCGACAATAAC |
| Aspergillus saccharolyticus | >NR_135441.1 Aspergillus saccharolyticus ITS CCGAGTGCTGGGTCCTTCGGGGCCCAACCTCCCACCCGTGTCTACCGTACCTCAGTTGCTTCGGCGGGCCCGCCTTAACGGTGGCCCGGGGGCTTGCCCCCGGGACAGCGCCCGCCGGAGACCCTTTAAACAAGAACCCTTGCCATGAATGCCTTGTAGTCTGAGTTGATGATTAAATTCATTAAAACTTTCAACAATGGATCTCTTGGTTCCGGCATCGATGAAGAACGCAGCGAAATGCGATAACTAATGTGAATTGCAGAATTCAGTGAATCATCGAGTCTTTGAACGCACATTGCGCCCCCTGGCATTCCGGGGGGCATGCCTGTCCGAGCGTCATTTCGCCCCTCCAGCCCGGCTGGTTGTTGGGCCTCGCCCCCCCCGGGGGGCGGGCCCCGAGAGAAATGGCGGCACCGTCCGGTCCCCGAGCGTATGGGTTTACCCGCTCTTAGGGCCCGGTCGGGGCTTTAGCCTCACCCCCAATCTTTCAA | >HM853553.1 Aspergillus saccharolyticus beta-tubulin gene, partial cds  TGTTGTCGAAGCTTCGTGGACAATCAAGCTGACAGTCCTCCAGGCAGACCATCTCTGGTGAGCACGGCCTTGATGGCGCTGGTGTGTAAGTGCAGACAACCCCCAGATTGCGGGCCAAGCGGGTGCGCGGCAAAGCGTGCAAGTGATTAATAAAAAGCCCAGTTACAATGGCTCCTCCGACCTTCAGCTGGAGCGCATGAACGTCTACTTCAACGAGGTTCGTCCTTCCCCGAGTCTTCCCCGCCAGTTTTCCCGCCAGCTCCTCATCCCTTCACCAGGCCAGCGGTAACAAGTATGTTCCTCGTGCCGTCCTCGTCGATCTCGAGCCCGGTACCATGGACGCGGTCCGTGCCGGTCCCTTCGGCCAGCTTTTCCGCCCCGACAACTTCGTTTTCGGTCAGTCCGGTGCTGGTAACAACTGGGCCAAGGGTCAC | >HM853554.1 Aspergillus saccharolyticus calmodulin gene, partial cds  GTGCTTTAGAACAATATTTACTGATGCTCTTGTAATCCCTAACAGGACAAGGATGGCGATGGTTAGTGCAGTCCTTACCCCTCAATCAATCCACCCGGTACCATGATTATCACGACGCTACAACCATGACACCCTCCCGAATAGTGAACAAGATTTGATTGATTTTACATGACAGGACAAATCACCACCAAGGAGTTGGGTACTGTTATG  CGTTCCCTGGGCCAGAACCCTTCCGAGTCTGAGCTCCAGGACATGATTAATGAGGTCGATGCTGACAACAACGGCACTATCGACTTTCCCGGTATGTGATGGTGATGAGTGGAGCTCGAGCATAAGATTGACAGGTACCAGAATTCCTTACAATGATGGCCCGTAAGATGAAAGACACCGATTCTGAGGAGGAAATCCGGGAGGCTTTCAAAGTCTTCGATCGCGACAACAACGGCTTCATTTCCGCCGCGGAGCTGCGCCACGTTATGACCTTCTATCGAAC |
| Aspergillus japonicus | >NR_131268.1 Aspergillus japonicus CBS 114.51 ITS region; from TYPE material  CCGAGTGCTGGGTCCTTCGGGGCCCAACCTCCCACCCGTGCTTACCGTACCCTGTTGCTTCGGCGGGCCCGCCTTCGGGCGGCCCGGGGCCTGCCCCCGGGACCGCGCCCGCCGGAGACCCCAATGGAACACTGTCTGAAAGCGTGCAGTCTGAGTTGATTGATACCAATCAGTTAAAACTTTCAACAATGGATCTCTTGGTTCCGGCATCGATGAAGAACGCAGCGAAATGCGATAACTAATGTGAATTGCAGAATTCAGTGAATCATCGAGTCTTTGAACGCACATTGCGCCCCCTGGTATTCCGGGGGGCATGCCTGTCCGAGCGTCATTTCTCCCCTCCAGCCCCGCTGGTTGTTGGGCCGCGCCCCCCGGGGGCGGGCCTCGAGAGAAACGGCGGCACCGTCCGGTCCTCGAGCGTATGGGGCTCTGTCACCCGCTCTATGGGCCCGGCCGGGGCTTGCCTCGACCCCCAATCTTCTCA | >MH614586.1 Aspergillus japonicus voucher IHEM 5627 beta-tubulin (benA) gene, exons 3 through 6 and partial cds  AGTGTGTAAGTGAACTCGTGCTGGGACACAACACCTTTGTGGTTCGGTATATTCATGCAGTGGACTAAAACATGGGTGCTGGTGACAGGGTAACCAAATTGGTGCCGCTTTTTGGTATGTCTCTACTTGAGTGAACGGAGACTTGACATGCTCTGAGGACCTGTCAAAGCTTCTCTGTGTCCAATTGAGCTAACAGTCTTCCAGGCAGAC  CATCTCTGGTGAGCACGGCCTCGACGGCGCCGGTGTGTAAGTACAGGCTTCCCAGGCGGCAGGAACAGCGGGTCGCGGAAGATGGTGAAGGATATTAATGGAAAGGACAGTTACAATGGCTCCTCCGACCTCCAGCTGGAGCGCATGAACGTCTACTTCAACGAGGTTCGTCTTCGACTCCTTTCCTCCGGCTGTCCCGCATCGGCTTCC  TGACTCCTTCACTAGGCTAGCGGCAACAAGTATGTCCCCCGTGCCGTCCTCGTCGACCTCGAGCCCGGTACCATGGACGCCGTCCGTGCCGGTCCTTTCGGCCAGCTTTTCCGCCCCGACAACTTCGTCTTCGGTCAATCCGGTGCCGGTAACAACTGGGCCAAGGGT | >MW086637.1 Aspergillus japonicus strain 16.548 calmodulin (CaM) gene, partial cds  ACCACTATAGCCACCACACTGCAAATGCCACCGAAACCGCTGAGAATCGATCGCATTTCTGACAGAATCTATGTTACAGGACAAATTACCACCAAGGAGTTGGGTACCGTCATGCGTTCCCTCGGCCAGAACCCCTCCGAGTCTGAGCTTCAGGACATGATCAACGAGGTCGACGCTGACAACAACGGCACTATCGATTTTCCCGGTATG  TGATAGCCATGTATGGGACTCCGGACATAGTATTGACAGCTGCCCAGAATTCCTTACCATGATGGCCCGTAAGATGAAGGATACCGACTCCGAGGAGGAGATCCGTGAGGCCTTCAAGGTTTTCGATCGCGATAATAACGGCTTCATCTCTGCCGCGGAGCTGCGCCACGTCATACCCTCTAT |
| Aspergillus pseudonomiae | >NR_137444.1 Aspergillus pseudonomiae NRRL 3353 ITS region; from TYPE material AAGGATCATTACCGAGTGTAGGGTTCCTAGCGAGCCCAACCTCCCACCCGTGTTTACTGTACCTTAGTTGCTTCGGCGGGCCCGCCGCAAGGCCGCCGGGGGGCATCCGCCCCCGGGCCCGCGCCCGCCGGAGACACCACGAACTCTGAACGATCTAGTGAAGTCTGAGTTGATTGTATCGCAATCAGTTAAAACTTTCAACAATGGATCTCTTGGTTCCGGCATCGATGAAGAACGCAGCGAAATGCGATAACTAGTGTGAATTGCAGAATTCCGTGAATCATCGAGTCTTTGAACGCACATTGCGCCCCCTGGTATTCCGGGGGGCATGCCTGTCCGAGCGTCATTGCTGCCCATCAAGCACGGCTTGTGTGTTGGGTCGTCGTCCCCCTCTCCGGGGGGGGACGGGCCCTAAAGGCAGCGGCGGCACCGCGTCCGATCCTCGAGCGTATGGGGCTTTGTCACCCGCTCTGTAGGCCCGGCCGGCGCTTGCCGAACGCAAAACAACCATTCTTTCCA | >KJ789981.1 Aspergillus pseudonomius strain ITAL 632 beta-tubulin gene, partial cds  GCTTTGGACGTAGGGCCTCAGCAGAAACATGAGCTCGGATATATCATTCTCGTGTCTGACACGTGTTTGCTAATACCTTTGCAGGCAGACCATCTCCGGGGAGCACGGCCTCGACGGCTCCGGTGTGTAAGTACAACCCGTATACACCTCGAACGAACGACGACCAGATGGCATTGGAAGAGTTTCGGATGGGTCTGACAGGAAGGATAG  TTACAATGGCTCCTCCGATCTCCAGCTGGAGCGCATGAACGTCTACTTCAACGAGGTGCGTACCTCAAATTTTTCAGCCGCTATAAAAACGCTTTGCAAGTCCTGACCGCTTCTCCAGGCCAGCGGAAACAAGTATGTCCCTCGTGCCGTCCTCGTCGATCTTGAGCCCGGTACCATGGACGCCGTCCGTGCCGGTCCCTTCGGTCAGCTGTTCCGTCCCGACAACTTCGTTTTCGGCCAGTCCGGTG | >MK451505.1 Aspergillus pseudonomiae strain CMV002B6 calmodulin gene, partial cds  GCCTTCTCCCTATTCGTAAGTCATTATCATCGTTCGCGAAAATCGGTTTTGATTAGCTTTTATTCGAACACAAGCTGACTTGGCTTTTCTTGGGTTCCCAATAGGACAAGGATGGTGATGGTTAGTACAAGTCATTTTATTCAATCTCTCTTCAACTTCGACCAATATCCTTTAGCCGCCATAATTTTACCCAATTTCTGTTCGATCGGTTATAGTCTTGGCGTTGATAAATTGACATGATATGCAGGCCAGATCACCACCAAGGAGTTGGGCACTGTCATGCGCTCTCTGGGCCAAAACCCCTCTGAGTCGGAACTCCAGGACATGATTAACGAGGTTGACGCCGATAACAATGGCACCATTGACTTCCCTGGTACGAGACGGCTTCCGTACGATACACACATGAAATAGTTGTTAATGTTCAATCAGAGTTCCTTACGATGATGGCGAGAAAGATGAAGGATACCGACTCTGAGGAGGAGATCCGGGAGGCTTTCAAGGTTTTCGACCGCGATAACAACGGCTTTATCTCTGCTGCCGAACTGCGCCACGTCATGACCTCTATCGGCGAGAAGCTTACCGATGATGAAGTTGATGAGATGATCCGCGAGGCGGATCAGGATGGTGACGGCCG |
| Aspergillus flavus | >NR_111041.1 Aspergillus flavus ATCC 16883 ITS region; from TYPE material  TCCGTAGGTGAACCTGCGGAAGGATCATTACCGAGTGTAGGGTTCCTAGCGAGCCCAACCTCCCACCCGTGTTTACTGTACCTTAGTTGCTTCGGCGGGCCCGCCATTCATGGCCGCCGGGGGCTCTCAGCCCCGGGCCCGCGCCCGCCGGAGACACCACGAACTCTGTCTGATCTAGTGAANTCTGAGTTGATTGTATCGCAATCAGTTAAAACTTTCAACAATGGATCTCTTGGTTCAGGCATCGATGAAGAACGCAGCGAAATGCGATAACTAGTGTGAATTGCAGAATTCCGTGAATCATCGAGTCTTTGAACGCACATTGCGCCCCCTGGTATTCCGGGGGGCATGCCTGTCCGAGCGTCATTGCTGCCCATCAAGCACGGCTTGTGTGTTGGGTCGTCGTCCCCTCTCCGGGGGGGACGGGCCCCAAAGGCAGCGGCGGCACCGCGTCCGATCCTCGAGCGTATGGGGCTTTGTCACCCGCTCTGTAGGCCCGGCCGGCGCTTGCCGAACGCAAATCAATCTTTTTCCAGGTTGACCTCGGATCAGGTAGGGATACCCGCTGAACTTAAGCATATCAATAAGCGGAGGA | >EU982070.1 Aspergillus flavus strain ATHUM 5015 BenA (benA) gene, partial cds  TGGTATGTCTCAATGCCTTCGAGTTAGTATGCTTTGGACCAAGGAACTCCTCAAAAGCATGATCTCGGAT  GTGTCCTGTTATATCTGCCACATGTTTGCTAACAACTTTGCAGGCAAACCATCTCTGGCGAGCACGGCCT  TGACGGCTCCGGTGTGTAAGTACAGCCTGTATACACCTCGAACGAACGACGACCATATGGCATTAGAAGT  TGGAATGGATCTGACGGCAAGGATAGTTACAATGGCTCCTCCGATCTCCAGCTGGAGCGTATGAACGTCT  ACTTCAACGAGGTGCGTACCTCAAAATTTCAGCATCTATGAAAACGCTTTGCAACTCCTGACCGCTTCTC  CAGGCCAGCGGAAACAAGTATGTCCCTCGTGCCGTCCTCGTTGATCTTGAGCCTGGTACCATGGACGCCG  TCCGTGCCGGTCCCTTCGGTCAGCTCTTCCGTCCCGACAACTTCGTTTTCGGCCAGTCCGGTGCTGGTAA  CAACTGG | >MN271387.1 Aspergillus flavus strain PEDX3 calmodulin (CaM) gene, partial cds  CAAAGAGGCCTTCTCCCTATTCGTAAGTAGTTATCGTCGTTCGTGAAAATTGGTTTTGTTAGTCGTCATGATTTGAACACAAGCTGACTTGGCTTTCTTGGGTTTCCTATAGGACAAGGACGGTGATGGTTAGTACAGTTTATTTTATTCATTCTCCCTTCAAATGCGACCAATATGTTTTAGCCGCCATAATTTTATCCAGTTTCTGTTCGATCGGCTGAAGTCTTGGCATTGATGAATTGACTTGATATGCAGGCCAGATCACCACCAAGGAGTTGGGCACTGTCATGCGCTCTCTGGGCCAAAACCCCTCTGAGTCGGAACTCCAGGACATGATTAACGAGGTTGACGCCGACAACAATGGCACCATTGACTTCCCTGGTACGAGACGGCTTCCGTACGATTCATAAATGAAATAGCTGTTAATGTTCAAATAGAGTTCCTGACGATGATGGCGAGAAAGATGAAGGATACCGACTCTGAGGAGGAGATCCGGGAGGCTTTCAAGGTTTTCGACCGCGATAACAACGGCTTCATCTCCGCTGCCGAATTGCGCCACGTTATGACCTCTATCGGA |
| Aspergillus alliaceus | > Aspergillus alliaceus strain CBS 536.65 ITS  TCGTGGAGCCCACCTCCCACCCGTGTATACTGTACCTTCGTTGCTTCGGCGGGCCCGCCGTCATGGCCGCCGGGGGGCTTCTGCCCCCGGGCCCGCGCCCGCCGGAGACACATGAACTCTGTCTGATCTAGTGAAGTCTGAGTTGATTGTCACACAATCAGTTAAAACTTTCAACAATGGATCTCTTGGTTCCGGCATCGATGAAGAACGCAGCGAAATGCGATAACTAATGTGAATTGCAGAATTCCGTGAATCATCGAGTCTTTGAACGCACATTGCGCCCCCTGGTATTCCGGGGGGCATGCCTGTCCGAGCGTCATTGCTGCCCATCAAGCACGGCTTGTGTGTTGGGTCCTCGTCCCCCCCGGGGGACGTGCCCGAAAGGCAGCGGCGGCACCGCGTCCGGTCCTCGAGCGTATGGGGCTTTGTCACCCGCTCTGCAGGCCCGGCCGGCGCTGGCCGACGCGAAAGCAACCATTTTTTCTCCAGGTTGACCTCGGAT | >OU641278.1 Aspergillus alliaceus partial tubb gene for beta tubulin  TGGTAACCAAATCGGTGCTGCTTTCTGGTATGTTTCAATTCTCAGATCAAGTCTGGGACAAAAGATCCCTACCGATGCATGGGATCTAATGCGTCCCATTACTTCTGCCACGTGTTTGCTAACGGTTTTACAGGCAGACCATTTCTGGCGAGCACGGCCTTGACGGCTCCGGTGTGTAAGTACAACCCGTGTACATCTCGAACGAAGGAC  AATCCGTTGGCGATGGAAGGGTCTGAAAGGGTCTGACGGGAAGGATAGTTACAATGGCTCCTCCGACCTCCAGCTGGAGCGCATGAACGTCTACTTCAACGAGGTGCGTACCTCAGATTTTGCAGCCTCCCTAGAAACGCCGTGCAGGCCCTGACCACTTCTCCAGGCTAGTGGAAACAAGTATGTTCCTCGTGCCGTCCTCGTCGATCT  TGAGCCCGGTACCATGGACGCCGTCCGCGCAGGTCCCTTCGGTCAGCTTTTCCGTCCCGACAACTTCGTTTTTGGCCAGTCCGGTGCTGGTAACAACTGGGCCAAGGGTCACTACACTGAGGGTA | >OU641279.1 Aspergillus alliaceus partial CaM gene for calmodulin  CCTATTCGTAAGTTGTTCTACCTATTCGCGAAAACTGATATTTTCTGATGGTCTTGGTGGAATGCATACTGACTTGAGTTTTCTTGGGCTCCTAATAGGACAAGGATGGTGATGGTTAGTACATCATGTTCCATAAAACCCCCCTTCTAGTGCGACCGACAGTTTTCAGCCACTATAATCGTCTCCATATTTTTTATTGTTCGATCGGCTGAAGTCTTGGCGTTGATAAATTGACTCGATATGCAGGCCAGATCACCACCAAGGAGTTGGGCACTGTGATGCGCTCTCTGGGCCAGAACCCTTCTGAGTCGGAACTCCAGGATATGATCAACGAGGTTGATGCCGATAACAATGGCACCATCGACTTCCCTGGTACGCGAGGGCTTTCCTACGCCTCACAGACAAAGAAATTCTTATTAACGTTCGATTAGAGTTCCTTACGATGATGGCCAGAAAGATGAAGGATACCGACTCTGAGGAGGAGATCCGGGAGGCTTTCAAGGTTTTCGACCGTGATAACAACGGCTTTATCTCCGCCGCGGAGCTGCGCCACGTCATGACCTCCATCGGTGAGAAACTTACCGATGATGAAGTTGATGAGATGATCCGCGAGGCGGATCAGGACGGTGATGGCCGGATCGATTGTACGTTGAGAACAACTCCCCATTTCTTTTACCCGCTGAGGATGAATGTGGATGTGAATCGCGGATACTGACCGGCTCTAGACAACGAGTTCGTC |
| Aspergillus hiratsukae | > Aspergillus hiratsukae strain CBS 294.93 ITS  ATCATTACCGAGTGCTGGGCCCTCTGGGTCCAACCTCCCACCCGTGTCTATTGTACCTTGTTGCTTCGGCGGGCCCGCCGTTTTCGAACGGCCGCCGGGGAGGCCTCGCGCCCCCGGGCCCGCGCCCGCCGAAGACCCCAACATGAACGCTGTTCTGAAAGTATGCAGTCTGAGTTTGATTATCATAATCAGTTAAAACTTTCAACAACGGATCTCTTGGTTCCGGCATCGATGAAGAACGCAGCGAAATGCGATAAGTAATGTGAATTGCAGAATTCAGTGAATCATCGAGTCTTTGAACGCACATTGCGCCCCCTGGTATTCCGGGGGGCATGCCTGTCCGAGCGTCATTGCTGCCCTCAAGCACGGCTTGTGTGTTGGGCCCCCGTCCCCGGTTCTCCCCGGGGACGGGCCCGAAAGGCAGCGGCGGCACCGCGTCCGATCCTCGAGCGTATGGGGCTTTGTCACCCGCTCTGTAGGCCCGGCCGGCGCCAGCCGACACCCCAACTTTATTTTTCTAAGGTTGACC | >MW217709.1 Aspergillus hiratsukae strain 19-2832 beta-tubulin (BenA) gene, partial cds  TCATGCTTGGATGATGGATATTAGGACCAGCCAGCATCCTCGATCGGTTCTAGTTGCTGTCATGGGTATAGCTAACAAATTTCCAGGCAGACCATCTCTGGTGAGCACGGCCTTGACGGCTCTGGCCAGTAAGTTCGACCTTTATCCTCCCAATTGAGAAAGCGGGGGAAACACGAAAGGCAAGCAGGAAGAGAACGCGTCTCTGATGGG  ATAATAGCTACAATGGCTCCTCCGATCTCCAGCTGGAGCGTATGAACGTCTACTTCAACGAGGTGCGTGGATGAAACTCTCGACTCTTAACTACTTCGGCAACATCTCACGATCTGACTCGCTACTAGGCCAACGGTGACAAGTATGTTCCTCGTGCCGTTCTGGTCGATCTCGAGCCCGGTACCATGGACGCTGTCCGTGCCGGCCCCT  TCGGCGAGCTCTTCCGTCCCGACAACTTCGTCTTCGGCCAGTCTGGTGCTGGTAACAACTGGGCCAAGGGTCACTC | >MK451422.1 Aspergillus hiratsukae strain CMV012G1 calmodulin gene, partial cds  GCCTTCTCTCTCTTCGTAAGTGAACTGTTCTAGTCCCTAGTCGTTGCATAAGTGGAATTGCCACACTGTTGAGGGTGTGCGCTCACATGAGATTCGACCTATAGGACAAGGATGGTGATGGTTAGTGACCTTTTTCCGCTCCTCGAACGTTGGCTTCCGTGCGACCGTGTTCAAATGCCGACGTCCAATATTCCGCAATGATCTATCGATACTGATAATATCTATGTTTGACTCTTAGGCCAGATCACCACCAAGGAATTGGGCACTGTCATGCGCTCGCTGGGCCAGAACCCTTCCGAGTCAGAGCTGCAAGATATGATCAACGAGGTGGATGCTGACAACAATGGCACCATCGATTTCCCTGGTATGCGATGCATCTTGCGTGACGTGGCTGGGAATGAGGAGAACAATCATTTCAGA  ATTCTAACCCGTATTCAGAATTCCTTACTATGATGGCTCGGAAGATGAAGGATACCGACTCCGAAGAGGAAATCCGGGAAGCCTTCAAGGTCTTCGACCGCGACAACAACGGTTTCATTTCCGCTGCGGAGCTGCGCCACGT |
| Aspergillus amoenus | > Aspergillus amoenus strain DTO:269-A7 ITS  TGAGTGCGGGCTGCCTTCGGGCGCCCAACCTCCCACCCGTGACTACCTAACACTGTTGCTTCGGCGGGGAGCCCTCTCGGGGGCGAGCCGCCGGGGACTACTGAACTTCATGCCTGAGAGTGATGCAGTCTGAGTCTGAATATAAAATCAGTCAAAACTTTCAACAATGGATCTCTTGGTTCCGGCATCGATGAAGAACGCAGCGAACTGCGATAAGTAATGTGAATTGCAGAATTCAGTGAATCATCGAGTCTTTGAACGCACATTGCGCCCCCTGGCATTCCGGGGGGCATGCCTGTCCGAGCGTCATTGCTGCCCATCAAGCCCGGCTTGTGTGTTGGGTCGTCGTCCCCCCCGGGGGACGGGCCCGAAAGGCAGCGGCGGCACCGTGTCCGGTCCTCGAGCGTATGGGGCTTTGTCACCCGCTCGATTTAGGGCCGGCCGGGCGCCAGCCGACGTCCAACCATTTTTCTTCA | >LC589362.1 Aspergillus amoenus NRRL 226 BenA gene for beta-tubulin, partial cds  TGGTGCGTCGAAAATTTCATCCATTTCAGATGGTATTTCCTTTGGTGCTTATTGCTAACGACCCTATAGGCAGACCATCTCCGGCGAGCACGGCCTTGATGGCTCCGGTGTGTGAGTACAACCCGTCCAGGACTCGATCAAAACACGACGGAACACAACCCCTGATATAATACAGTTACAATGGTACCTCCGACCTCCAGCTCGAGCGTATGAACGTCTACTTCAACGAGGCCAGCGGCAACAAGTACGTTCCCCGTGCCGTTCTCGTCGATCTCGAGCCCGGTACTATGGACGCCGTCCGTGCCGGTCCCTTCGGTCAGCTCTTCCGTCCCGACAACTTCGTCTTTGGCCAGTCCGGTGCTGGTAACAACTGG | >MK451308.1 Aspergillus amoenus strain CMV008E2 calmodulin gene, partial cds  GCCTTCTCCCTATTTGTAAGTGCCATTGATCCCATCGGTATCAGTTATGTGGCTTTTCCGACAAGGCACTAATTTATTCTGCGCTTAACAGGACAAGGATGGCGATGGTTAGTGTGCTCCCCGCGATAACTTAGTCGCATACGCCCAATGTCGACATCATAATTCTATGTAATTGGATATTGACACACTATTCAGGCCAGATCACTACCAAGGAGCTCGGCACCGTGATGCGCTCGCTCGGCCAGAACCCCTCGGAGTCTGAGCTTCAGGACATGATCAACGAAGTCGATGCTGACAACAACGGCACCATTGATTTCCCGGGTACGTGGCCTTGCAAAAATAAAAGTTACCGTCAAAGCGAACAACTGTTAACTGTCAAACAGAGTTCCTCACGATGATGGCTAGAAAGATGAAGGACACCGACTCCGAGGAGGAAATTCGGGAGGCATTCAAGGTTTTCGACCGTGATAACAATGGCTTCATCTCGGCTGCCGAGCTGCGCCACGT |
| Aspergillus lentulus | >NR_135407.1 Aspergillus lentulus NRRL 35552 ITS region; from TYPE material  AAGGATCATTACCGAGTGAGGGCCCTCTGGGTCCAACCTCCCACCCGTGTCTATCGTACCTTGTTGCTTCGGCGGGCCCGCCGTTTCGACGGCCGCCGGGGAGGCCTCGCGCCCCCGGGCCCGCGCCCGCCGAAGACCCCAACATGAACGCTGTTCTGAAAGTATGCAGTCTGAGTTGATTATCATAATCAGTTAAAACTTTCAACAACGGATCTCTTGGTTCCGGCATCGATGAAGAACGCAGCGAAATGCGATAAGTAATGTGAATTGCAGAATTCAGTGAATCATCGAGTCTTTGAACGCACATTGCGCCCCCTGGTATTCCGGGGGGCATGCCTGTCCGAGCGTCATTGCTGCCCTCAAGCACGGCTTGTGTGTTGGGCCCCGTCCCCTCCCCCGGGGACGGGCCCGAAAGGCAGCGGCGGCACCGCGTCCGGTCCTCGAGCGTATGGGGCTTTGTCACCCGCTCTGTAGGCCCGGCCGGCGCCAGCCGACACCCAACTTTATTTTCTAA | >MZ062543.1 Aspergillus lentulus isolate BMU09589 beta-tubulin gene, partial cds  TGGTATGTCTCCACCTCAATGCTAGGATGATGGGAGACTGGGACCTGTCATCTTAGCAGGCTGTCCCCATGTGTTCAGCTTCGCTGTCATGAGTATCAGCTAACAAATCTACAGGCAGACCATCTCTGGTGAGCACGGCCTTGACGGCTCTGGCCAGTAAGTTCGACCTATATCCTCCCAATTGAGAAAGCGGCGGAAACACGTAAGGCAAGGAAGAAGAGGACGCGTGTCTGACGGGGATAATAGCTACAATGGCTCCTCCGATCTTCAGCTGGAGCGTATGAACGTCTATTTCAACGAGGTGCGTGGATGAAACTCTCGACTCTACACTCCTTTGACAACATCTTACGGTCTGACTCGCTACTAGGCCAACGGCGACAAGTATGTTCCTCGTGCCGTCCTGGTCGATCTCGAGCCCGG  TACCATGGACGCTGTCCGTGCCGGTCCCTTCGGCGAGCTCTTCCGTCCCGACAACTTCGTCTTCGGCCAGTCTGGTGCTGGTAACAACTGG | >KJ775275.1 Aspergillus lentulus strain DTO:179-A4 calmodulin (cmdA) gene, partial cds  CTCTTCGTAAGTGAACTGTCCACCTCACTAGTCGTCGCAGAAGAGGAATTTCCACAACCTTGAGGGTGTGCGCTCACACGAGATTCGACCTATAGGACAAGGATGGTGATGGTTAGTGACCTTTTTTCCGCTCCTCGGACGTCTGCTTGCATGCGACCATGTTCAAATGCCGACTTACAATACCCGGAAATGATCCATCAATACTGATAATATCTATGTTTGACTATTAGGCCAGATCACCACCAAGGAATTGGGCACTGTGATGCGCTCCCTGGGCCAGAACCCTTCCGAATCAGAGCTGCAAGATATGATCAACGAGGTGGATGCTGACAACAACGGCACCATCGATTTCCCTGGTATGCGATGCATTTGGTGTGACCTCGGGAGGGAGGAGAACAATCATTAACTCATTAACTCGTATTCAGAATTCCTTACTATGATGGCTCGGAAGATGAAGGACACCGACTCTGAAGAGGAAATCCGGGAAGCTTTCAAGGTCTTCGACCGCGACAACAACGGTTTCATCTCCGCTGC |
| Aspergillus nomiae | >NR_121218.1 Aspergillus nomiae NRRL 13137 ITS region; from TYPE material  AAGGATCATTACCGAGTGTAGGGTTCCTAGCGAGCCCAACCTCCCACCCGTGTTTACTGTACCTTAGTTGCTTCGGCGGGCCCGCCGCAAGGCCGCCGGGGGGCATCCGCCCCCGGGCCCGCGCCCGCCGGAGACACCACGAACTCTGAACGATCTAGTGAAGTCTGAGTTGATTGTATCGCAATCAGTTAAAACTTTCAACAATGGATCTCTTGGTTCCGGCATCGATGAAGAACGCAGCGAAATGCGATAACTAGTGTGAATTGCAGAATTCCGTGAATCATCGAGTCTTTGAACGCACATTGCGCCCCCTGGTATTCCGGGGGGCATGCCTGTCCGAGCGTCATTGCTGCCCATCAAGCACGGCTTGTGTGTTGGGTCGTCGTCCCCCCCTCCGGGGGGGGACGGGCCCTAAAGGCAGCGGCGGCACCGCGTCCGATCCTCGAGCGTATGGGGCTTTGTCACCCGCTCTGTAGGCCCGGCCGGCGCTTGCCGAACGCAAAACAACCATTCTTTCCA | >MK450926.1 Aspergillus nomiae strain CMV002B2 beta-tubulin gene, partial cds  TGGTATGTCTCGATACCTCCGAGTCAATATGCTTTGGACCTAGGGCCTCAGCAGAAACATGAGCTCGGATATATCATCTCGTGTCTGACACGTGTTTGCTAATACCTTTGCAGGCAGACCATCTCCGGCGAGCACGGCCTCGACGGCTCCGGTGTGTAAGTACAACCCGCGTACACCTCGAATGAACGACGATCAGATGGCATTGGAAGA  GTTTTGGATGGGTCTGACGGGAAGGATAGTTACAATGGCTCCTCCGATCTCCAGCTGGAGCGCATGAACGTCTACTTCAACGAGGTGCGTACCTCAAATTTTTCAGCCGCTATAAAAACGCTTTGCAAGTCCTGACCGCTTCTCCAGGCCAGCGGAAACAAGTATGTCCCTCGTGCCGTCCTCGTCGATCTTGAGCCCGGTACCATGGAC  GCCGTCCGTGCCGGTCCCTTCGGTCAGCTGTTCCGTCCCGACAACTTCGTTTTCGGCCAGTCCGGTGCTG  GTAACAACTGGGCCAAGGGTC | >MK451473.1 Aspergillus nomiae strain CMV002B2 calmodulin gene, partial cds  GCCTTCTCCCTATTCGTAAGTCATTATCATCGTTCGCGAAAATCGGGTTTGATTAGCCTTTATTCGAACACAAGCTGACTTGGCTTTTCTTGGGTTCCCAATAGGACAAGGATGGTGATGGTTAGTACAAGTCATTTTATTCAATCTCTCTTCAACTTCGACCAATATCCTTTAGCCGCCATAATTTTACCCATTTTCTGTTCGATCGGTTGTAGTCTTGGCGTTGATAAATTGACATGATATGCAGGCCAGATCACCACCAAGGAGTTGGGCACTGTCATGCGCTCTCTGGGCCAAAACCCCTCTGAGTCGGAACTCCAGGACATGATTAACGAGGTTGACGCCGATAACAATGGCACCATTGACTTCCCTGGTACGAGACGGCTTCCGTACGATACACACATGAAATAGTTGTTAATGTTCAATCAGAGTTCCTTACGATGATGGCGAGAAAGATGAAGGATACCGACTCTGAGGAGGAGATCCGGGAGGCTTTCAAGGTTTTCGATCGCGATAACAACGGCTTTATCTCTGCTGCCGAACTGCGCCACGTCATGACCTCTATCGGCGAGAAGCTTACCGATGATGAAGTTGATGAGATGATCCGCGAGGCGGATCAGGATGGTGACGGCCG |
| Aspergillus ochraceus | >NR_077150.1 Aspergillus ochraceus NRRL 398 ITS region; from TYPE material  TCCGTAGGTGAACCTGCGGAAGGATCATTACTGAGTGAGGGTCCCTCGGGGCCCAACCTCCCACCCGTGTATACCGTACCTTGTTGCTTCGGCGAGCCCGCCCCCTTTTTCTTTTAGGGGGCACAGCGCTCGCCGGAGACACCAACGTGAACACTGTCTGAAGTTTTGTCGTCTGAGTCGATTGTATCGCAATCAGTTAAAACTTTCAACAATGGATCTCTTGGTTCCGGCATCGATGAAGAACGCAGCGAAATGCGATAATTAATGTGAATTGCAGAATTCAGTGAATCATCGAGTCTTTGAACGCACATTGCACCCCCTGGTATTCCGGGGGGTATGCCTGTCCGAGCGTCATTGCTGCCCTCAAGCACGGCTTGTGTGTTGGGTCGTCGTCCCCCCCCAGGGGGACGGGCCCGAAAGGCAGCGGCGGCACCGCGTCCGGTCCTCGAGCGTATGGGGCTTTGTCACCCGCTCTTGTAGGCCCGGCCGGCTGCTGGCCGACGCTGAAAAGCAACCAACTATTTTTCCAGGTTGACCTCGGATCAGGTAGGGATACCCGCTGAACTTAAGCATATCAATAAGCGGAGGAAAAGAAACCAACCGGGATTGCCTC | >KJ767724.1 Aspergillus ochraceus strain LEMI966 beta-tubulin (BenA) gene, partial cds  TGGTAACCAGATAGGTGCTGCTTTCTGGTAAGTCTACACGTTGGCATTGGCAGTCTGGGCTATCTGAGATCATCTTTGATACCTTGGCGCTTGTGACGCTCAATCCTAGATACTTGTTTACTGATAGGTGAATAGGCAAAACATCTCTGGCGAGCACGGCCTTGACGGCGCCGGTGTGTAAGTACATCCCGCGTTTACACCTATCGAAATCAGAATCGAAGAGAGAAGAAATAAACGACCATGGTGAGAGATTGATTGTCTGATGGGATGAACAGTTACAATGGCTCCTCCGACCTTCAGCTGGAGCGCATGAACGTCTACTTCAACGAGGTTCGTTGCCCGAAAATTTTCTATCTCCCTTCACCTATCCGAAACGCCCCGTACAAAGCTCTAACCCACGCTCTCTTCATTTTTTAGGCTTCCGGTGGCAAGTATGTTCCCCGTGCCGTTCTGGTCGATCTTGAGCCCGGTACCATGGACGCTGTCCGTGCCGGTCCCTTCGGTCAGCTTTTCCGCCCCGACAACTTCGTCTTCGGCCAGTCTGGTGCCGGTAACAACTGGGCCAAGGGTCACTACACCGAG | >MK451476.1 Aspergillus ochraceus strain CMV007B6 calmodulin gene, partial cds  GCCTTCTCCCTGTTCGTAAGTGATCCATTCTTTCTGATGTAATGATGGGGACACGAAGCGATCTTGCTTTGTTTTTTACGCCCTCAGCCTTATGGGAATATCCCAAGCTCACATGAGATTTTGCCTCTTCACAGGACAAGGATGGCGATGGTTAGTGCTATTCCGATTCCCTTCCAGTTCGTCGACTCACGCGACCGGCTATTTTCCGTCGACATGGATCTTTTTTTTCACCCTGTTGCTTCCGCCGACCGATTTGCGACCAGGACGCTAATTTTGTGAATTCACGATGCAGGCCAGATCACCACCAAGGAGTTGGGCACTGTTATGCGCTCGCTGGGCCAGAACCCCTCCGAGTCTGAGTTGCAGGACATGATCAACGAGGTTGACGCCGACAACAATGGCACCATTGATTTCCCCGGTATGCGATGATGGACACACCGATATCCGGAAAGGGAAACGATCCTGAAACCCAGCTGCTAACCTCACGCAGAGTTCCTGACGATGATGGCCCGAAAGATGAAGGATACCGATTCTGAGGAGGAAATCCGGGAAGCTTTCAAGGTCTTCGATCGCGATAACAACGGTTTCATCTCGGCCGCGGAGCTGCGCCACGT |
| Aspergillus cristatus | >NR_135341.1 Aspergillus cristatus NRRL 4222 ITS region; from TYPE material  AAGGATCATTACCGAGTGCGGGCCCTCTGGGTCCAACCTCCCATCCGTGTCTATCTGTACCCTGTTGCTTCGGCGTGGCCACGGCCCGCCGGAGACTAACATTTGAACGCTGTCTGAAGTTTGCAGTCTGAGTTTTTAGTTAAACAATCGTTAAAACTTTCAACAACGGATCTCTTGGTTCCGGCATCGATGAAGAACGCAGCGAAATGCGATAATTAATGTGAATTGCAGAATTCAGTGAATCATCGAGTCTTTGAACGCACATTGCGCCCCCTGGTATTCCGGGGGGCATGCCTGTCCGAGCGTCATTGCTGCCCTCAAGCACGGCTTGTGTGTTGGGCTTCCGTCCCTGGCAACGGGGACGGGCCCAAAAGGCAGTGGCGGCACCATGTCTGGTCCTCGAGCGTATGGGGCTTTGTCACCCGCTCCCGTAGGTCCAGCTGGCAGCTAGCCTCGCAACCAATCTTTTTAACCA | >EF651914.1 Aspergillus cristatus isolate NRRL 4222 beta-tubulin gene, partial cds  TGGTATGTCTGCATTATAATCAGAGTCGGATTTAGTGATATACTAACAGTATCACAGGCAGACTATCTCCGGCGAGCACGGTCTCGACGGCTCTGGTGTGTAAGTACAGTCGGGTCTCCGGGATGGATGCGTATCGGATATGGATATCTAAATGGATTGCAGCTACAATGGCTCCTCCGACCTCCAGTTGGAGCGTATGAACGTCTACTTCAACGAGGTTTGCTTAATTCATTCGTGTTTGTGTGGAAAACAGTTCTGACAGTGACAGGCCTCCAACAACAAATATGTCCCCCGTGCCGTCCTCGTCGACCTTGAGCCCGGTACCATGGACGCCGTCCGTGCCGGTCCCTTCGGTCAGCTCTTCCGTCCCGACAACTTCGTCTTCGGTCAGTCCGGTGCCGGTAACAACTGG | >LT671062.1 Aspergillus cristatus partial CaM gene for calmodulin, strain CGMCC 3.06081  CCCCGTCGGGCGTTGAGGACAGTAAACTGACCACGATTTTCGCATTTCTACAGGACAAGGATGGCGATGGTTAGTGATCCCGCTCTACTCTGCATCCAATGCCTCTTTTACCCCACCATTCGATTGGTTCCGACCGTGATATCATATTCGTTGTTGAAATAATACCTTTCCGGTTAACGACATAATACTGATGGATCTCCGCGATTACAGGCCAGATCACCACCAAGGAGCTGGGTACCGTTATGCGCTCGCTGGGCCAGAACCCGTCCGAGTCGGAGTTGCAGGACATGATCAACGAGGTTGACGCTGACAACAACGGCACCATCGATTTCCCTGGTATGCGATCGTCCCGACATAAAAGGACCCGTAACAAGATAGGCGATTCTGATCATACTAGAATTCCTTACCATGATGGCACGG  AAGATGAAAGACACCGACTCCGAGGAGGA |
| Aspergillus ibericus | >NR_119514.1 Aspergillus ibericus IMI 391429 ITS region; from TYPE material  CCGAGTGCGGGTCCTTTGGGCCCAACCTCCCACCCGTGTCTATTGTACCCTGTTGCTTCGGCGGGCCCGCCGCTTGTCGGCCGCCGGGGGGGCATCTCTGCCCCTCGGGCCCGCGCCCGCCGGAGACACCAACACGAACACTGTCTGAAATCGTGAAGTCTGAGTCGATTGTTTTCAATCAGTTAAAACTTTCAACAATGGATCTCTTGGTTCCGGCATCGATGAAGAACGCAGCGAAATGCGATAACTAATGTGAATTGCAGAATTCAGTGAATCATCGAGTCTTTGAACGCACATTGCGCCCCCTGGCATTCCGGGGGGCATGCCTGTCCGAGCGTCATTGCTGCCCTCAAGCCCGGCTTGTGTGTTGGGTCGCCGTCCCCCTTTCCGGGGGGACGGGCCCGAAAGGCAGCGGCGGCACCGCGTCCGATCCTCGAGCGTATGGGGCTTTGTCACATGCTCTGTAGGATTGGCCGGCGCCTGCCGACAACTCCAACCTTTTTTTTCCA | >MH614574.1 Aspergillus ibericus voucher IHEM 23498 beta-tubulin (benA) gene, exons 3 through 6 and partial cds  AGTGTGTAAGTGCGACCATGTCTTGGGATTGTTGTCCAAGTGTCATGCTTGGTGTCCAGCGGCTAAACAACGATGGCGAATAGGGTAACCAAATTGGTGCTGCTTTCTGGTATGTCTCACCGTCACTGGACTTGGGATGGGATCTCTCAGGCTTTCCAAGCATGAGGCTAGATACCCATTGATACTCTCACTCAAGGATTAATCTAACAAATCAACAGGCAGACCATCTCTGGTGAGCACGGCCTCGATGGCGCTGGTGTGTAAGTACACCCTCTTCACAGCTCTCAATCGATCCACATGAGGAACGAGTCAGGTCTCCTGACATGGAGGATAGTTACAATGGTACCTCCGATCTCCAGCTGGAGCGCATGAACGTCTACTTCAACGAGGTGGGTATCAACCAGCCCCTGGGTGTACCACGTGTACATGTGGTGTCCCAATTTCTGACCCCTTCACCAGGCTAGCGGCAACAAGTATGTTCCCCGTGCTGTCCTTGTCGACCTTGAGCCCGGTACCATGGACGCCGTCCGTGCCGGTCCTTTCGGCCAGCTTTTCCGCCCCGACAACTTCGTCTTCGGCCAGTCCGGTGCTGGTAACAACTGGGCCAAGGGT | >AJ971806.1 Aspergillus ibericus partial cdl gene for calmodulin, exons 2-3, strain ITEM 6600  TCTTTGTAAGTTCCCCCTGAATGACTTCCGACCAGCGAAAGCGATAGGTTACTTCACAGAAATACATACTCATGTGCTTTGATCCAATAGGACAAGGATGGCGATGGTGGGTGGAATTCTGTCCCTTTCTCTGGCGACTGAACTGACCGCGGGGTTGCCGTCTTCCGACCGACTTGAACTTAATACTGATGTGACTTGGGGAATAGGCCAGATCACCACCAAGGAGCTGGGCACTGTCATGCGCTCGCTCGGCCAGAACCCCTCCGAGTCTGAGCTCCAGGACATGATCAACGAGGTTGATGCTGACAACAACGGCACGATCGACTTCCCCGGTATGTGGCGTGTTGATATTTATAGGAAGAGGGAATGCGGGCCAGATATTAACTTCTGCCAGAATTCCTTACCATGATGGCTCGTAAGATGAAGGACACCGACTCCGAGGAGGAGATCCGCGAGGCATTCAAGGTCTTCGACCGCGATAACAACGGTTTCATCTCCGCTGCGGAGCTCCGTCATGTCATGACTTCCATCGGTGAGAAGCTCACCGACGACGAGGTCGATGAGATGATCCGTGAGGCGGACCAGGACGGTGACGGCCGCATTGACTGTACGTTTCTCATTCTTAACCATACGCATTTAATATACTAACCTGGTG |
| Aspergillus tubingensis | >NR_131293.1 Aspergillus tubingensis NRRL 4875 ITS region; from TYPE material  CGAGTGCGGGTCCTTTGGGCCCAACCTCCCATCCGTGTCTATTATACCCTGTTGCTTCGGCGGGCCCGCCGCTTGTCGGCCGCCGGGGGGGCGCCTTTGCCCCCCGGGCCCGTGCCCGCCGGAGACCCCAACACGAACACTGTCTGAAAGCGTGCAGTCTGAGTTGATTGAATGCAATCAGTTAAAACTTTCAACAATGGATCTCTTGGTTCCGGCATCGATGAAGAACGCAGCGAAATGCGATAACTAATGTGAATTGCAGAATTCAGTGAATCATCGAGTCTTTGAACGCACATTGCGCCCCCTGGTATTCCGGGGGGCATGCCTGTCCGAGCGTCATTGCTGCCCTCAAGCCCGGCTTGTGTGTTGGGTCGCCGTCCCCCTCTCCGGGGGGACGGGCCCGAAAGGCAGCGGCGGCACCGCGTCCGATCCTCGAGCGTATGGGGCTTTGTCACATGCTCTGTAGGATTGGCCGGCGCCTGCCGACGTTTTCCAACCATTTTTTCCA | >AY585528.1 Aspergillus tubingensis strain CBS 12652 beta tubulin gene, partial cds  TGGTACGTATTCACTGCCACTGGATTGGGGATGGAACATCATCTCTCAAGCTATCTTAGCTTGAGTTCAGATGTTATCCATCGGGTATATAGCTATCGGGTTAAGAACACGTCTAACAACTCAACAGGCAGACCATCTCTGGCGAGCACGGCCTTGACGGCTCCGGTGTGTAAGTACAACTTTTTCACACCTCTCAATTGGTCAACAATGTGGAAAGGATTGGGTTTCCTGACGCGCAGGATAGTTACAATGGCACCTCCGACCTCCAGCTGGAGCGCATGAACGTCTACTTCAACGAGGTTAGATCACACCGTCCCTGAGTTTTTTCACGACAATATCATCAATGTCCTGACCACTTCAGCAGGCTAGCGGTAACAAGTATGTCCCCCGTGCCGTCCTCGTCGATCTCGAGCCCGGTACCATGGACGCCGTCCGTGCCGGTCCCTTCGGCCAGCTCTTCCGCCCCGACAACTTCGTCTTCGGCCAGTCCGGTGCTGGTAACAACTGG | >FR751416.1 Aspergillus tubingensis partial caM gene for calmodulin, culture collection CCF<CZE_:2818, exons 1-5  GCTTCTCCCTCTTTGTGAGTGCTCCCTGAATGAACCCCCGATCACTCAAATTGATGTCCTATCTTAACCGGCTCATAATGCTAATGTATTTTCAAACTCAATAGGACAAGGATGGCGATGGTGGGTGGAATTCTGTCCCCTTCACGTTTTACCTGTAGCGCCCGATCCGACCGCGGGATTTCGACAGCTATTTCCCCCTTCGATCTGAATCATAATACTGATGTAATCTGGAAATAGGCCAGATCACCACCAAGGAGCTCGGCACTGTGATGCGCTCCCTCGGCCAGAACCCCTCCGAGTCTGAGCTTCAGGACATGATCAACGAGGTTGACGCTGACAACAACGGAACGATCGACTTCCCCGGTATGTGATAGATCTACGCCTGTAAGGCGGGAATGCCGTATGGATTGTGATTGACTTTTGCCGCCAGAATTCCTTACCATGATGGCTCGTAAGATGAAGGACACCGACTCCGAAGAGGAAATCCGCGAGGCTTTCAAGGTCTTCGACCGCGACAACAATGGTTTCATCTCCGCCGCGGAGTTGCGCCACGTCATGACCTCCATTGGCGAGAAGCTCACTGACGACGAAGTCGATGAGATGATCCGTGAGGCTGACCAGGACGGTGATGGCCGCATCGACTGTATGTTTCCCATTCCTGATATGCCCGTGATATGACATGCTAACTCTGCTACCAGACAACGAGTTCGTCCA |
| Aspergillus fischeri | >NR_137479.1 Aspergillus fischeri NRRL 181 ITS region; from TYPE material  AAGGATCATTACCGAGTGAGGGCCCTCTGGGTCCAACCTCCCACCCGTGTCTATCGTACCTTGTTGCTTCGGCGGGCCCGCCGTTTCGACGGCCGCCGGGGAGGCCTCGCGCCCCCGGGCCCGCGCCCGCCGAAGACCCCAACATGAACGCTGTTCTGAAAGTATGCAGTCTGAGTTGATTATCATAATCAGTTAAAACTTTCAACAACGGATCTCTTGGTTCCGGCATCGATGAAGAACGCAGCGAAATGCGATAAGTAATGTGAATTGCAGAATTCAGTGAATCATCGAGTCTTTGAACGCACATTGCGCCCCCTGGTATTCCGGGGGGCATGCCTGTCCGAGCGTCATTGCTGCCCTCAAGCACGGCTTGTGTGTTGGGCCCCCGTCCCCTCTCCCGGGGACGGGCCCGAAAGGCAGCGGCGGCACCGCGTCCGGTCCTCGAGCGTATGGGGCTTTGTCACCCGCTCTGTAGGCCCGGCCGGCGCCA  GCCGACACCCAACTTTATTTCTAAGGTTGACCTCGGATCAGGTAGGGATACCCGCTGAACTTAAGCATATCAATAAGCGGAGGAAAAGAAACCAACAGGGATTGCCTCAGTAACGGCGAGTGAA | >MK297582.1 UNVERIFIED: Aspergillus fischeri isolate NCE03 beta-tubulin-like (benA) gene, partial sequence  TGGTATGTCTTGACCTCAATTCTTGGATGACGGGAGATTGGGACCTGTCTTAGCAGGCTGTCCTCCATGGGTTCAGCTTCGCTGTCATGGGTATCAGCTAACAAATCTACAGGCAGACCATCTCTGGTGAGCACGGCCTTGACGGCTCTGGCCAGTAAGTTCGACCTATATCCTCCCAATTGAGAAAGCGGCGGAAACACGAAAGGAAGGAAGAAGAGGACGCGTGTCTGATGGGGTTAATAGCTACAATGGCTCCTCCGATCTCCAGCTGGAGCGTATGAACGTCTACTTCAACGAGGTGTGTGGATGAAACTCTCGACTCTATACTATTTCGGCAACATCTCACGATCTGACTCGCTACTAGGCCAACGGTGACAAGTATGTTCCTCGTGCCGTTCTGGTCGATCTCGAGCCTGGTACCATGGACGCTGTCCGTGCCGGTCCCTTCGGCGAGCTCTTCCGTCCCGATAACTTCGTCTTCGGCCAGTCTGGTGCTGGTAACAACTGG | >MK451362.1 Aspergillus fischeri strain CMV012A6 calmodulin gene, partial cds  TTCGTAAGTGAACTGTCCAAGTCCCTAGTCGTTGCATAGGAGGGATCTACTCCATATTGAGGGTGTGCGCTCACACGAGATTCGACCTATAGGACAAGGATGGTGATGGTTAGTGACCCTTTTTCCGCTCCTCGAACTTCGGCTTCAAATGCGATCATGTTCAAACGCCGACTTACATTATCCGGAAATGACCCGTCAGTACTGATAATA  TCTATGTTTGACTATTAGGCCAGATCACCACTAAGGAATTGGGCACTGTTATGCGCTCCCTGGGCCAGAACCCTTCCGAGTCAGAGCTACAAGATATGATCAACGAGGTGGATGCTGACAACAACGGCACCATCGATTTCCCTGGTATGCGATGCATTTGGTATGACCTCGGGAGGGAGGAGAACAATCATTAACTCGTATTCAGAATTCCTTACTATGATGGCTCGGAAGATGAAGGACACCGACTCCGAAGAGGAAATCCGGGAAGCTTTCAAGGTCTTCGACCGCGACAACAACGGTTTCATCTCCGCTGCGGAGCTGCGCCACGT |
| Aspergillus piperis | >NR_077191.1 Aspergillus piperis CBS 112811 ITS region; from TYPE material  CCGAGTGCGGGTCCTTTGGGCCCAACCTCCCATCCGTGTCTATTATACCCTGTTGCTTCGGCGGGCCCGCCGCTTGTCGGCCGCCGGGGGGGCGCCTTTGCCCCCCGGGCCCGTGCCCGCCGGAGACCCCAACACGAACACTGTCTGAAAGCGTGCAGTCTGAGTTGATTGAATGCAATCAGTTAAAACTTTCAACAATGGATCTCTTGGTTCCGGCATCGATGAAGAACGCAGCGAAATGCGATAACTAATGTGAATTGCAGAATTCAGTGAATCATCGAGTCTTTGAACGCACATTGCGCCCCCTGGTATTCCGGGGGGCATGCCTGTCCGAGCGTCATTGCTGCCCTCAAGCCCGGCTTGTGTGTTGGGTCGCCGTCCCCCCTCTCCGGGGGGACGGGCCCGAAAGGCAGCGGCGGCACCGCGTCCGATCCTCGAGCGTATGGGGCTTTGTCACATGCTCTGTAGGATTGGCCGGCGCCTGCCGACGTTTTCCAACCATTTTTTCCA | >MZ027925.1 Aspergillus piperis strain DTO 438-C5 beta-tubulin (benA) gene, partial cds  GGTGCTGCTTTCTGGTACGTATTCACTGCCACTGGATTGGGGATGGATAACATCATCTCTCAAGCTATCTCGGCTTGAGTTCAGATGTTATTTATCGGGTATATAGCTATCGGGTTAAGAACACGTCTAACAACTCAACAGGCAGACCATCTCTGGCGAGCACGGCCTTGACGGCTCCGGTGTGTAAGTGCAACTTTTTCACACCTCTCA  ATTGGTCGACATTGTGGAAAGGATTGGGTTTCCTGACACGCAGGATAGTTACAATGGCACCTCCGACCTCCAGCTGGAGCGCATGAACGTCTACTTCAACGAGGTTAGATCACACCGTCCCTGAGTTTTTTCACGACAATATCATCAATGTCCTGACCACTTCAGCAGGCTAGCGGTAACAAGTATGTCCCCCGTGCCGTCCTCGTCGATCTCGAGCCCGGTACCATGGACGCTGTCCGTGCCGGTCCCTTCGGCCAGCTCTTCCGTCCCGACAACTTCGTCTTCGGCCAGTCCGGTGCTGGTAACAACTGGGCCAAGGGTCACTAC | >MZ027986.1 Aspergillus piperis strain DTO 438-C5 calmodulin (cmdA) gene, partial cds  CCGAGTACAAGGAGGCCTTCTCCCTCTTTGTGAGTGCTCCCTGAATGAACTTCCGGTCACTCAAATTGATGACCTATCTTGACCGGCTCATAATGCTAATGTATTCTCGAACTCAATAGGACAAGGATGGCGATGGTGGGTGGAATTCTGTCCCCTTTACGTTTTACCTGTAGCGCTCGATCCGACCGCGGGATTTCGACAGCCATTTCCCCATCGATCTGAATCATTATACTGATGTAACTTGGAAATAGGCCAGATCACCACCAAGGAGCTCGGCACTGTGATGCGCTCCCTCGGCCAGAACCCCTCCGAGTCTGAGCTTCAGGACATGATCAACGAGGTTGACGCTGACAACAACGGAACGATCGACTTCCCCGGTATGTGATAGATCTACGCCTGTAGGGCGGGAATGCCGTATGGGTTGTGATTGACTTTTGCCGCCAGAATTCCTTACCATGATGGCTCGTAAGATGAAGGACACCGACTCCGAGGAGGAAATCCGCGAGGCTTTCAAGGTCTTCGACCGCGACAACAATGGTTTCATCTCCGCCGCGGAGTTGCGCCACGTCATGA |
| Aspergillus tanneri | >NR_111840.1 Aspergillus tanneri ATCC MYA-4905 ITS region; from TYPE material  AAGGATCATTACCGAGTGAGGGTCCCCCGGGGCCCAACCTCCCACCCGTGTCTATCGTACCTTGTTGCTTCGGCGGGCCCGCCGCTTGTTCGGCCGCCGGGGGGGCTCTCGCCCCCCGGGCCCGCGCCCGCCGGAGACCCCAACATGAACACTATGTCTGAAGCTTTGCAGTCTGAGTCGATTGTATCGCAATCAGTTAAAACTTTCAACAATGGATCTCTTGGTTCCGGCATCGATGAAGAACGCAGCGAAATGCGATAACTAATGTGAATTGCAGAATTCAGTGAATCATCGAGTCTTTGAACGCACATTGCGCCCCCTGGTATTCCGGGGGGCATGCCTGTCCGAGCGTCATTGCTGCCCTCAAGCACGGCTTGTGTGTTGGGCCGCCGTCCCCCCGGGGACGGGCCCGAAAGGCAGCGGCGGCACCGCGTCCGGTCCTCGAGCGTATGGGGCTTTGTCACCCGCTCTGCAGGCCCGGCCGGCGCTGGCCGACGCTTCACACCGAAGCAACCAACCTTCTCTCCA | >JN896577.1 Aspergillus tanneri isolate NRRL 62426 beta-tubulin gene, partial cds  TGGTACGTCAATATAGGCAATTGGAGTCTTCTGGGTCCAAATTCTTTTGAGCTTGGGCTATACAAGACCTGTTTATTTCTACGCATGAGTACTGACAATGGAAATAGGCAGACTATCTCCGGAGAGCACGGCCTTGACGGCTCCGGTGTGTAAGTACAGCCCGCCTACATTACGAAATGAGGAGCAAAAAAGGCAGTGAAAGGAGAGGCGCATAGCATGATCATGTGTCTGATGGAATGAATAGTTACAATGGCTCCTCAGACCTCCAGCTGGAGCGCATGAACGTCTACTTCAACGAGGTTCGGATGTCTCCCATATCATCCAGGAACAGGTTCTGACTTCTTTTTTCTCCCCTAGGCTTCCGGAGGCAAGTATGTTCCTCGTGCTGTTCTGGTCGACCTGGAGCCCGGCACCATGGACGCTGTCCGTGCCGGTCCCTTCGGCCAGCTTTTCCGTCCCGACAACTTCGTTTTCGGCCAGTCTGGCGCTGGTAACAACTG | >JN896578.1 Aspergillus tanneri isolate NRRL 62426 calmodulin gene, partial cds  CAGGTCTCCGAATACAAGGAGGCCTTCTCTCTGTTTGTAAGTGACCGTAATATCCTTATATAATGATGGGAATCATGTGTATTCCCCGATACTCTGCTCGAATATATGCTGACATGGGATATCCTGTCTCTGAACAGGACAAGGATGGTGATGGTTAGTGCGAAACTCCTCCCATTAAAATTTCCTCTCACACGAATGACTTTTTTACAT  TTTCATAAATTTTTCTTATACTTTTGTCGACTCGCAACATCATACTTACAACCTAATTCATCAATGTGCAGGCCAGATCACCACCAAGGAGCTGGGCACTGTTATGCGCTCGCTGGGCCAAAACCCTTCCGAGTCCGAGTTGCAGGATATGATTAACGAAGTTGACGCTGACAACAATGGCACTATCGACTTCCCTGGTATGAGATGGGC  ACACCGTAATCCGCAAGGGAGAAGATCTGCTGCTAACCGGTCAAAGAATTCCTCACGATGATGGCCCGAAAGATGAAAGATACTGATTCCGAGGAGGAAATCCGGGAAGCTTTCAAGGTCTTCGATCGCGACAACAATGGTTTCATTTCTGCCGCGGAGTTGCGTCATGTCATGACTTCCATCGGCGAGAAACTTACCGATGACGAAGTCGACGAGATGATTCGTGAGGCGGACCAGGATGGCGATGGCCGGATTGACTGTACGTTGCCTCGTGTCTTACCCTTTACTTGAGATGAAGCTAATTGATCTCCCAGACAACGAGTTT |
| Aspergillus luchuensis | >NR_135449.1 Aspergillus luchuensis KACC 46772 ITS region; from TYPE material  AAGGATCATTACCGAGTGCGGGTCCTTTGGGCCCAACCTCCCATCCGTGTCTATTATACCCTGTTGCTTCGGCGGGCCCGCCGCTTGTCGGCCGCCGGGGGGGCGCCTTTGCCCCCCGGGCCCGTGCCCGCCGGAGACCCCAACACGAACACTGTCTGAAAGCGTGCAGTCTGAGTTGATTGAATGCAATCAGTTAAAACTTTCAACAATGGATCTCTTGGTTCCGGCATCGATGAAGAACGCAGCGAAATGCGATAACTAATGTGAATTGCAGAATTCAGTGAATCATCGAGTCTTTGAACGCACATTGCGCCCCCTGGTATTCCGGGGGGCATGCCTGTCCGAGCGTCATTGCTGCCCTCAAGCCCGGCTTGTGTGTTGGGTCGCCGTCCCCCTCTCCGGGGGGACGGGCCCGAAAGGCAGCGGCGGCACCGCGTCCGATCCTCGAGCGTATGGGGCTTTGTCACATGCTCTGTAGGATTGGCCGGCGCCTGCCGACGTTTTCCAACCATTTTTTCCA | >MN510418.1 Aspergillus luchuensis isolate 16676/C beta-tubulin (benA) gene, partial cds  GTGCTGCTTTCTGGTACGTATTCACTGCCACTGGATTGGGGATGGATAACATCATCTCTCAAGCTATCTC  GGCTTGAGTTCAGATGTTATTTATCGGGTATATAGCTATCGGGTTAAGAACACGTCTAACAACTCAACAG  GCAGACCATCTCTGGCGAGCACGGCCTTGACGGCTCCGGTGTGTAAGTGCAACTTTTTCACACCTCTCAA  TTGGTCGACAATGTGGAAAGGATTGGGTTTCCTGACACGCAGGATAGTTACAATGGCACCTCCGACCTCC  AGCTGGAGCGCATGAACGTCTACTTCAACGAGGTTAGATCACACCGTCCCTGAGTTTTTTCACGACAATA  TCATCAATGTCCTGACCACTTCAGCAGGCTAGCGGTAACAAGTATGTCCCCCGTGCCGTCCTCGTCGATC  TCGAGCCCGGTACCATGGACGCTGTCCGTGCCGGTCCCTTCGGCCAGCTCTTCCGTCCCGACAACTTCGT  CTTCGGCCAGTCCGGTGCTGGTAACAACTGGGCCAAGGGTCACTA | >JX500074.1 Aspergillus luchuensis isolate KACC 45131 calmodulin (CAM) gene, partial cds  GCAAGTTTCTGAGTACAAGGAGGCCTTCTCCCTCTTTGTGAGTGCTCCCTGAATAAACCCCCGATCACTCAAATTGATGACCTATCATGACCGGCTCATAATGCTAATGTATTCTCGAACTCAATAGGACAAGGATGGCGATGGTGGGTGGAATTCTGTCCCCTTTACGTTTTACCCGTAGCGCCCGATCCGACCGCGGGATTTCGACAG  CCATTTCCCCATCGATCTGAATCATTATACTGATGTAATCTGGAAATAGGCCAGATCACCACCAAGGAGCTCGGCACTGTGATGCGCTCCCTCGGCCAGAACCCCTCCGAGTCTGAGCTTCAGGACATGATCAACGAGGTTGACGCTGACAACAACGGAACGATCGACTTCCCCGGTATGTGATAGATCTACGCCTGTAGGGCGGGAATGCCGTATGGGTTGTGATTGACTTTTGCCGCCAGAATTCCTTACCATGATGGCTCGTAAGATGAAGGACACCGACTCCGAGGAGGAAATCCGCGAGGCTTTCAAGGTCTTCGACCGCGACAACAATGGTTTCATCTCCGCCGCGGAGTTGCGCCACGTCATGACCTCCATTGGCGAGAAGCTCACTGACGACGAAGTCGATGAGATGATCCGTGAGGCTGACCAGGATGGTGATGGCCGCATCGACTGTATGTTTCCCATTCTTGATATGCCCGTGATATAGCATGCTAACTCTGCTACCAGACAACGAGTTCG |
| Aspergillus mulundensis | >NR_159553.1 Aspergillus mulundensis DSM 5745 ITS region; from TYPE material  TCCGTAGGGGTGACCTGCGGAAGGATCATTACCGAGTGCGGGCTGCCTCCGGGCGCCCAACCTCCCACCCGTGACTACCTAACACTGTTGCTTCGGCGGGGAGCCCCCCCTGGGGGGCGAGCCGCCGGGGACCACCGAACTTCATGCCTGAGAGTAATGCAGTCTGAGTCTGAATACAAATCAGTCAAAACTTTCAACAATGGATCTCTTGGTTCCGGCATCGATGAAGAACGCAGCGAACTGCGATAAGTAATGTGAATTGCAGAATTCAGTGAATCATCGAGTCTTTGAACGCACATTGCGCCCCCTGGCATTCCGGGGGGCATGCCTGTCCGAGCGTCATTACTGCCCTTCAAGCGCGGCTTGTGTGTTGGGTCCTCGTCCCCCCCGGGGGACGGGCCCGAAAGGCAGCGGCGGCACCGTGTCCGGTCCTCGAGCGTATGGGGCTTTGTCACCCGCTCGATTAGGGCCGGCCGGGCGCCAGCCAGCGTCAACTCCTTCTTAA | >KU866833.1 Aspergillus mulundensis strain DTO 316-C9 beta-tubulin (BenA) gene, partial cds  GGTACGTCAAAAATTTAAACGTCTTGGGATACTCTTTCCCCGACACTTGCTAATAAATTTATAGGCAGACCATCTCCGGTGAGCACGGCCTCGATGGCTCCGGTGTGTGAGTACAATCCGTCCAGAGTCCGATAACAACACAACGAGCGAACGGGACCTGATACAAAGCAGTTACAATGGTACCTCCGACCTCCAGCTCGAGCGTATGAACGTCTACTTCAACGAGGTCAGTGGATAAGCTGCAGCGGTGGCAATTGAGGCATCTCTGACTCTTTGCAGGCCAGCGGTAACAAGTACGTTCCCCGTGCCGTCCTCGTCGATCTCGAGCCCGGTACTATGGACGCCGTCCGTGCCGGTCCCTTCGGCGAGCTCTTCCGTCCCGACAACTTCGTTTTCGGTCAGTCCGGTGCTGGTAACAACTGGGCCAAGGGT | >KU866729.1 Aspergillus mulundensis strain DTO 316-C9 calmodulin gene, partial cds  GTAAGTGGCATTGATTACTTCCTCGTCAGCACGAGAATTTCCTTGGAATATACTAATCACTCCGTATTTATAGGACAAGGATGGCGATGGTTAGTGCATTTGCCCCCTACAACATGATCGCATTTATCCGGCTTTGGTGTTATGTCTGTATATCGACTGATTTTAACGAATGACGCTAGGACAAATCACCACCAAGGAACTCGGTACCGTCATGCGCTCGCTCGGCCAGAACCCTTCGGAGTCTGAACTCCAGGACATGATCAACGAAGTTGACGCCGACAACAATGGCACCATTGACTTCCCAGGTGCGTGATTTACTTGCAACACCCAATGCACCGCGTTGAAGAAAATGGGCTCTAACTGGAAAATAGAGTTCCTCACCATGATGGCCAGAAAGATGAAGGACACCGACTCCGAGGAGGAAATCCGAGAGGCGTTCAAAGTCTTTGACCGCGACAACAATGGTTTTATCTCCGCTGCTGAGCTGCGCCACGT |
| Aspergillus sclerotioniger | >NR_077192.1 Aspergillus sclerotioniger CBS 115572 ITS region; from TYPE material  CCGAGTGCGGGTCCTTTGGGCCCAACCTCCCACCCGTGTCTATTGTACCCTGTTGCTTCGGCGGGCCCGCCGCTTGTCGGCCGCCGGGGGGGCATCTCTGCCCCTCGGGCCCGTGCCCGCCGGAGACACCAACACGAACACTGTCTGAAATCGTGAAGTCTGAGTCGATTGTTTTCAATCAGTTAAAACTTTCAACAATGGATCTCTTGGTTCCGGCATCGATGAAGAACGCAGCGAAATGCGATAACTAATGTGAATTGCAGAATTCAGTGAATCATCGAGTCTTTGAACGCACATTGCGCCCCCTGGTATTCCGGGGGGCATGCCTGTCCGAGCGTCATTGCTGCCCTCAAGCCCGGCTTGTGTGTTGGGTCGCCGTCCCCCTGTCTGGGGGGACGGGCCCGAAAGGCAGCGGCGGCACCGCGTCCGATCCTCGAGCGTATGGGGCTTTGTCACATGCTCTGTAGGATTGGCCGGCGCCTGCCGACAACTCCAACCTTTTTTTCCA | >GU296702.1 Aspergillus sclerotioniger strain CBS 115572 beta-tubulin (tub2) gene, partial cds  AGGCAGACCATCTCTGGTGAGCACGGCCTCGACGGCTCCGGTGTGTAAGTACACCCTTTTTACGTCTCAATCGGTCAACATGATGGAAGAACCGGGTTCCTGACGTGGAGGATAGTTACAATGGCACCTCCGATCTCCAGCTGGAGCGCATGAACGTCTACTTCAACGAGGTGGGTCTCACCCAATCCTAGGCTATACCACGTGTACGTGTGGTGTCTCGATTCCTGACCCCTTCACCAGGCTAGCGGCAACAAGTATGTTCCTCGTGCCGTCCTTGTCGACCTTGAGCCCGGTACCATGGATGCCGTCCGTGCCGGTCCTTTCGGACAGCTCTTCCGCCCCGACAACTTCGTCTTCGGCCAGTCCGGTGCTGGTAACAACTGG | >EU163271.1 Aspergillus sclerotioniger calmodulin (cmd) gene, partial cds  GATAAAATATTGATAGGACAAGGATGGCGATGGTGGGTGGAACCCTATTCCCTTCGTGTTTACCTATGGCGACCGAACCGACCGCGGGTTTCCGAAAGCTGTTTTTTGATCGACTTGAGCCGTAATACTGATGTGACTTGGGAAATAGGCCAGATCACCACCAAGGAGCTGGGCACTGTCATGCGCTCGCTCGGCCAGAACCCCTCCGAGTCTGAGCTTCAGGATATGATTAACGAGGTTGACGCTGACAACAACGGCACGATCGACTTCCCTGGTATGTGGCGGGCTTATGTTTATACGAAGCGGGAATGCGGATCGGGTATTAACTTCTGCCAGAATTCCTCACTATGATGGCTCGTAAGATGAAAGACACCGACTCCGAGGAGGAGATCCGCGAGGCTTTCAAGGTCTTCGACCGCG  ATAACAACGGTTTTATCTCCGCTGCTGAGCT |
| Aspergillus ustus | >NR_131284.1 Aspergillus ustus CBS 261.67 ITS region; from TYPE material  GGAAGGATCATTACCGAGTGCAGGTCTGCCCCCGGGCAGGCCTAACCTCCCACCCGTGAATACCTGACCAACGTTGCTTCGGCGGTGCGCTCCCCCCGGGGGCAGCCGCCGGAGACCACACCGAACCTCTTGTTATAGCGTGTCGTCTGAGCTTGATACAAGCAAACCTAATTAAAACTTTCAACAATGGATCTCTTGGTTCCGGCATCGATGAAGAACGCAGCGAACTGCGATAAGTAATGTGAATTGCAGAATTCAGTGAATCATCGAGTCTTTGAACGCACATTGCGCCCCCTGGCATTCCGGGGGGCATGCCTGTCCGAGCGTCATTGCTGCCCTTCAAGCCCGGCTTGTGTGTTGGGTCGTCGTCCCCCCCGGGGGACGGGCCCGAAAGGCAGCGGCGGCACCGCGTCCTGGTCCTCGAGCGTATGGGGCTTTGTCACCCGCTCGTTTAGGGCCGGCCGGGCGCCAGCCGGCGTCTCCAAACCTTTTATTTTACCAGGTTGACCTCG | >EF652279.1 Aspergillus ustus isolate NRRL 275 beta-tubulin gene, partial cds  TGGTACGTCGAAAAATTCCCTCAGCGATTGTTTGCTTCTAGGTTGTATCTCTGCTGACACGTTTACAGGC  AAACCATCTCCGGTGAGCACGGCCTTGATGGCTCCGGTGTGTGAGTGCAACCCGTTTCGACCCCGACGAA  AATACAATGGAACGGAAACCTGATGGGATACAGTTACAATGGCTCCTCCGACCTCCAGCTCGAGCGTATG  AACGTCTACTTCAACGAGGTCAGTGTTTCGACCATAAGACGAACAGCTTGGTGGTGTGTCTGACTCGATA  CAGGCCAGCGGCAACAAGTACGTTCCCCGTGCTGTCCTCGTCGATCTCGAGCCCGGTACTATGGACGCTG  TCCGTGCCGGTCCCTTCGGCCAGCTTTTCCGTCCCGACAACTTCGTCTTCGGCCAGTCTGGTGCTGGTAA  CAACTGG | >EF652367.1 Aspergillus ustus isolate NRRL 275 calmodulin gene, partial cds  GTTTCCGAGTACAAGGAAGCATTCTCTCTATTTGTAAGTCCCATATGGTATTACATAAACTACCGGTCGCCATTTAAGAATCACTAACTTACCCTTTTCCGCGTGAAACAGGACAAGGATGGCGATGGTTAGTGCAATTCTCGCGCCCATTTATTCCGGCGATGTTATCGCATACGCCCTAAAAATATCCAGGAAATTTGGTCGCATTTACTGCTCTGGTATTAATATATGCGTGTAGGCCAGATCACCACCAAGGAACTCGGCACTGTGATGCGCTCACTCGGCCAGAATCCCTCCGAGTCCGAACTTCAGGACATGATCAACGAGGTCGACGCCGACAACAATGGCACCATTGACTTTCCAGGTATTCTATTCGGCTGTTTTTTTCGTGTGCGATACAATTGGGCAGATCACTGATATTCAAACCAGAGTTCCTCACAATGATGGCCAGAAAGATGAAGGACACCGACTCCGAGGAGGAAATCCGCGAGGCGTTCAAGGTTTTCGACCGTGACAATAATGGTTTCATCTCGGCTGCCGAGCTACGCCATGTCATGACCTCTATTGGCGAGAAGCTCACCGATGATGAAGTCGATGAGATGATCCGTGAGGCAGACCAGGATGGTGACGGCCGAATTGACTGTACGTTGACCGTTCCAGTTATACAGTTATACTCGATGTGCAAGAATTGATGCTAATAATTCTTCAGACAACGAATTC |
| Aspergillus sergii | >NR_172033.1 Aspergillus sergii MUM 10.219 ITS region; from TYPE material  CATTACCGAGTGTAGGGTTCCTAGCGAGCCCAACCTCCCACCCGTGTTTACTGTACCTTAGTTGCTTCGGCGGGCCCGCCGTCATGGCCGCCGGGGGCGTCAGCCCCGGGCCCGCGCCCGCCGGAGACACCACGAACTCTGTCTGATCTAGTGAAGTCTGAGTTGATTGTATCGCAATCAGTTAAAACTTTCAACAATGGATCTCTTGGTTCCGGCATCGATGAAGAACGCAGCGAAATGCGATAACTAGTGTGAATTGCAGAATTCCGTGAATCATCGAGTCTTTGAACGCACATTGCGCCCCCTGGTATTCCGGGGGGCATGCCTGTCCGAGCGTCATTGCTGCCCATCAAGCACGGCTTGTGTGTTGGGTCGTCGTCCCCTCTCCGGGGGGGACGGGCCCCAAAGGCAGCGGCGGCACCGCGTCCGATCCTCGAGCGTATGGGGCTTTGTCACCCGCTCTGTAGGCCCGGCCGGCGCTTGCCGAACGCAAAACAATCTTTCCA | >MG517688.1 Aspergillus sergii strain DTO 223-C9 beta-tubulin (BenA) gene, partial cds  TGGTATGTCTCCATACCTTCGAGTTAATATGCTTTGGATCAAGGAACTCCTCAAAAGCATGATCTCGGATGTGTCCTATTATATCTGCCACGTGTTTGCTAACAACTTTGCAGGCAAACCATCTCTGGCGAGCACGGCCTTGACGGCTCCGGTGTGTAAGTACAGCCTGTATACACCTCGAACGAACGACGACCATATGGCATTAGAAGTTGGAATGGATCTGACGGCAAGGATAGTTACAATGGCTCCTCCGATCTCCAGCTGGAGCGTATGAACGTCTACTTCAACGAGGTGCGTACCTCAAAATTTTCAGCATCTATGAAAACGCTTTGCAACTCCTGACCGCTTCTCCAGGCCAGCGGAAACAAGTATGTCCCTCGTGCCGTCCTCGTCGATCTTGAGCCTGGTACCATGGACGCCGTCCGTGCCGGTCCCTTCGGTCAGCTCTTCCGTCCCGACAACTTCGTTTTCGGCCAGTCCGGTGCTGGTAACAACTGGGCCAAG | >MG518059.1 Aspergillus sergii strain DTO 223-C9 calmodulin (CaM) gene, partial cds  TCGTAAGTAGTTATCGTCGTTCGCGAAAATTGGTTTTTGTTAGTCGTCTTGATTTGAACACAAGCTGACTTGGCTTTTCTTGGGTTTCCTATAGGACAAGGACGGTGATGGTTAGTACAGTTTATTTTATTCATTCTCCCTTCAAATGCGACCAATATCTTTTAGCCGCCATAAGTTTATCAAGTTTCTGTTCGATCGGCTGAAGTCTTGGCATTGATGAATTGACTTGATATGCAGGCCAGATCACCACCAAGGAGTTGGGCACTGTCATGCGCTCTCTGGGTCAAAACCCCTCTGAGTCGGAACTCCAGGACATGATTAACGAGGTTGACGCCGACAACAATGGCACCATTGACTTCCCTGGTACGAGACGGCTTCCGTACGATTCATAGATGAAATAGTTGTTAATCGTCCAAATAGAATTCCTTACGATGATGGCGAGAAAGATGAAGGATACCGACTCTGAGGAGGAGATCCGGGAGGCTTTCAAGGTTTTCGACCGCGATAACAACGGCTTCATCTCCGCTGCCGAA |
| Aspergillus calidoustus | > Aspergillus calidoustus ITS  TCCGTAGGTGAACCTGCGGAAGGATCATTACCGAGTGCAGGTCTGCCCCCGGGCAGGCCTAACCTCCCACCCGTGAATACCTGACCAACGTTGCTTCGGCGGTGCGCCCCCCCCGGGGGTAGCCGCCGGAGACCACACCGAACCTCCTGTCTTTAGTGTTGTCTGAGCTTGATAGCAAACCTATTAAAACTTTCAACAATGGATCTCTTGGTTCCGGCATCGATGAAGAACGCAGCGAACTGCGATAAGTAATGTGAATTGCAGAATTCAGTGAATCATCGAGTCTTTGAACGCACATTGCGCCCCCTGGCATTCCGGGGGGCATGCCTGTCCGAGCGTCATTGCTGCCCTTCAAGCCCGGCTTGTGTGTTGGGTCGTCGTCCCCCCCGGGGGACGGGCCCGAAAGGCAGCGGCGGCACCGCGTCCGGTCCTCGAGCGTATGGGGCTTTGTCACCCGCTCGATTAGGGCCGGCCGGGCGCCAGCCGGCGTCTCCAACCTTCTATTTTACCAGGTTGACCTCGGATCAGGTAGGGATACCCGCTGAACTTAAGCATATCAATAAGCGGAGGAAAAGA | >MT410164.1 Aspergillus calidoustus isolate CCMFBH-940 beta-tubulin (benA) gene, partial cds  TGCTGCTTTCTGGTATGTCGAAAATTTTCGGCCAACAGTTGTTTGCTTCCGCGTTGTATCGGTGCTGACACGGTTCTAGGCAAACCATCTCTGGTGAACACGGCCTCGATGGCTCTGGTGTGTGAGTGCACCCTCTCTTGGACGCCGACGGAAAACACAACGGAACAGAAACCTGATGGGAAGCAGTTACAATGGCACCTCCGACCTCCAGCTCGAGCGTATGAACGTCTACTTCAACGAGGTCAGTGTTGCGGATATAAAACAAATGGCTTGGTTGTGTGTCTAACTTTGTCCAGGCCAGCGGCAACAAGTACGTTCCCCGTGCCGTCCTCGTCGATCTCGAGCCCGGCACCATGGACGCCGTCCGCGCCGGTCCCTTCGGCCAGCTCTTCCGCCCCGACAACTTCGTCTTCGGCCAGTCCGGTGCTGGTAACAACTGGGCCAAGGGTCAA | >HG964950.1 Aspergillus calidoustus partial CaM gene for calmodulin protein, isolate E460  GTCTCCGAGTACAAGGAGGCCTTCTCCCTATTTGTAAGTCGCCCACGAAATCTTATGATCACCGATCGCTATCTAAGAATCCCTTATTTACCCTTTTCCGCTTTGAACAGGACAAGGATGGTGATGGTTAGTGCAACCCACCCACTCCGGCAACGTTATCGCATACGCTCAGCCTCGATTATCCCATAATGTTTAGCCCCATCAATTAGCCTGGTATTGATAATGTGTGAATGCGTTTAGGCCAGATTACCACCAAGGAACTCGGCACTGTGATGCGCTCGCTCGGGCAGAACCCCTCCGAGTCTGAACTCCAGGACATGATCAACGAGGTTGACGCCGACAACAACGGCACCATTGATTTCCCAGGTATATACTCACGATACAACCTCTAGGCTTACAGGATATTGGGCAGATCACTGACTCGAAAAAATATATCAGAGTTCCTCACAATGATGGCCAGAAAGATGAAGGACACCGACTCCGAGGAGGAAATCCGAGAGGCTTTCAAGGTTTTCGACCGTGACAACAATGGTTTCATCTCAGCTGCCGAACTGCGCCACGTYATGACCTCYATCGGCGA |

| Species | Target | Oligo Sequence (5’→3’) |
| --- | --- | --- |
| *Aspergillus fumigatus* | TTTGTGAAAGCAGCGACTTGGGTTA | F: GGTTGCAGCCAGCAGCAACATTCTT |
|  |  | R: GCCTTAACCTGCAGCAGCAGCGTTT |
| *Aspergillus terreus* | TTTCAGTGGCAGTAAGAGTAGTCTC | F: ACCGCCAGTTTGTCGTCTCCACACC |
|  |  | R: TCGAAAGGCATTCGAGAAGAGACGC |
| *Aspergillus nidulans* | TTTCCCAGTCTTAAGCTGGAATCTG | F: AGCCTCTCCTGAGTATGTTATTCGG |
|  |  | R: GTCGCCTAGGTACATGTCTCATAGC |
| *Aspergillus niger* | TTTGAATGCTGAACCAGAGCACGAT | F: ATAACAAGGAAGCCAGCGCTTACGC |
|  |  | R: GATATAGCATGCATAGTCTGCGTCC |
| *Aspergillus flavus* | TTTCGTCCGACTTTAGAATGAGTTG | F: GGTGAGGCTAATCAGTAAGTTCCGC |
|  |  | R: GCATACCTCCAAGGCACAGTTCCAT |
| *Aspergillus parasiticus* | TTTGCTAAAAGGCCATTGAGGCGAG | F: ATTGACTCGTGGATGAATTGGACCG |
|  |  | R: ATGAGCTTAAGGAACGGCTTCGAGC |
| *Aspergillus oryzae* | TTTCCATTTACTTTTAAGGCAGGGG | F: ATCAGCGTCAGATTGTTCATGGGCC |
|  |  | R: AGCATACGGAACTTTTCCGCAAGCG |

**Table S3 The primer pairs of species Targets.**
